# ADVANCE-ID bacterial and fungal infections review: antimicrobial resistance research updates: Summary of clinical trials and implementation studies, March–April 2026

**DOI:** 10.1093/jacamr/dlag113

**Published:** 2026-06-22

**Authors:** Yin Mo, Abi Manesh, Jennifer Wang, David L Paterson

**Affiliations:** ADVANCE-ID, Saw Swee Hock School of Public Health, National University of Singapore, Singapore; Saw Swee Hock School of Public Health, National University of Singapore and National University Health System, Singapore; Infectious Diseases Translational Research Programme, Yong Loo Lin School of Medicine, National University of Singapore, Singapore; Mahidol-Oxford Tropical Medicine Research Unit, Mahidol University, Bangkok, Thailand; ADVANCE-ID, Saw Swee Hock School of Public Health, National University of Singapore, Singapore; Department of Infectious Diseases, Christian Medical College (CMC) Vellore, Vellore, India; ADVANCE-ID, Saw Swee Hock School of Public Health, National University of Singapore, Singapore; ADVANCE-ID, Saw Swee Hock School of Public Health, National University of Singapore, Singapore; Infectious Diseases Translational Research Programme, Yong Loo Lin School of Medicine, National University of Singapore, Singapore

ADVANCE-ID (Advancing Clinical Trials in Infectious Diseases) is a clinical trial network that aims to generate clinically relevant, policy-informing evidence for infectious diseases, with a focus on antimicrobial resistance (AMR). By building large, multicentre interventional trials across diagnostics, therapeutics and prevention strategies, the network is designed not only to generate evidence but also to enable its translation into policy and clinical practice across diverse healthcare settings. In this series, we provide highlights from our biweekly *Bacterial and Fungal Infections Newsletter* in the past 2 months (March*–*April 2026) featuring recent studies that illustrate how evidence in AMR is evolving, and where key challenges remain in bridging data to decision-making. To receive the bi-weekly newsletter, sign up here: https://ad-id.co/subscribe

**Implementation and setting-specific interventions are key in antimicrobial stewardship programmes.** A large cluster-randomized trial across rural township hospitals in China showed that a digitally enabled stewardship intervention reduced antibiotic prescribing for acute respiratory infections from 71% to 26% without increasing 30-day hospitalization^1^. Its scale and pragmatic design support real-world applicability, demonstrating that prescribing behaviour can be modified at scale in the community setting. In contrast, the multicentre PRONTO randomized controlled trial in emergency departments in England and Wales found that procalcitonin-guided algorithms did not reduce early antibiotic initiation though lower mortality was demonstrated in the procalcitonin arm^2^. This divergence reflects differences in patient population and implementation: in rural China, stewardship targeted less severely ill patients and used integrated tools such as electronic prompts, training and feedback, whereas PRONTO evaluated a single, variably adopted diagnostic in sicker patients with greater clinical uncertainty. As a standalone intervention without reinforcement, it did not change early antibiotic use.

***Clostridioides difficile* and the microbiome: towards targeted ecological therapy.** Trials in *C. difficile* infection highlight the limits of optimizing antibiotic regimens alone. A randomized trial of vancomycin tapering showed only modest and uncertain reductions in recurrence, with early signals of benefit not sustained and limited by early termination^3^. Similarly, a phase 2 trial of CRS3123, a protein synthesis inhibitor, demonstrated comparable initial cure and lower recurrence signals relative to vancomycin, suggesting the potential of more selective, microbiome-sparing agents^4^. These trials offered further evidence that *C. difficile* recurrence is a consequence of disrupted microbial ecology rather than incomplete pathogen clearance. Emerging approaches such as CRISPR–phage therapies introduce a novel mechanism, as shown by Peterson *et al*.^5^, using a bacteriophage cocktail engineered to selectively target *Escherichia coli* in the gut. In a first-in-human study, this approach demonstrated favourable safety, dose-dependent recovery in stool and gut-restricted activity without systemic exposure or disruption of overall microbiome composition. Although reductions in *E. coli* burden were modest, this targeted approach supports further development of precision microbiome-directed interventions.

**Infection prevention in low- and middle-income countries: a much-needed evidence gap.** The Clean FrontLine stepped-wedge cluster trial in Cambodian hospitals addresses a critical gap in antimicrobial resistance: the role of infection prevention and control (IPC) in low-resource settings, where the burden of healthcare-associated infections is high and infrastructure constraints are substantial^6^. A multicomponent training intervention, embedding cleaning champions and structured supervision, improved microbiological surface cleanliness at the hospital level, demonstrating that context-appropriate IPC interventions are feasible and scalable. Such studies are urgently needed in LMICs, where IPC remains under-resourced despite its central role in reducing transmission of resistant pathogens. However, the primary endpoint was a surrogate measure, and the modest absolute improvements leave uncertainty around downstream effects on infection rates and patient outcomes. To translate IPC into policy and sustained practice change, future studies will need to incorporate clinical endpoints, alongside implementation science and health economic evaluations to define scalable, cost-effective best practices.

## Supplementary Material

dlag113_Supplementary_Data

## Data Availability

DLP has received grants from Shionogi, Pfizer, Merck, BioVersys, Gilead and bioMerieux. He has received honoraria as a speaker or consultant to the AMR Action Fund, CARB-X, GARDP, Shionogi, Merck, Aurobac, Antabio, BioVersys, Basilea, Gangagen, Menarini, ADVANZ, Roche, GSK and Tamrisa.
