# Supplementary material for: ADVANCE-ID bacterial and fungal infections review: antimicrobial resistance research updates: Summary of clinical trials and implementation studies, March–April 2026
Source: JAC Antimicrob Resist. 2026 Jun 22;8(3):dlag113. doi: 10.1093/jacamr/dlag113 (PMC13284420; doi:10.1093/jacamr/dlag113)
Supplement: dlag113_Supplementary_Data [file dlag113_supplementary_data.docx]

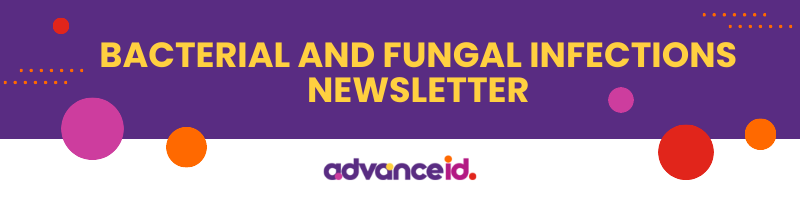


**Randomised Controlled Trials^[[1]](#footnote-1)^**

| **Citation of Articles** | **PICO** | **Main Results** | **Risk of Bias** |
| --- | --- | --- | --- |
| [Liatsikos K, Hyder-Wright A, Davies K, El Safadi D, Farrar M, Goncalves A, et al. The effect of pneumococcal conjugate vaccine and pneumococcal polysaccharide vaccine on nasopharyngeal colonisation following human infection challenge with serotype 3 and serotype 6B (PREVENTING PNEUMO 2): a double-masked, randomised, controlled, phase 4 trial. Lancet Microbe. 2026 Feb 13; doi:10.1016/j.lanmic.2025.101267](https://ad-id.co/3ZZRNjX) | **P:** 407 healthy adults aged 18–50 years challenged with *Streptococcus pneumoniae* serotype 3 (Spn3) and 243 challenged with serotype 6B (Spn6B) in a phase 4 RCT in the UK  **I:** 13-valent pneumococcal conjugate vaccine (PCV13)  **C:** 23-valent pneumococcal polysaccharide vaccine (PPV23) or placebo (0.9% NaCl)  **O:** Acquisition of pneumococcal colonisation (Spn3 at 1 month; Spn6B at 6 months) detected by nasal wash culture | At 1 month, PCV13 showed a non-significant 16% reduction in overall Spn3 colonisation compared with placebo (56% vs 65%; RR 0.84, 95% CI 0.70–1.01; p=0.068). Protection varied by clade: PCV13 reduced clade II acquisition by 29% (RR 0.71, 95% CI 0.54–0.91; p=0.0090) but showed no protection against clade Iα (RR 1.01, 95% CI 0.77–1.32; p=0.95). PPV23 provided no protection against clade Iα (RR 0.84, 95% CI 0.63–1.11; p=0.22). At 6 months, PCV13 reduced Spn6B colonisation by 60% (RR 0.40, 95% CI 0.22–0.69; p=0.0020), whereas PPV23 showed no effect (RR 0.90, 95% CI 0.60–1.34; p=0.60). No severe or life-threatening adverse events occurred. | Low to moderate risk: The double-masked randomized design with laboratory and participant blinding reduces performance and detection bias. Modified intention-to-treat analysis strengthens validity. However, subgroup selection for the 6-month challenge was first-come-first-served, introducing potential selection bias. The study was funded by industry, which may introduce potential sponsorship bias, although outcomes were objective microbiological measures. Overall internal validity is strong but generalizability is limited to healthy young adults. |
| [Gon G, Ma S, Aiken AM, Dancer SJ, Graham WJ, Nash S, et al. Impact of a multicomponent training intervention (Clean FrontLine) on microbiological cleanliness in Cambodian referral hospitals: a multicentre, stepped-wedge, cluster-randomised trial. Lancet Microbe. 2025 Dec 10;7(2):101262; doi:10.1016/j.lanmic.2025.101262](https://ad-id.co/404mBQA) | **P:** 13 Cambodian referral hospitals (3822 near-patient surface samples analysed from 3900 collected) in a stepped-wedge cluster RCT  **I:** Clean Frontline multicomponent hospital cleaning training intervention (selection, training, and supervision of cleaning champions and cleaners)  **C:** Usual environmental cleaning practices (pre-intervention control period)  **O:** Microbiological cleanliness of near-patient surfaces (<2.5 CFU/cm²) measured by dipslides | In surface-level analysis, the intervention showed a positive but non-significant improvement in cleanliness (OR 1.39, 95% CI 0.95–2.03; p=0.081). Hospital-level analysis demonstrated a significant absolute increase in cleanliness of 5.04 percentage points (95% CI 0.76–9.33; p=0.026). Overall, the training intervention modestly improved microbiological cleanliness, with stronger evidence at the hospital level than at the individual surface level. | Moderate risk: The stepped-wedge cluster design strengthens internal validity but may introduce temporal confounding despite adjustment for predefined covariates. Masking of outcome assessors reduces detection bias. The small number of clusters (13 hospitals) limits statistical power and precision. Behavioural intervention delivery may introduce performance variability across sites. Overall risk is moderate with reasonable methodological rigor for a pragmatic implementation trial. |
| [McDonald EG, Butler-Laporte G, Brophy JM, Elsayed S, Frenette C, Huseen I, et al. Initial vancomycin taper for the prevention of recurrent Clostridioides difficile infection: a randomized clinical trial. JAMA Netw Open. 2026 Feb 27;9(2):e2560495; doi:10.1001/jamanetworkopen.2025.60495](https://ad-id.co/4shCLSD) | **P:** 265 adults with first episode or first recurrence of *Clostridioides difficile* infection (median age 63 years; 52.1% women) in 12 Canadian hospitals  **I:** 4-week vancomycin pulse and taper regimen (14-day pulse followed by 7 days twice daily and 7 days once daily)  **C:** Standard 2-week vancomycin pulse regimen plus placebo taper  **O:** Recurrent CDI by day 56 (primary); recurrence at day 38 (secondary); adverse events | Recurrence by day 56 occurred in 14.8% (20/135) in the pulse-plus-taper group versus 17.7% (23/130) in the standard pulse group (adjusted RR 0.84, 95% CrI 0.48–1.45; posterior probability of superiority 73.8%). At day 38, recurrence was lower with pulse-plus-taper (6.7% vs 15.4%; adjusted RR 0.43, 95% CrI 0.19–0.89; posterior probability of superiority 99.0%). Adverse effects were rare in both groups. The trial was stopped early due to recruitment feasibility. | Moderate risk: Double-blind design with concealed allocation and blinded adjudication reduces performance and detection bias. Early trial termination for recruitment feasibility limits power and precision, increasing risk of imprecision bias. Bayesian analysis with minimally informative priors is appropriate but wide credible intervals reflect uncertainty. Overall internal validity is good, but early stopping limits robustness of conclusions. |
| [Rose M, Vogrin S, Mitri EA, De Luca J, Lapirow D, Reynolds GK, et al. Feasibility trial of prolonged versus single-dose challenge in penicillin allergy. NEJM Evid. 2026 Feb 24;5(3); doi:10.1056/EVIDoa2500038](https://ad-id.co/47kWI2L)   - **Editorial Commentary:**   [Alvarez-Arango S, Blumenthal KG. Feasibility of PROSPECTOR — a randomized trial evaluating oral challenge duration in penicillin allergy evaluation. NEJM Evid. 2026 Feb 24;5(3); doi:10.1056/EVIDe2500223](https://ad-id.co/4sayZdy) | **P:** 120 adults with a history of delayed-onset or unknown-timing penicillin allergy who tolerated a single-dose amoxicillin challenge (screened n=302) across four Australian hospitals  **I:** 5-day multidose oral amoxicillin challenge  **C:** Placebo (after initial tolerated single-dose challenge)  **O:** Feasibility outcomes (≥80% adherence, unblinding, recruitment rate); immune-mediated adverse events up to day 7 | Adherence ≥80% was achieved in 83% overall (88% intervention vs 78% control; 95% CI 75–90), with no unblinding events and a recruitment feasibility rate of 71% (120/169 eligible participants), meeting predefined feasibility criteria. Positive immune-mediated reactions occurred in 12% (7/60) in the multidose group vs 5% (3/60) in the placebo group (risk difference 6.7 percentage points, 95% CI −3.2 to 16.5). One serious adverse event (1.7%, severe cutaneous adverse reaction) occurred in the placebo group. The study was not powered to detect efficacy differences. | Moderate risk: The multicenter, double-blind, placebo-controlled randomized design reduces performance and detection bias. However, this was a feasibility trial with a small sample size and not powered for clinical efficacy outcomes, leading to imprecision in adverse event estimates. Participants had already tolerated a single-dose challenge, limiting generalizability to higher-risk populations. Overall internal validity is good, but conclusions regarding safety differences remain uncertain. |
| [Wei X, Zhuo C, Hicks JP, Zhang Z, Wu S, Walley JD, et al. Effects of a comprehensive antibiotic stewardship program on antibiotic prescribing for acute respiratory infections in rural facilities: a cluster randomized trial. Nat Med. 2026 Feb 17; doi:10.1038/s41591-026-04222-y](https://ad-id.co/4sayZKA) | **P:** 97,239 acute respiratory infection (ARI) consultations in 34 township hospitals (cluster RCT) in rural Guangdong, China  **I:** Digitally enabled antimicrobial stewardship program (doctor training and guidelines, EMR-embedded decision prompts, monthly peer-review feedback, patient education via smartphone app)  **C:** Usual care (no stewardship inputs)  **O:** Antibiotic prescribing at consultation; 30-day hospitalization for respiratory illness or sepsis | Antibiotics were prescribed in 26% (14,521/54,799) of intervention consultations vs 71% (30,340/42,440) in controls, corresponding to an adjusted risk difference of –39 percentage points (95% CI –47 to –29; p<0.001). There was no evidence of increased harm, with similar 30-day hospitalization rates for respiratory illness or sepsis (adjusted risk difference 0.2 percentage points; 95% CI –0.3 to 0.6). The intervention substantially reduced antibiotic prescribing without compromising short-term safety. | Moderate risk: The cluster-randomized pragmatic design strengthens real-world applicability but may introduce cluster-level confounding despite adjustment. Lack of blinding could influence prescribing behavior (performance bias). The large sample size and objective prescribing outcomes enhance reliability. Potential contamination between clusters cannot be excluded. Overall internal validity is reasonable with strong effect size but some risk of behavioral bias. |
| [Huang WC, Huang YW, Lin CB, Chien ST, Lee CH, Yu MC, et al. Xpert MTB/RIF assay as an initial diagnostic test in pulmonary tuberculosis: a multicenter prospective randomized pragmatic trial. Sci Rep. 2026 Feb 21; doi:10.1038/s41598-026-39022-4](https://ad-id.co/4saz016) | **P:** 6,835 patients evaluated for pulmonary tuberculosis (TB) in Taiwan; patients without clinician-requested NAAT randomized 1:1 to intervention or usual care  **I:** Immediate Xpert MTB/RIF assay as initial diagnostic test (group B)  **C:** Usual diagnostic care without immediate Xpert (group C)  **O:** Proportion diagnosed with active TB; death before anti-TB treatment; time to treatment initiation; treatment success | Comparing immediate Xpert vs usual care, there were no significant differences in active TB diagnosis (3.1% vs 2.7%, p=0.336), death before treatment (2.3% vs 5.1%, p=0.318), median time to treatment initiation (7.0 vs 6.0 days, p=0.589), or treatment success among TB cases (73.8% vs 81.8%, p=0.657). Expanded upfront Xpert testing in a population with relatively low pretest probability did not improve TB control outcomes. | Moderate risk: Randomization between intervention and usual care reduces selection bias, but lack of reported blinding may introduce performance bias. Outcomes such as diagnosis and mortality are objective, limiting detection bias. The low prevalence setting may reduce power to detect differences in clinically important outcomes. Inclusion of a non-randomized clinician-selected group (group A) may complicate interpretation, though primary comparison was randomized. Overall internal validity is moderate. |
| [Pieren M, Dale GE, Gitzinger M, Tiberi S, Penman SL, Remuiñán MJ, et al. Revival of Ethionamide by Alpibectir. N Engl J Med. 2026 Feb 19;394(8):818-819.](https://ad-id.co/3OHBG8k) | **P:** 105 adults with newly diagnosed rifampin-susceptible pulmonary tuberculosis (14–16 participants per group)  **I:** Alpibectir plus ethionamide combinations (9 mg + 250 mg; 27 mg + 125 mg; 27 mg + 250 mg; 27 mg + 500 mg) for 7 days  **C:** Isoniazid 300 mg daily or ethionamide 250 mg or 750 mg daily  **O:** Early bactericidal activity (change in sputum colony-forming units over 7 days); adverse events | Ethionamide 750 mg showed bactericidal activity similar to isoniazid 300 mg. Ethionamide demonstrated dose-related activity, which was potentiated by increasing doses of alpibectir. The 27-mg alpibectir plus 250-mg ethionamide regimen achieved bactericidal activity comparable to 750-mg ethionamide and isoniazid, with mostly mild-to-moderate gastrointestinal adverse events and a lower side-effect profile than higher-dose ethionamide. Proof of concept was established for alpibectir-enhanced ethionamide activity. | Moderate to high risk: Small sample size with 14–16 participants per arm limits statistical power and precision. Short 7-day follow-up assesses early bactericidal activity rather than clinical outcomes. Details on blinding are not specified, potentially introducing performance or detection bias. Objective microbiological endpoints reduce measurement bias, but the early-phase design limits generalizability and long-term safety conclusions. |
| [Tavoulareas G, Kontakou-Zoniou O, Antonakos N, Tasouli E, Adamis G, Kakavouli N, et al. Efficacy of anakinra in reducing progression to organ dysfunction in patients with pneumonia (INSPIRE): a randomised, double-blind, placebo-controlled, phase IIa trial. Lancet Reg Health Eur. 2026 Jan 7;62:101573; doi:10.1016/j.lanepe.2025.101573](https://ad-id.co/4saz0hC) | **P:** 60 hospitalized adults with community-acquired or hospital-acquired pneumonia, qSOFA = 1, plasma presepsin >350 pg/mL  **I:** Standard-of-care plus subcutaneous Anakinra 100 mg once daily for 10 days (n=30)  **C:** Standard-of-care plus subcutaneous placebo for 10 days (n=30)  **O:** Progression to organ dysfunction by day 7 (≥2-point SOFA increase) and/or death by day 90; 90-day mortality; cytokine production | The primary endpoint occurred in 20% of Anakinra-treated patients vs 50% in placebo (difference 30%; 95% CI 5.9–49%; p=0.011). 90-day mortality was 20% vs 43.3% (difference 23.3%; 95% CI 0–43.7%; p=0.029). Serious treatment-emergent adverse events occurred in 33.3% vs 50%, none judged treatment-related. TNFα and IFNγ production by blood mononuclear cells was decreased, indicating cytokine attenuation as a potential mechanism. | Moderate risk: Double-blind, randomized, placebo-controlled design reduces selection and detection bias. Small sample size (30 per arm) limits precision and generalizability. Short follow-up for organ dysfunction assessment may miss late events. Biomarker-based inclusion improves mechanistic insight but may reduce external validity. Overall, internal validity is reasonable for a proof-of-concept trial. |
| [Hossain MJ, Secka F, Sanyang LC, Taiwo R, Okoh EC, Olubiyi OA, et al. Efficacy of ETVAX, a vaccine against enterotoxigenic Escherichia coli-positive diarrhoea in Gambian children: a double-blind, randomised, placebo-controlled, phase 2b trial. Lancet Infect Dis. 2026 Feb 16; doi:10.1016/S1473-3099(25)00774-1](https://ad-id.co/404gU5c) | **P:** 4,936 Gambian children aged 6–18 months (2,468 ETVAX, 2,468 placebo)  **I:** Three-dose oral ETVAX vaccine on days 1, 15, and 90  **C:** Placebo  **O:** Primary: vaccine efficacy against moderate-to-severe ETEC-positive diarrhoea (MSD-ETEC) excluding major viral/bacterial copathogens; Secondary: efficacy against MSD-ETEC regardless of copathogens and all-cause moderate-to-severe diarrhoea (MSD); Safety: serious adverse events | ETVAX was safe with serious adverse events in 1.0% vs 1.3% of placebo, none vaccine-related. Immunogenicity subset (n=122) showed increased antibodies to colonisation factors CFA/I, CS3, and heat-labile toxins. Vaccine efficacy was 26.6% (95% CI −58.3 to 66.0; p=0.43) for the primary endpoint, 48.2% (p=0.053) against MSD-ETEC regardless of copathogens, and 80.6% (p=0.0092) when excluding enteroparasitic copathogens. Efficacy reached 67.8% (p=0.026) when first dose given before age 9 months. All-cause MSD was reduced by 21.4% (p=0.032). | Low risk: Double-blind, placebo-controlled, randomized design with block randomization and stratification by site reduces selection and performance bias. Large sample size enhances precision. Masking of participants, parents, staff, and outcome assessors minimizes detection bias. Analysis followed per-protocol and intention-to-treat principles. Some exploratory subgroup analyses may be underpowered. Overall internal validity is high. |
| [Baghdadi JD, Harris AD, Pineles L, Al-Shanqeeti S, Palacio D, Charles DW, et al. Using probability of community-acquired pneumonia to tailor antimicrobials among inpatients: a pragmatic, randomized trial. Clin Infect Dis. 2026 Mar 13; doi:10.1093/cid/ciag126](https://ad-id.co/4rBB6qm) | **P:** 107 hospitalized adults receiving antibiotics for suspected respiratory infection with either low procalcitonin (65%), positive respiratory virus testing (30%), or both (5%) at 2 hospitals.  **I:** Antimicrobial stewardship–guided interpretation of test results via a templated electronic health record note providing post-test probability of bacterial pneumonia and antibiotic decision recommendations.  **C:** Usual care without stewardship-guided interpretation.  **O:** In-hospital antibiotic days of therapy; early discontinuation of respiratory antibiotics; length of stay; 30-day readmission. | Stewardship-guided interpretation significantly reduced antibiotic exposure, with mean in-hospital antibiotic days of therapy of 7.5 vs 11.6 in usual care (difference −4.1 days, P = .006). Respiratory antibiotics were discontinued within 5 days for 76% of intervention patients vs 49% in the usual care group (P = .004). There were no statistically significant differences in hospital length of stay (5.5 vs 6.6 days, P = .16) or 30-day readmission (7% vs 19%, P = .079), suggesting reduced antibiotic use without evidence of worse clinical outcomes. | Moderate risk: The pragmatic design enhances real-world applicability but the relatively small sample size and proof-of-concept nature limit statistical power and generalizability. Lack of blinding could introduce performance bias in prescribing behavior. However, randomized allocation and objective antibiotic utilization outcomes strengthen internal validity. |
| [Winthrop KL, Flume PA, Khare R, Sriaroon C, Sirbu A, Manley A, et al. Omadacycline monotherapy in nontuberculous mycobacterial pulmonary disease caused by Mycobacterium abscessus: results from a phase 2, double-blind, randomized, placebo-controlled study. Clin Infect Dis. 2026 Mar 3; doi:10.1093/cid/ciag062](https://ad-id.co/4bcG485)   - **Editorial Commentary:**   [Marino A, Marras TK. A framework for progress: what a phase 2 trial teaches us about Mycobacterium abscessus research. Clin Infect Dis. 2026 Mar 3; doi:10.1093/cid/ciag063](https://ad-id.co/4rtPUaf) | **P:** 66 adults with nontuberculous mycobacterial pulmonary disease caused by Mycobacterium abscessus meeting diagnostic criteria (randomized 1.5:1; prior antibiotic treatment stratified).  **I:** Omadacycline 300 mg orally once daily for 84 days as monotherapy.  **C:** Placebo for 84 days.  **O:** Symptom response at day 84 (improvement in severity of ≥50% of baseline symptoms; and composite response including no worsening of any baseline symptom); secondary clinical and microbiological outcomes; adverse events. | Among 66 randomized patients (41 omadacycline, 25 placebo), symptom response at day 84 favored omadacycline: 34.1% vs 20.0% for responder definition 1 (improvement in ≥50% of baseline symptoms) and 34.1% vs 12.0% for responder definition 2 (definition 1 plus no worsening of any baseline symptom). Secondary and exploratory clinical and microbiological outcomes also favored omadacycline. Treatment-emergent adverse events leading to discontinuation occurred in 9.8% of omadacycline patients, with gastrointestinal effects—particularly nausea—the most common drug-related adverse events. | Moderate risk: The randomized, double-blind, placebo-controlled design strengthens internal validity. However, the small phase 2 sample size and short 84-day follow-up limit statistical power and long-term outcome assessment. Reliance on symptom-based primary endpoints may introduce subjectivity despite blinding, and results may have limited generalizability beyond the studied population. |
| [Yahav D, Pinto R, Cook D, Davis J, Duan E, Hoffman T, et al. Duration of therapy for Pseudomonas aeruginosa bacteremia – a post hoc subgroup analysis from the BALANCE randomized controlled trial. Clin Infect Dis. 2026 Mar 3; doi:10.1093/cid/ciag144](https://ad-id.co/3NGAEcl)   - **Editorial Commentary:**   [Hojat LS, Spivak ES. Another point goes to Team Short Course: implications of the BALANCE trial post-hoc analysis for Pseudomonas aeruginosa bloodstream infections. Clin Infect Dis. 2026 Mar 3; doi:10.1093/cid/ciag145](https://ad-id.co/3NGAEsR) | **P:** 157 hospitalized patients with *Pseudomonas aeruginosa* bacteremia included in the BALANCE randomized controlled trial.  **I:** 7 days of antibiotic therapy for bacteremia.  **C:** 14 days of antibiotic therapy for bacteremia.  **O:** 90-day mortality. | Among 157 patients (74 in the 7-day group and 83 in the 14-day group), crude 90-day mortality was 29.7% with 7 days vs 20.5% with 14 days of therapy (risk difference 9.2%, 95% CI −4.8 to 23.2), showing no statistically significant difference between groups. In multivariable analysis, increasing age was independently associated with higher 90-day mortality (OR 1.06 per year, 95% CI 1.02–1.09), whereas treatment duration (7 vs 14 days) was not associated with mortality (OR 1.47, 95% CI 0.66–3.27). | Moderate risk: This was a post hoc subgroup analysis of an RCT rather than a trial specifically powered for *P. aeruginosa* bacteremia, increasing risk of imprecision and potential imbalance between groups. The relatively small sample size limits statistical power and prevents firm conclusions about noninferiority. However, randomization in the parent trial and objective mortality outcomes reduce risk of major bias. |
| [Athan E, Greenberg RN, Baker DA, Shah R, Dubhashi S, Badat A, et al. Safety, efficacy, and immunogenicity of a multivalent adjuvanted S. aureus vaccine in adults with recent skin and soft tissue infections: an observer-blind, randomized, placebo-controlled, multinational phase 1/2 trial. Clin Infect Dis. 2026 Mar 9; doi:10.1093/cid/ciag162](https://ad-id.co/4lAxHH2) | **P:** 194 participants aged 18–64 years with a recent history of *Staphylococcus aureus* skin and soft tissue infections (SA-SSTIs).  **I:** Two full doses of AS01E-adjuvanted five-antigen S. aureus vaccine (SA5Ag-Adj), 2 months apart.  **C:** Placebo, 2 months apart.  **O:** Vaccine efficacy in preventing recurrent SA-SSTIs over 12 months; safety; immunogenicity. | At 12 months post-second dose, SA5Ag-Adj showed no efficacy in preventing recurrent SA-SSTIs (vaccine efficacy −38.1%, 95% CI −245.8 to 40.9) despite robust functional immune responses against three of the five vaccine antigens (CP5, CP8, Hla). Solicited local adverse events were more frequent with SA5Ag-Adj but mostly mild or moderate; medically attended and serious adverse events were similar between groups. | Moderate risk: Randomized, placebo-controlled design supports internal validity, but early termination for futility and modest sample size limit statistical power and precision of efficacy estimates. Safety outcomes are reliable due to objective reporting, but results may not generalize beyond the studied adult population with recent SA-SSTI. |
| [Durovni B, Cordeiro-Santos M, Cavalcante SC, Spener-Gomes R, Garcia J, Cohn S, et al. Acceptability and safety of one versus three months of rifapentine and isoniazid to prevent tuberculosis in people exposed in the household or workplace in Brazil: the Ultra-Curto randomized controlled trial. PLoS Med. 2026 Feb 10; doi:10.1371/journal.pmed.1004758](https://ad-id.co/4lusssj) | **P:** 500 adolescents and adults without HIV infection with recent tuberculosis exposure and a positive latent tuberculosis infection test (249 assigned to 1HP, 251 to 3HP; 193 males, 307 females; median age 39 years).  **I:** One month of daily isoniazid and rifapentine (1HP).  **C:** Three months of weekly isoniazid and rifapentine (3HP).  **O:** Treatment completion (>90% of doses) and safety (Grade >2 adverse events or treatment discontinuation). | Treatment completion was high for both regimens: 89.6% for 1HP versus 84.1% for 3HP (site-adjusted risk difference 5.2%, 95% CI −0.1 to 11.2%, p = 0.10). Targeted >Grade 2 adverse events or treatment discontinuation occurred in 16.1% of 1HP versus 10.4% of 3HP recipients (site-adjusted risk difference 6.1%, 95% CI −0.04 to 12.3%, p = 0.05), with most events being low-grade. Discontinuation for any side effect was 7.2% for 1HP and 4.4% for 3HP. Overall, neither regimen demonstrated clear superiority in completion or safety. | Low-moderate risk: Randomized phase 4 design with objective completion measures supports internal validity. Open-label administration may have introduced reporting bias for adverse events, but high adherence assessment and large sample size strengthen reliability. Results may not generalize to people living with HIV or other populations. |
| [Hansen JR, Hillerup S, Greibe E, Gade S, Pikelis A, Hanberg P, et al. Cefuroxime concentrations in facial artery musculomucosal flap, buccal submucosa, subcutaneous tissue, and plasma following bolus or continuous infusion in patients undergoing oral cancer resection: a randomized clinical microdialysis study. J Antimicrob Chemother. 2026 Apr; doi:10.1093/jac/dkag085](https://ad-id.co/4bcub1W) | **P:** 18 patients scheduled for tumor resection and facial artery musculomucosal (FAMM) flap reconstruction for oral cavity cancer.  **I:** Continuous infusion (CI) of cefuroxime 4500 mg/day.  **C:** Bolus infusion (BI) of cefuroxime 1500 mg every 8 hours.  **O:** Free cefuroxime concentrations in plasma, FAMM flap, buccal submucosa, and subcutaneous tissue; time above minimal inhibitory concentration (T > MIC) and attainment of 50%T > MIC for MIC 2 and 4 mg/L. | All patients achieved ≥50%T > MIC in all tissues for both MIC targets. CI achieved 100%T > MIC across all compartments and MIC thresholds, whereas BI achieved 89%–98% for MIC 2 mg/L and 79%–90% for MIC 4 mg/L. CI showed significantly higher T > MIC than BI in subcutaneous tissue for both MIC thresholds and in plasma for MIC 4 mg/L, indicating more consistent exposure. | Moderate risk: Small sample size limits statistical power and generalizability. Randomized allocation reduces selection bias, but unblinded pharmacokinetic measurements could introduce detection bias. Short observation period (8 hours) may not capture longer-term variability in drug exposure. |
| [Neyra JA, Legrand M, Tidswell MA, Al-Khafaji A, Galphin C, Rains R, et al. Polymyxin B haemoadsorption in endotoxic septic shock (Tigris): a multicentre, open-label, Bayesian, randomised, controlled, phase 3 trial. Lancet Respir Med. 2026 Mar 23; doi:10.1016/S2213-2600(26)00047-0](https://ad-id.co/4bMWm6o)  **Editorial Commentary:** [Schupp T, Behnes M, Akin I. Implementing endotoxin adsorption for septic shock into clinical practice. Lancet Respir Med. 2026 Mar 23; doi:10.1016/S2213-2600(26)00055-X](https://ad-id.co/4dg8rnf) | **P:** 157 adults with endotoxic septic shock requiring vasopressors, multiorgan failure, and endotoxin activity between 0.60 and 0.89 units.  **I:** Two 90-120 minute sessions of polymyxin B haemoadsorption, administered 22 hours apart, in addition to standard of care.  **C:** Standard of care alone.  **O:** The primary outcome was 28-day mortality. The key secondary outcome was 90-day mortality. | Polymyxin B haemoadsorption was associated with a high probability of benefit for mortality. At 28 days, mortality was 39% in the polymyxin B group versus 45% in the control group (adjusted odds ratio 0.67, 95% credible interval 0.39–1.08), yielding a 95.3% posterior probability of benefit. The benefit was more pronounced at 90 days, with a 99.4% posterior probability of a lower mortality rate (adjusted odds ratio 0.54, 95% credible interval 0.32–0.87). Serious adverse events were more common in the treatment group (30% vs. 22%), but only two events were deemed related to the treatment. | Moderate risk: The open-label design is a significant limitation, creating a high risk of performance and detection bias, as clinicians and patients were aware of the treatment allocation. The study's credibility is strengthened by its randomized design, use of an intention-to-treat analysis, and hard mortality endpoints. However, the use of a Bayesian framework that borrowed from a prior trial (EUPHRATES) and the relatively small sample size are potential limitations that could influence the results. |
| [Lotz C, Heckelmann J, Lendzian C, Herrmann J, Haack B, Gieselmann M, et al. Clinical and Immunologic Effects of Extracorporeal Cytokine Removal in Patients with Septic Shock: A Randomized Controlled Trial. Shock. 2026 Apr;65(4):637-47. doi:10.1097/SHK.0000000000002802](https://ad-id.co/4dg8rDL) | **P:** 31 adult patients with septic shock, an existing extracorporeal circuit (CVVHD or ECMO), and hyperinflammation (interleukin-6 >500 pg/mL).  **I:** Standard care plus CytoSorb hemoadsorption, initiated within 24 hours of septic shock onset.  **C:** Standard care alone, according to international sepsis guidelines.  **O:** Primary: Cumulative norepinephrine dose over 72 hours. Secondary: Survival at 48/72 hours, ICU mortality, length of stay, duration of septic shock, and various immunological endpoints. | Early hemoadsorption with CytoSorb did not improve outcomes and was associated with worse results on some measures. The primary outcome, cumulative norepinephrine dose at 72 hours, was not significantly different between the intervention and control groups (100.7 mg vs 78 mg, P=0.09). However, the control group had a significantly lower total vasopressor dose per hour alive (1.2 mg vs. 2.5 mg, P=0.0053) and significantly higher survival rates at both 48 hours (100% vs. 64%, P=0.01) and 72 hours (94% vs. 57%, P=0.03). No significant differences were observed in ICU mortality, length of stay, or humoral immune responses. | High risk: The open-label design, where patients, care providers, and the study team were all unblinded, creates a significant potential for performance and detection bias. The very small sample size (n=31) and single-center design severely limit the statistical power and the generalizability of the findings. Furthermore, the duration of hemoadsorption and the timing of adsorber changes were left to the discretion of the attending ICU team, introducing potential variability and inconsistency in the application of the intervention. |
| [Chen WH, Beckett CG, Al-Ibrahim M, Datar R, Sikorski MJ, Liang Y, et al. Safety and Immunogenicity of a Trivalent Salmonella Conjugate Vaccine to S. Typhi, S. Typhimurium, and S. Enteritidis. J Infect Dis. 2026 Mar 26; doi:10.1093/infdis/jiag156](https://ad-id.co/4s7pLyx) | **P:** 80 healthy adults aged 20–47 years.  **I:** A single dose of one of three formulations of a trivalent Salmonella conjugate vaccine (TSCV): full-strength (FS), half-strength (HS), or dilutional half-strength (dilHS).  **C:** Placebo.  **O:** Safety (local and systemic adverse events) and immunogenicity (serum antibody and antibody-secreting cell responses). | All three vaccine formulations were well tolerated, with mild injection site pain and fatigue being the most common adverse events. The vaccines produced robust immune responses to all three Salmonella antigens, with response rates ranging from 85% to 100%. There was no statistically significant difference in the immune response observed between the three different vaccine strengths. As expected, participants who received the placebo did not show any significant antibody or cellular responses. | Low to Moderate risk: As a Phase 1/2a trial, the study has a small sample size (n=80), which limits statistical power and the generalizability of the findings to the target population of young children. The abstract does not specify if the trial was blinded, which could introduce performance or detection bias if it was an open-label study. The use of healthy adults as a surrogate for the pediatric target population is a standard practice for initial safety trials but means the immunogenicity results may not be directly transferable. |
| [Todd S, Euden J, Condie J, Aston S, Barlow G, Brookes-Howell L, et al. Procalcitonin testing combined with NEWS2 evaluation compared with usual care based on NEWS2 for identification of sepsis and antibiotic initiation in the emergency department in England and Wales (PRONTO): a multicentre, randomised, controlled, open-label, phase 3 trial. Lancet Respir Med. 2026 Mar 22; doi:10.1016/S2213-2600(25)00433-3](https://ad-id.co/4bXqkVx)  **Editorial Commentary:** [Cabral S, Rhee C. Rethinking the role of procalcitonin in suspected sepsis. Lancet Respir Med. 2026 Mar 22; doi:10.1016/S2213-2600(25)00471-0](https://ad-id.co/4dg8saN) | **P:** 5453 adults (from an initial 7667 randomized) with suspected sepsis presenting to 20 UK emergency departments.  **I:** Procalcitonin-guided care, using rapid procalcitonin testing in combination with standard NEWS2 assessment and a guidance algorithm.  **C:** Usual care, based on standard clinical management with NEWS2 assessment.  **O:** Co-primary endpoints were intravenous antibiotic initiation at 3 hours (for superiority) and 28-day mortality (for non-inferiority). | The availability of a procalcitonin-guided algorithm did not reduce early antibiotic use; there was no significant difference in intravenous antibiotic initiation at 3 hours between the groups (48.4% in the procalcitonin group vs. 48.2% in the usual care group). However, the study found an unexpected reduction in 28-day mortality in the procalcitonin group (13.6%) compared to the usual care group (16.6%), a result that met the criteria for both non-inferiority and superiority. The reason for this mortality benefit was not explained by the study's analyses. | High risk: The open-label design, where participants, clinicians, and research staff were all unmasked to the treatment allocation, creates a significant risk of performance and detection bias. This knowledge could have influenced clinical behaviors in ways other than just following the algorithm, potentially confounding the results. Adherence to the intervention was also suboptimal, as clinicians only considered the procalcitonin result in about 65% of cases, which complicates the interpretation of the findings. |
| [Wrønding T, Vomstein K, Lundgaard AT, DeLong K, Mollerup S, Mortensen B, et al. Vaginal microbiota transplantation for treatment of vaginal dysbiosis without the use of antibiotics: a double-blind, randomised controlled trial in women with vaginal dysbiosis. Lancet Microbe. 2026 Mar 26; doi:10.1016/j.lanmic.2025.101294](https://ad-id.co/4s8znce) | **P:** 49 premenopausal women (aged 18–40) with asymptomatic or symptomatic molecular vaginal dysbiosis (defined as <10% Lactobacillus spp and >20% combined Gardnerella spp, Fannyhessea vaginae, and Prevotella spp).  **I:** Up to three administrations of vaginal microbiota transplant (VMT) across three menstrual cycles, without antibiotic pretreatment.  **C:** Placebo administration.  **O:** The primary outcome was the resolution of dysbiosis (defined as ≥70% Lactobacillus spp and <10% combined dysbiotic bacteria) at any timepoint during a six-cycle follow-up. | The study found no significant difference in the resolution of dysbiosis between the VMT and placebo groups (Hazard Ratio 0.65, 95% CI 0.20–2.16; p=0.49). Adverse events were equally common in both groups (42%), with no serious events reported. However, in a small, un-randomized extension study involving 10 participants who had not responded to the initial treatment, 50% achieved microbiome conversion after receiving an antiseptic pretreatment followed by another VMT. | Moderate risk: The study's main strength is its double-blind, randomized, placebo-controlled design, which minimizes performance and detection bias. However, the very small sample size (n=49) is a significant limitation, as it provides low statistical power to detect a true difference between the groups, increasing the risk of a false-negative result (Type II error). The single-center design may also limit the generalizability of the findings. |
| [Lee JL, Kim HJ, Kim KM, Yi JM, Oh J, Lee EK, et al. Target-controlled infusion vs standard dosing of cefoxitin for surgical prophylaxis in colorectal surgery: A randomized clinical trial. Clin Microbiol Infect. 2026 Mar 17; doi:10.1016/j.cmi.2026.03.019](https://ad-id.co/4s3VuAA) | **P:** 2,494 adults undergoing elective colorectal surgery.  **I:** Cefoxitin administered via a target-controlled infusion (TCI) pump targeting a specific plasma concentration (80 μg/mL).  **C:** Standard dosing of cefoxitin (2 g every 2 hours).  **O:** Primary: Incidence of surgical site infection (SSI) within 30 days. Secondary: Intraoperative cumulative cefoxitin dose and incidence of acute kidney injury (AKI). | The study found no difference in the primary outcome, with the incidence of surgical site infection being identical in both groups (5.6%). However, the target-controlled infusion (TCI) method resulted in a significantly lower intraoperative antibiotic exposure, with the median cumulative dose being approximately 30% less than in the standard dosing group (1.38 g vs. 2.00 g). There was no significant difference in the rates of acute kidney injury between the two groups. | Low to Moderate risk: The study's major strength is its large sample size (n=2,494) and randomized controlled design, which provides high statistical power for the primary outcome. The endpoints are objective, reducing the risk of detection bias. However, the abstract does not specify whether surgeons or outcome assessors were blinded to the treatment allocation, which could introduce bias. Additionally, as a single-center trial, the findings may have limited generalizability to other institutions with different patient populations or surgical protocols. |
| [Bijukchhe SM, Marchevsky NG, Kibengo F, Sharma AK, Basi R, Cantrell L, et al. Optimising DTwP-containing vaccine infant immunisation schedules in Uganda and Nepal (OptImms): two open-label, non-inferiority, randomised controlled trials. Lancet Infect Dis. 2026 Mar 19; doi:10.1016/S1473-3099(26)00053-8](https://ad-id.co/4dg8sYl) | **P:** 1727 healthy infants (876 in Uganda, 851 in Nepal) aged 42–50 days at enrollment.  **I:** Four alternative DTwP-Hib-HepB vaccination schedules: two reduced-dose schedules (at 6 & 14 weeks; or 2 & 4 months) and two delayed three-dose schedules (at 2, 3, & 4 months; or 2, 4, & 6 months).  **C:** The standard three-dose WHO schedule (at 6, 10, and 14 weeks).  **O:** The primary outcome was the pre-booster IgG antibody response against pertussis antigens to assess if the two-dose schedules were non-inferior to the WHO schedule. Secondary outcomes included antibody responses at other time points. | The reduced two-dose schedules failed to meet the non-inferiority criteria for pre-booster pertussis antibody levels compared to the standard WHO schedule. While the delayed three-dose schedules produced similar or higher antibody levels just before the booster, the standard WHO schedule was superior in generating higher antibody responses during the first 3 months of life. The authors concluded that the standard WHO schedule is preferable, especially in high-pertussis-burden settings, as it provides the best protection when infants are most vulnerable. | Moderate risk: The open-label design, where investigators and participants were not blinded to the vaccine schedule, could introduce performance bias. However, this risk is mitigated because the primary outcome is an objective laboratory measurement (antibody levels), which is not susceptible to subjective interpretation. The study's strengths include its large, multi-country, randomized design and the use of a pre-defined non-inferiority margin, which strengthens the validity of the primary analysis. |
| [Wassil J, Fairman J, Fierro CA, Clark J, Bennett S, Johnson D, et al. Safety, tolerability, and immunogenicity of a 31-valent pneumococcal conjugate vaccine (VAX-31) in healthy adults aged 50 years and older from the USA: a phase 1/2, double-blinded, active-controlled, parallel-group, dose-finding randomised clinical trial. Lancet Infect Dis. 2026 Mar 18; doi:10.1016/S1473-3099(26)00059-9](https://ad-id.co/4dg8teR) | **P:** 1015 healthy, pneumococcal-naive adults aged 50 years or older.  **I:** A single intramuscular dose of VAX-31, a 31-valent pneumococcal conjugate vaccine (PCV), at one of three dose levels (low, mid, or high).  **C:** A single intramuscular dose of the licensed 20-valent PCV (PCV20).  **O:** Primary outcomes were safety and tolerability (adverse events). Secondary outcomes were immunogenicity, measured by serotype-specific opsonophagocytic activity (OPA) and IgG concentrations at 1 month. | VAX-31 was well tolerated at all doses, with a safety profile comparable to the licensed PCV20. Most adverse events were mild to moderate. The mid-dose and high-dose VAX-31 formulations demonstrated immune responses that were non-inferior to PCV20 for all 20 shared serotypes. Furthermore, all three doses of VAX-31 elicited superior immune responses for the 11 additional serotypes not contained in PCV20. | Low risk: The study's double-blind, randomized, active-controlled design is a major strength, minimizing the risk of performance and detection bias. The use of a multi-center approach enhances the generalizability of the findings. As a Phase 1/2 trial, its primary focus is on safety and immunogenicity rather than clinical efficacy, which is an inherent limitation of early-phase research but not a flaw in its design for the stated objectives. |
| [Last A, Abdurahman OS, Greenland K, Robinson A, Etu ES, Butcher R, et al. Double-dose azithromycin mass drug administration, facial cleanliness, and fly control measures for trachoma control in Oromia, Ethiopia (Stronger SAFE): a cluster-randomised controlled trial. Lancet Infect Dis. 2026 Mar 24; doi:10.1016/S1473-3099(26)00024-1](https://ad-id.co/418TrAe) | **P:** 68 rural community clusters in Oromia, Ethiopia, with the primary outcome assessed in a cross-section of 3,480 children aged 1–9 years.  **I:** The "Stronger SAFE" intervention, consisting of enhanced antibiotics (two annual doses of azithromycin 2 weeks apart) plus enhanced facial cleanliness and environmental improvement (fly control and a household behavior change intervention).  **C:** The "Standard SAFE" intervention (control group), consisting of annual single-dose azithromycin mass drug administration plus standard promotion of latrine use and facial hygiene.  **O:** The primary outcome was the prevalence of conjunctival *Chlamydia trachomatis* infection at 3 years. | After three years of intervention, both groups saw a substantial reduction in the prevalence of *C. trachomatis* infection. However, the enhanced "Stronger SAFE" strategy provided no additional benefit compared to the standard SAFE strategy. The infection prevalence was 2.2% in the Stronger SAFE group versus 2.7% in the standard care group, a difference that was not statistically significant. The authors concluded that these enhanced measures are unlikely to accelerate trachoma elimination beyond what can be achieved with well-implemented, high-coverage standard SAFE. | Moderate risk: The study has a strong cluster-randomized design with an objective primary outcome (PCR test), and key personnel (lab technicians, statistician) were masked, which reduces detection and analysis bias. However, the open-label nature of the trial, where communities and field staff knew the intervention allocation, creates a risk of performance bias. This could have led to a "study effect," where the standard SAFE intervention in the control group was implemented with unusually high fidelity and coverage, potentially masking any true additional benefit from the enhanced interventions. |
| [de Graaf H, Gbesemete DF, Hill AR, Fröberg J, Ibrahim MM, Dale AP, et al. Safety, colonisation kinetics, transmissibility, and immune correlates of protection in healthy adults inoculated with Bordetella pertussis in England: a single-centre, open-label, phase 1, controlled human infection study. Lancet Microbe. 2026 Mar 23; doi:10.1016/j.lanmic.2025.101313](https://ad-id.co/4c55UKu) | **P:** 51 healthy volunteers aged 18–55 years who had previously received a whole-cell pertussis vaccine in childhood.  **I:** Intranasal inoculation with wild-type *Bordetella pertussis* in an outpatient setting, with a subsequent re-inoculation for a subset of participants.  **C:** The primary comparison was between volunteers who became colonized with *B. pertussis* and those who did not, to identify immunological correlates of protection.  **O:** The primary outcome was safety. Secondary outcomes included the rate of bacterial colonization, identification of immunological biomarkers associated with protection, and assessment of transmission to close contacts. | The outpatient controlled human infection model was found to be safe, with adverse events being mostly mild to moderate and no serious adverse events related to the inoculation. 40% of volunteers became colonized. Protection from colonization was associated with higher pre-existing antibody levels (including IgG and IgA) and specific T-cell responses. Infection-induced immunity was strong, as only 1 of 13 previously colonized volunteers became re-colonized upon rechallenge. No transmission was detected to 14 enrolled close contacts. | Low to Moderate risk: The study's open-label design is inherent to a human challenge model but introduces a risk of performance and detection bias for subjective outcomes like self-reported symptoms. However, the key outcomes of colonization and immunological response were measured by objective laboratory tests, minimizing this bias. The main limitations are the small, single-center sample size and the specific population (healthy adults primed with whole-cell vaccine), which restrict the generalizability of the findings to other populations, such as those primed with acellular vaccines. |
| [Singh M, Joshi S, Vohra V, Sarin R, Kamble SV, Velayutham B, et al. Efficacy and safety of VPM1002 and Immuvac in preventing tuberculosis: phase 3 randomised clinical trial (PreVenTB trial). BMJ. 2026 Apr 9;393:e085716. doi:10.1136/bmj-2025-085716](https://ad-id.co/421xa7Q) | **P:** 12,717 healthy household contacts (aged ≥6 years) of patients with smear-positive TB across India  **I:** Intradermal VPM1002 vaccine (with second dose in one arm after 1 month)  **C:** Placebo (and Immuvac as an additional comparator arm)  **O:** Incidence of microbiologically confirmed TB (pulmonary and extrapulmonary) over 38 months; latent TB infection; safety; immunogenicity | VPM1002 did not significantly reduce overall TB incidence compared with placebo (1.68% vs 2.13%; vaccine efficacy 21.4%, 95% CI −8.9% to 43.2%) or pulmonary TB (19.5%, 95% CI −14.6% to 43.4%), but showed significant efficacy against extrapulmonary TB (50.4%, 95% CI 0.8% to 75.2%). Immuvac did not reduce overall TB incidence but demonstrated efficacy against extrapulmonary TB (33.2%, 95% CI −25.9% to 64.5%). Both vaccines showed higher efficacy against extrapulmonary TB among tuberculin skin test–positive participants (VPM1002 64.9%, Immuvac 66.3%). Post hoc analysis suggested greater efficacy of VPM1002 in participants aged 6–14 years for all TB (64.6%, 95% CI 16.3% to 85.1%). Both vaccines were well tolerated with mainly mild local reactions and induced Mycobacterium tuberculosis–specific polyfunctional CD4+ T cell responses. | Moderate risk: The randomized design and large sample size strengthen internal validity, but potential performance and detection bias may exist due to limited detail on blinding. The modest effect sizes with wide confidence intervals (crossing null for primary outcomes) suggest limited statistical power for some endpoints. Post hoc subgroup analyses increase risk of selective reporting bias. High follow-up and objective microbiological outcomes reduce attrition and measurement bias. |
| [Louie T, Ribble W, Boccumini L, Johnson K, De Groote MA, Day J, et al. Safety and efficacy of CRS3123 in adults with a primary episode or first recurrence of Clostridioides difficile infection: a phase 2, randomised, double-blind, multicentre, vancomycin-controlled study. Lancet Infect Dis. 2026 Jan 22; doi:10.1016/S1473-3099(25)00721-2](https://ad-id.co/4stt8Qj) | **P:** 43 adults (mean age 58.4 years, 77% female; 72% primary CDI, 28% first recurrence) with Clostridioides difficile infection  **I:** CRS3123 (200 mg or 400 mg orally twice daily for 10 days)  **C:** Oral vancomycin (125 mg four times daily for 10 days)  **O:** Clinical cure at test-of-cure (day 12–15); CDI recurrence at days 40 and 70; safety (adverse events) | Clinical cure rates at test-of-cure were similar across groups (CRS3123 200 mg: 93%; CRS3123 400 mg: 100%; vancomycin: 93%). Recurrence by day 40 was lower with CRS3123 (0% for 200 mg; 7% for 400 mg) compared with vancomycin (23%), with one additional recurrence at day 70 in the CRS3123 400 mg group. No clinical failures were observed; two patients had indeterminate outcomes due to missing data. Treatment-emergent adverse events were mild to moderate and comparable across groups, with no serious adverse events attributed to CRS3123. | Moderate risk: The randomized, double-blind design strengthens internal validity, but the very small sample size limits statistical power and precision of effect estimates. Phase 2 nature and exploratory efficacy outcomes reduce generalizability. Low event numbers increase uncertainty, and some missing outcome data may introduce minor attrition bias. |
| [Petersen AO, Damholt B, Grove M, Hink J, Marotte-Hurbon T, Söderqvist J, et al. Safety, recovery, and pharmacodynamics of CRISPR–Cas therapeutic SNIPR001: a phase 1, randomised, double-blind, first-in-human, dose-escalation study. Lancet Microbe. 2026 Mar 2;7(4):101257 doi:10.1016/j.lanmic.2025.101257](https://ad-id.co/4svinNC) | **P:** 36 healthy adults (mean age 42.1 years, 39% female) with high gut E. coli colonisation (>10^7 CFU/g stool)  **I:** Oral SNIPR001 bacteriophage cocktail (CRISPR–Cas-armed phages) at 10^8, 10^10, or 10^12 PFU twice daily for 7 days  **C:** Placebo (phosphate-buffered saline), dose-matched across cohorts  **O:** Safety and adverse events (primary); reduction in stool E. coli levels, microbiome composition changes, pharmacokinetics/biodistribution of SNIPR001 (secondary) | SNIPR001 was safe and well tolerated across all dose cohorts, with only mild to moderate adverse events and no grade 3–4 or serious adverse events attributed to treatment. The incidence of adverse events was not significantly higher than placebo (p=0.94), and most events occurred in placebo groups. Gut microbiome composition was not significantly altered by SNIPR001 compared with placebo. The phage was recovered from stool in a dose-dependent manner but was not detected in plasma or urine (except rare samples) and was undetectable by 6 months, supporting gut-restricted activity. A non-significant reduction in E. coli burden was observed at the highest dose (78% reduction; −0.65 log10 at day 14), but this did not reach statistical significance (p=0.811). | Moderate risk: The randomized, double-blind, placebo-controlled design supports internal validity, but the very small sample size and phase 1 dose-escalation structure limit statistical power and precision. Primary outcomes focus on safety rather than efficacy, restricting interpretability for clinical effectiveness. Cohort-based sequential enrolment may introduce selection or temporal bias, and exploratory microbiome/efficacy endpoints are underpowered with multiple comparisons. |
| [Li Y, Song L, Feng Z, Zhang X, Miao Y, Chen X, et al. Rifapentine dosing for drug-susceptible tuberculosis: stage 1 of a seamless phase 2/3 randomized clinical trial. Clin Microbiol Infect. 2026 Apr 11; doi:10.1016/j.cmi.2026.04.004](https://ad-id.co/423xJOr) | **P:** 400 adults with pulmonary drug-susceptible tuberculosis enrolled across 16 sites in China  **I:** 4-month rifapentine-based regimens (10, 15, or 20 mg/kg daily) combined with isoniazid, moxifloxacin, and pyrazinamide  **C:** Standard 6-month regimen (isoniazid, rifampicin, pyrazinamide, ethambutol)  **O:** Safety-related permanent treatment discontinuation by week 8; culture conversion by week 8; treatment outcomes at end of therapy | Safety-related discontinuation was higher in rifapentine groups compared with control, particularly at 20 mg/kg (14.6% vs 3.0%), with intermediate rates at 10 mg/kg (9.3%) and 15 mg/kg (8.9%). Culture conversion by week 8 was numerically higher in rifapentine 15 mg/kg (84.8%) and 20 mg/kg (87.9%) compared with control (78.0%), with 10 mg/kg showing lower conversion (73.8%). End-of-treatment favorable outcomes were similar across groups (≈81–87%), with no statistically significant differences in relative risk for any rifapentine dose versus control. Overall, higher rifapentine doses improved early microbiological response but increased safety-related treatment discontinuation. | Low to moderate risk: The randomized, multicentre, controlled design with adequate sample size improves internal validity. However, the open-label nature (not explicitly stated but typical for regimen trials) may introduce performance and detection bias, particularly for safety-related discontinuation. Early-phase (stage 1) adaptive design and multiple dose comparisons increase multiplicity risk. Follow-up to end-of-treatment outcomes is appropriate, but longer-term relapse outcomes are not reported, limiting completeness. |
| [Thorpe A, Lee RA, Szymczak JE, Farrell MC, Palmer I, Petty WB, et al. Comparing the antimicrobial resistance crisis to the coronavirus disease 2019 pandemic: a randomized public health messaging experiment. Clin Infect Dis. 2026 Apr 8; doi:10.1093/cid/ciag110](https://ad-id.co/4sAbjzo) | **P:** 972 US adults (mean age 42 years, 58% female) recruited in an online randomized survey  **I:** Public health messaging comparing antimicrobial resistance (AMR) to the COVID-19 pandemic (written message or infographic)  **C:** Standard written AMR information without COVID-19 comparison  **O:** Intentions to visit a clinician and to seek antibiotics for a hypothetical viral respiratory infection | There were no significant differences between intervention and control groups in intention to visit a primary care clinician (P = 0.625) or in desire to take antibiotics (P = 0.157). Overall, exposure to COVID-19 comparison messaging (written or infographic) did not reduce antibiotic-seeking intentions compared with standard AMR messaging. Exploratory analyses showed that older age and independent/third-party political affiliation were associated with lower antibiotic-seeking intentions, while medical maximizing tendencies, prior antibiotic use, COVID-19 vaccination status, and pride in vaccination were associated with higher antibiotic-seeking intentions. | Low to moderate risk: Randomized design with large sample and high completion rate (90%) supports internal validity. However, outcomes are self-reported behavioral intentions in a hypothetical scenario rather than actual behavior, limiting external validity. Online survey design may introduce selection bias and social desirability bias. Intervention exposure was brief and may have been insufficient to detect meaningful behavioural change. |
| [Regev-Yochay G, Barda N, Shusterman Y, Magiel E, Ganmore I, Baharav N, et al. West Nile virus–neutralizing plasma for West Nile virus disease. NEJM Evid. 2026 Mar 24;5(4). doi:10.1056/EVIDoa2500169](https://ad-id.co/4tLoVZh) | **P:** 34 hospitalized patients with laboratory-confirmed symptomatic West Nile virus (WNV) disease (median age 74 [IQR 64–82], 50% women; 91% with neuroinvasive disease; included ≥60 years or 18–59 immunocompromised).  **I:** Donor-derived WNV-neutralizing plasma (2:1 randomization; n=22).  **C:** Placebo (n=12).  **O:** Composite of all-cause mortality or functional deterioration (>5-point decline in Barthel Index) at 30 days; secondary outcomes included mortality, Barthel Index, and cognitive function (MMSE) at 30 and 90 days. | At 30 days, the primary composite outcome occurred in 50% of intervention vs 50% of placebo (RR 0.96, 95% CI 0.51–1.79), showing no difference. Mortality was numerically lower in the intervention group (2/22 vs 4/12; RR 0.33, 95% CI 0.09–1.15) but not statistically significant. Intervention was associated with improved functional capacity (Barthel Index 85 vs 78; RR 1.15, 95% CI 1.05–1.27) and higher cognitive scores (MMSE 25 vs 22; RR 1.24, 95% CI 1.04–1.47). One allergic reaction occurred in the treatment group. Overall, no clear clinical benefit on primary outcome but signals of functional and cognitive improvement. | High risk: Very small sample size (n=34) with wide confidence intervals and limited power increases risk of type II error and imprecision. Although randomized and double-blind design reduces performance and detection bias, the small cohort and 2:1 allocation reduce robustness of effect estimates. Some outcomes show borderline significance and may be influenced by chance. Short follow-up (30 days primary endpoint) limits longer-term inference. |

**Phage Therapy**

- [Bosco K, Petrovic Fabijan A, Iredell J, Dabrowska K, Khatami A. Immune responses to phage therapy in humans: a review. J Infect Dis. 2026 Feb 13; doi:10.1093/infdis/jiag096](https://ad-id.co/4sjHYJM)
  - Summary: This review examines mammalian, particularly human, immune responses to both endogenous and therapeutic phages, highlighting how patient-specific factors, treatment regimens, and phage characteristics influence phage clearance and pharmacokinetics, and emphasizes that a deeper understanding of these immune interactions is critical for developing targeted and effective personalized phage therapies to address the growing challenge of antimicrobial resistance.

**Antibiotic Therapy Reviews**

- [Islam R, Das SC, Pletzer D. Combating multidrug-resistant Klebsiella pneumoniae: current therapeutic regimens and future directions. J Antimicrob Chemother. 2026 Mar;81(3):dkag064; doi:10.1093/jac/dkag064](https://ad-id.co/47gxXVm)
  - Summary: This review addresses the escalating threat of multidrug-resistant Klebsiella pneumoniae in hospital-acquired pneumonia, analyzing resistance mechanisms such as blaKPC, blaNDM, and mcr genes, porin mutations, efflux pump overexpression, and enzymatic degradation, evaluates the efficacy and controversies of double and triple antibiotic combination therapies, and highlights inhaled antibiotic delivery—especially dry powder inhalers—as a promising strategy to achieve effective pulmonary drug concentrations with reduced systemic toxicity, while noting ongoing challenges including formulation stability, regulatory hurdles, and limited clinical trials, and emphasizing the need for future research on optimized inhaled therapies and innovative delivery platforms.
- [Fukuda D, Powell D, Mulgirigama A, Vojtek I, Ozeki H, Yoshimoto D, et al. Bacterial DNA topoisomerase IV and DNA gyrase inhibitors: history of the quinolones, their clinical usage and potential alternatives for the future. J Antimicrob Chemother. 2026 Mar;81(3):dkag054; doi:10.1093/jac/dkag054](https://ad-id.co/4soFmKF)
  - Summary: This review discusses bacterial topoisomerases as essential targets for antibacterial therapy, detailing the development and mechanisms of quinolones and fluoroquinolones—which inhibit DNA gyrase and topoisomerase IV to disrupt DNA replication—and highlights their broad-spectrum efficacy, chemical stability, and continued clinical importance, while addressing concerns over side effects, rising fluoroquinolone resistance, and the urgent need for new non-quinolone topoisomerase inhibitors such as zoliflodacin, gepotidacin, and fobrepodacin to combat antimicrobial resistance and ensure future treatment options.

**PK/PD and Drug Dosing**

- [O’Keefe K, Denny KJ, Le Marsney R, McCullough J, Gilholm P, Budai KA, et al. Prolonged versus intermittent beta-lactam antibiotic infusions in paediatric critical care: a systematic review and meta-analysis. J Paediatr Child Health. 2025 Dec 27;62(2):160-170; doi:10.1111/jpc.70275](https://ad-id.co/3OQkTQf)
  - Summary: This systematic review and meta-analysis of 10 observational studies in paediatric intensive care unit patients found no significant reduction in mortality with prolonged versus intermittent beta-lactam infusions (OR 0.60, 95% CI 0.24–1.51), identified insufficient data to assess other outcomes such as length of stay and PK/PD benefits, and concluded that current evidence is limited and of low quality, underscoring the need for further high-quality research to determine potential clinical advantages in critically ill children.
- [Chastain DB, Anderson DT, Eudy J, Henao-Martínez AF, Cluck DB. Precision dosing of systemic antifungals in adults: therapeutic drug monitoring, empiric dose optimization and barriers to implementation. J Antimicrob Chemother. 2026 Mar;81(3):dkag055; doi:10.1093/jac/dkag055](https://ad-id.co/406CndH)
  - Summary: This review highlights the critical role of therapeutic drug monitoring (TDM) in optimizing systemic antifungal therapy by enabling individualized dosing to improve efficacy, reduce toxicity, and prevent resistance, detailing the need for routine TDM with triazoles like voriconazole, posaconazole, and itraconazole due to interpatient variability, selective monitoring for fluconazole and isavuconazole in high-risk scenarios, mandatory monitoring for flucytosine, limited TDM utility for echinocandins and liposomal amphotericin B, and the current lack of data for emerging agents, while emphasizing that real-world TDM use remains inconsistent and calling for improved access, standardized protocols, and consensus guidance to ensure safe and effective antifungal therapy.
- [Li W, Pang Y, Wang P, Liu J, Gao C, Liu W, et al. Physiologically based pharmacokinetic modelling to optimize dosing regimen of biapenem in renal impairment and elderly populations. J Antimicrob Chemother. 2026 Mar;81(3):dkaf499; doi:10.1093/jac/dkaf499](https://ad-id.co/400MjW7)
  - Summary: This study developed and validated physiologically based pharmacokinetic (PBPK) models for biapenem in healthy adults, renal impairment, and elderly populations, recommending dose reductions to 67%, 50%, and 30% of the standard adult dose for moderate, severe, and end-stage renal impairment respectively, with no adjustment for mild impairment, showing that most simulated regimens achieved effective cumulative fraction of response against Escherichia coli and Klebsiella pneumoniae but were insufficient for Pseudomonas aeruginosa and Acinetobacter baumannii, demonstrating that PBPK modeling can guide optimized biapenem dosing in special populations.
- [Chinello P, Gavaruzzi F, Galati V, Tempestilli M, Lauri C, De Nicolò A, et al. An inoperable mediastinal infection involving vascular graft treated with weekly oritavancin as chronic suppressive therapy: a case report with pharmacokinetic data. J Antimicrob Chemother. 2026 Mar;81(3):dkag013; doi:10.1093/jac/dkag013](https://ad-id.co/3PbplZW)
  - Summary: This report highlights the emerging off-label use of long-acting lipoglycopeptide antibiotics, dalbavancin and oritavancin, for prolonged infections such as endocarditis, osteomyelitis, and prosthetic device infections, noting that most evidence and pharmacokinetic data pertain to dalbavancin, and presents a case of a patient with an inoperable mediastinal infection involving vascular prostheses treated with chronic suppressive oritavancin therapy, including pharmacokinetic observations.
- [De Clercq A, Desmet T, Boelens J, Somers A, Stove V, Verougstraete N, et al. Risk factors for suboptimal target attainment of commonly used β-lactam antibiotics in older adults: a prospective cohort study. J Antimicrob Chemother. 2026 Mar;81(3):dkag048; doi:10.1093/jac/dkag048](https://ad-id.co/4se9AQf)
  - Summary: This prospective study in geriatric inpatients (median age 87 years) found that standard intravenous dosing of amoxicillin-clavulanate and piperacillin-tazobactam frequently failed to achieve PK/PD targets (100% fT > MIC), with comorbidity burden and renal function, rather than chronological age, identified as key risk factors for target non-attainment, and no patients exceeded toxicity thresholds, highlighting the need for further research to identify predictors of interpatient variability and to develop evidence-based dosing strategies in older adults.
- [Bergmann F, Prager M, Pracher L, Jorda A, Haselwanter P, Zeitlinger M. Tissue pharmacokinetics of antifungal drugs: a review. Int J Antimicrob Agents. 2026 Feb 12;107743; doi:10.1016/j.ijantimicag.2026.107743](https://ad-id.co/4sdHMeN)
  - Summary: This review highlights that tissue penetration of antifungal agents—including triazoles, echinocandins, amphotericin B formulations, and flucytosine—varies widely across compartments and is not reliably predicted by plasma concentrations, with AUC-based tissue penetration ratios offering more clinically relevant insights, emphasizing that optimizing antifungal dosing in immunocompromised and critically ill patients requires consideration of tissue-specific pharmacokinetics to ensure therapeutic success.
- [Uemura O. Differences in international recommendations for piperacillin dosing: a Japanese perspective. JMA J. 2026 Jan 15;9(1):366-368; doi:10.31662/jmaj.2025-0414](https://ad-id.co/4sDP24b)
  - Summary: This article examines the contrasting piperacillin dosing recommendations between international guidelines, which advocate high-dose regimens (13.5–27 g/day) in critically ill ICU patients to achieve pharmacokinetic/pharmacodynamic targets, and Japanese labeling, which recommends lower doses (2–4 g/day, max 16 g/day) to prioritize safety in general wards, highlighting that indiscriminate application of either approach can lead to adverse events, therapeutic failure, or antimicrobial resistance, and emphasizing that clinicians should tailor dosing based on patient severity and clinical context to balance efficacy, safety, and stewardship.
- [Pillay-Fuentes Lorente V, Abulfathi AA, Marais JS, De Jong J, Kellermann T, Mashishi D, et al. Pharmacokinetics of colistin in adult critically ill patients in South Africa. J Antimicrob Chemother. 2026 Mar;81(3):dkag011; doi:10.1093/jac/dkag011](https://ad-id.co/4sgAOFQ)
  - Summary: This prospective study in 24 critically ill South African adults, half with burns, characterized the pharmacokinetics of intravenous colistimethate sodium (CMS) and colistin, finding that PK parameters after a 9 MU loading dose were comparable to published data, that colistin AUC negatively correlated with white cell count and eGFR, and that target attainment was adequate for Acinetobacter baumannii MICs <1 mg/L, highlighting the need for future population pharmacokinetic modeling to guide individualized dosing in this population.
- [Vallé Q, Sharma R, Bui NM, Schaefer A, Mahadevan R, Mojica MF, et al. Evaluating the antibacterial activity of ceftazidime/avibactam and aztreonam combinations against multidrug-resistant *Stenotrophomonas maltophilia* complex isolates in a hollow fiber infection model. Clin Microbiol Infect. 2026 Feb 14; doi:10.1016/j.cmi.2026.02.010](https://ad-id.co/4snYvwm)
  - Summary: This study evaluated aztreonam (ATM) combined with ceftazidime/avibactam (CZA) against multidrug-resistant Stenotrophomonas maltophilia complex isolates, showing that clinically relevant ATM+CZA exposures produced 31–74% reductions in bacterial AUC in static time-kill assays and ≥3-log10 CFU/mL reductions in hollow fiber infection models, effectively suppressing resistant subpopulations and demonstrating therapeutic potential, while highlighting the need for further evaluation in murine pneumonia and bacteremia models due to the pathogen’s intrinsic resistance mechanisms.
- [Gatti M, Rinaldi M, Laici C, Ambretti S, Siniscalchi A, Viale P, et al. Biliary pharmacokinetic/pharmacodynamic analysis of continuous infusion ceftazidime–avibactam in a case series of orthotopic liver transplant recipients. J Antimicrob Chemother. 2026 Mar;81(3):dkag009; doi:10.1093/jac/dkag009](https://ad-id.co/3N70FRT)
  - Summary: This exploratory case series in four critical orthotopic liver transplant recipients assessed biliary pharmacokinetics and pharmacodynamics of continuous infusion ceftazidime–avibactam, finding moderate and broadly similar bile penetration for both drugs (median bile-to-plasma ratios 0.28 for ceftazidime and 0.24 for avibactam), with aggressive joint PK/PD targets achieved in half of the cases, suggesting that continuous infusion may help optimize biliary exposure against pathogens with MICs up to 8 mg/L.
- [Lin T, Senneville E, Hennart B, Valentin B, Lafon-Desmurs B, Boucher A, et al. Real-world drug monitoring of dalbavancin using a three-dose regimen of 1500 mg at days 1, 15 and 43 in bone and joint or cardiovascular infection. J Antimicrob Chemother. 2026 Mar;81(3):dkag039; doi:10.1093/jac/dkag039](https://ad-id.co/4l3xbAL)
  - Summary: This retrospective study of 42 patients with bone, joint, or vascular infections found that a three-dose dalbavancin regimen (1500 mg on days 1, 15, and 43) maintained serum concentrations above the validated target of 8.04 mg/L over a 12-week treatment period, including in patients with hypoalbuminemia or obesity, suggesting this regimen can achieve sustained therapeutic exposure for Staphylococcus spp. infections with MIC ≤0.125 mg/L, while highlighting the potential need for therapeutic drug monitoring in special populations or for higher-MIC strains.
- [Monti B, Ricci E, Mariani M, Mesini A, Saffioti C, Cafaro A, et al. The ‘double hit’ on dalbavancin pharmacokinetics: hypertriglyceridaemia and augmented renal clearance in a child with glycogen storage disease type Ib. J Antimicrob Chemother. 2026 Mar;81(3):dkag028; doi:10.1093/jac/dkag028](https://ad-id.co/3N93Los)
  - Summary: This case report describes a 6-year-old boy with glycogen storage disease type Ib and left orbital cellulitis who exhibited marked pharmacokinetic variability with dalbavancin due to metabolic dysregulation and augmented renal clearance, highlighting the challenges of off-label multidose dalbavancin therapy in pediatric patients with complex metabolic disorders to provide prolonged outpatient coverage for Gram-positive infections, including early osteomyelitis.

**Beta-Lactamases and Other Resistance Mechanisms**

- [Zz Xi J, Jia X, Li S, Li X, Hu L, Xia H, et al. A nationwide genomic surveillance study of Gram-negative bacteria causing community- and hospital-acquired bloodstream infections in China. J Infect. 2026 Feb;92(2):106693; doi:10.1016/j.jinf.2026.106693](https://ad-id.co/4sdpL0g)
  - Summary: This genomic epidemiology study of 1,934 Gram-negative bloodstream infection isolates from 21 Chinese teaching hospitals revealed that community-acquired (CA) and hospital-acquired (HA) infections exhibit distinct clinical and molecular profiles, with CA-BSIs showing elevated inflammatory markers and virulent lineages such as ST131 E. coli and ST23 K. pneumoniae, while HA-BSIs are dominated by high-risk, antimicrobial-resistant clones like ST11 K. pneumoniae and ST2 A. baumannii associated with worse prognosis, and multivariable analysis identified setting-specific mortality risk factors influenced by patient comorbidities and pathogen genetic determinants, highlighting the need for targeted genomic surveillance to improve outcomes.

**Antibiotics - In vitro susceptibility**

- [Fan Y, Liang X, Ren Y, Nie S, Li H, Xie J, et al. A two-stage diagnostic model for discriminating and assessing risk of meropenem heteroresistance in *Pseudomonas aeruginosa*. J Antimicrob Chemother. 2026 Mar;81(3):dkag061; doi:10.1093/jac/dkag061](https://ad-id.co/4smsuon)
  - Summary: This retrospective study of 420 Pseudomonas aeruginosa strains from China identified stage-specific risk factors for meropenem heteroresistance—central venous catheters for susceptible-to-heteroresistant (S-HR) and malignant solid tumors, pulmonary infections, mechanical ventilation, and prior carbapenem use for heteroresistant-to-resistant (HR-R) transitions—and developed a validated two-stage diagnostic model combining clinical and laboratory data with high discriminative performance (AUROC 0.856 for S-HR and 0.919 for HR-R), providing a practical tool for early detection and targeted management of heteroresistant infections.
- [Chen J, Sun Z, Su J, Li P, Xu X, Wang M. Functional characterization of a novel class A carbapenemase CAE-1 in carbapenem-resistant *Pseudomonas aeruginosa* clinical isolates. Antimicrob Agents Chemother. 2026 Feb 23;e01362-25; doi:10.1128/aac.01362-25](https://ad-id.co/3OTagw2)
  - Summary: This study characterized two carbapenem-resistant Pseudomonas aeruginosa isolates lacking known carbapenemases but producing the class A β-lactamase CAE-1, demonstrating that CAE-1 confers broad β-lactam resistance—including an eightfold increase in meropenem MIC in P. aeruginosa—while remaining susceptible to ceftazidime-avibactam, exhibits catalytic activity against multiple β-lactams with lower efficiency for carbapenems compared to KPC-2, and is encoded on an integrative and conjugative element, highlighting its potential for horizontal transfer and the need for enhanced surveillance of blaCAE-1-harboring strains.
- [Yu X, Liu Y, Du J, Hu F, Yin D. Assessment of the revision of the 2025 CLSI breakpoints for the interpretation of minocycline susceptibility for *Acinetobacter baumannii* complex. Microbiol Spectr. 2026 Feb 13; doi:10.1128/spectrum.02700-25](https://ad-id.co/47iI8Zz)
  - Summary: This study evaluated the impact of the 2025 CLSI breakpoint revisions for minocycline on 276 Acinetobacter baumannii complex isolates in China, finding that the updated breakpoints markedly reduced susceptibility rates (from 73.9% to 46.4% overall and from 53.3% to 6.0% for carbapenem-resistant A. baumannii) and increased resistance rates, while improving categorical agreement between disk diffusion and broth microdilution from 64.1% to 90.9% and reducing intermediate results, supporting the clinical applicability of the revised breakpoints and recommending BMD confirmation for isolates categorized as intermediate by disk diffusion.
- [Kim SH, Kim HM, Chung DR, Ko JH, Huh K, Cho SY, et al. In vitro activity of double and triple antimicrobial combinations against carbapenem-resistant *Pseudomonas aeruginosa* biofilm. J Antimicrob Chemother. 2026 Mar;81(3):dkag031; doi:10.1093/jac/dkag031](https://ad-id.co/401fOqT)
  - Summary: This study evaluated 12 carbapenem-resistant Pseudomonas aeruginosa bloodstream isolates and found that minimum biofilm eradication concentrations (MBECs) of rifampin, colistin, imipenem, and ceftazidime/avibactam were significantly higher than planktonic MICs, but approximately half of tested double and triple antibiotic combinations demonstrated synergistic activity against biofilms, with triple combinations generally showing lower fractional biofilm eradication indices, providing experimental evidence that combination therapy may be more effective than single agents for eradicating CRPA biofilms and supporting further investigation in biofilm-associated infections.

**Adverse effects from antimicrobial agents**

- [Joean O, Sermet K, Ashkenazi-Hoffnung L, Cakir Kiymaz Y, Blumenthal K, Bonazzetti C, et al. ESCMID clinical guidelines on the evaluation and management of a reported antibiotic allergy. Clin Microbiol Infect. 2026 Feb 16; doi:10.1016/j.cmi.2026.02.011](https://ad-id.co/4l4Mmdc)
  - Summary: The ESCMID guideline provides evidence-based recommendations for evaluating and managing reported antibiotic allergies, emphasizing that most documented allergies, particularly to beta-lactams, are not true immune-mediated hypersensitivities, and advocating structured clinical assessment, direct delabelling, or controlled drug challenges for low-risk cases, with the goals of improving patient outcomes, reducing unnecessary broad-spectrum antibiotic use, and supporting antimicrobial stewardship to mitigate the development of antimicrobial resistance.
- [Pham HT, Truong-Nguyen KH, Tran MH. Echinocandins and coagulation dysfunction events. Thrombosis J. 2026 Jan 5;24:1; doi:10.1186/s12959-025-00774-0](https://ad-id.co/404caMY)
  - Summary: This analysis critiques recent real-world pharmacovigilance studies linking echinocandins to coagulation dysfunction, highlighting concerns including the combined categorization of hyper- and hypocoagulation, potential misclassification of thrombocytopenia, lack of adjustment for confounders, inappropriate statistical methods, and misleading interpretations of time-to-onset and predictability, emphasizing that addressing these methodological limitations is necessary to strengthen understanding of echinocandin-associated coagulation adverse events and guide further investigation.

**Antibiotic Stewardship and Hospital in the Home**

- [Abdulaziz S, Alhajri HS, So M, Butt S, Zaaqoq AM, Asiri A, et al. Antimicrobial stewardship during extracorporeal membrane oxygenation: challenges and new perspectives. Clin Microbiol Infect. 2026 Feb 25; doi:10.1016/j.cmi.2026.02.021](https://ad-id.co/3NcjXFt)
  - Summary: This review highlights that extracorporeal membrane oxygenation (ECMO) alters antimicrobial pharmacokinetics and pharmacodynamics, increasing the risk of underdosing or toxicity, and complicates antibiotic therapy in critically ill patients, emphasizing that judicious antimicrobial stewardship—including protocol-driven prophylaxis, therapeutic drug monitoring, timely de-escalation, and interprofessional programs—combined with strict non-pharmacologic infection control measures, can reduce antimicrobial consumption and resistance without compromising outcomes, while acknowledging persistent knowledge gaps in optimal dosing and stewardship strategies for ECMO patients.
- [Hughes S, Cheong J, Snape J, Jethwa S, Ng SH, Thangarajah R, et al. Multicentre evaluation of teicoplanin prescribing and monitoring in the UK and Ireland: the TUcK–SHOP study. J Antimicrob Chemother. 2026 Mar;81(3):dkag038; doi:10.1093/jac/dkag038](https://ad-id.co/4spbhe1)
  - Summary: This multicentre retrospective study of 391 patients across 21 UK and Irish hospitals found significant variation in teicoplanin prescribing and therapeutic drug monitoring, with overall guideline adherence at 66% and only 40.8% of patients on 6 mg/kg maintenance achieving target trough levels (≥20 mg/L) compared with 86.6% on 12 mg/kg, while predictors of higher trough levels included lower creatinine clearance, longer time to TDM, higher doses, and greater body weight, highlighting the need for standardized dosing protocols to optimize teicoplanin therapy and improve target attainment.
- [Foong KS, Fowle L, Slider A, Campion M, Leaf J, Cumming M, et al. A public health approach to antimicrobial stewardship in long-term care facilities: a multifaceted program in Massachusetts. Clin Infect Dis. 2026 Feb 13; doi:10.1093/cid/ciag092](https://ad-id.co/4aSJQU1)
  - Summary: The Massachusetts Antibiotic Start (AS) Reporting Program, implemented across 217 long-term care facilities from 2018 to 2024, demonstrated that sustained benchmarking, feedback, educational activities, and recognition can improve antimicrobial stewardship, with overall AS rates slightly increasing, fluoroquinolone starts decreasing by 36%, and beta-lactam starts rising by 26%, while interrupted time series analysis revealed a significant uptick in beta-lactam use following the 2022 program expansion, highlighting the effectiveness of data-driven public health-academic collaboration in promoting engagement and sustainable improvements in antibiotic prescribing in resource-limited settings.
- [Peltan ID, Groat D, Butler J, Bledsoe JR, Ofori-Atta BS, Wu C, et al. Physician variation in early sepsis management. JAMA Netw Open. 2026 Feb 13;9(2):e2556945; doi:10.1001/jamanetworkopen.2025.56945](https://ad-id.co/4sj1wh5)
  - Summary: In a mixed-methods study of 9810 sepsis patients and 88 ED physicians across four Utah hospitals, physician-level door-to-antimicrobial times varied significantly, but faster antimicrobial initiation was not associated with overtreatment; qualitative interviews revealed that physicians with shorter times employed proactive, parallel task execution and team coordination, whereas slower physicians used stepwise, reactive approaches, suggesting that individual and team-based strategies can expedite sepsis care without increasing unnecessary antimicrobial use.
  - **Editorial Commentary:**[Gershengorn HB. How physician heterogeneity may matter in sepsis care. JAMA Netw Open. 2026 Feb 13;9(2):e2556868; doi:10.1001/jamanetworkopen.2025.56868](https://ad-id.co/3N7sFoq)

**Infection Prevention / Antibiotic Prophylaxis**

- [Chu VT, Spottiswoode N, Ward R, Yokoe DS, Ramirez-Avila L, Phelps MS, et al. Implementation and outcomes of a rapid response genomic hospital epidemiology programme at an academic medical centre over 7 years. Lancet Microbe. 2026 Jan 6;7(2):101277; doi:10.1016/j.lanmic.2025.101277](https://ad-id.co/47hRip8)
  - Summary: The Rapid Response (RR) genomic epidemiology programme at University of California, San Francisco, leverages whole-genome sequencing (WGS) and metagenomic next-generation sequencing (mNGS) to investigate suspected hospital outbreaks, monitor emerging pathogens, and conduct surveillance of high-priority microbes; over seven years, the programme conducted 67 investigations and four surveillance initiatives, predominantly bacterial, demonstrating that rapid genomic analyses can rule out transmission, detect changing pathogen incidence, guide infection-prevention responses, and reduce resource-intensive investigations, providing a practical framework for integrating genomics into hospital infection control and patient safety efforts.
- [Motaghi S, Karam SG, Mulazzani F, Mirzayeh Fashami F, Buchan TA, Ibrahim S, et al. Antibiotic prophylaxis strategies and surgical site infections in colorectal surgery: a systematic review and network meta-analysis. JAMA Netw Open. 2026 Feb 19;9(2):e2560095; doi:10.1001/jamanetworkopen.2025.60095](https://ad-id.co/400sC0C)
  - Summary: This systematic review and network meta-analysis of 105 randomized clinical trials involving 18,273 patients found that broad-spectrum penicillins and cephalosporin-based regimens were most strongly associated with reduced surgical site infection (SSI) risk after elective colorectal surgery, with broad-spectrum penicillins also linked to decreased 30-day mortality; no significant differences were observed in hospital length of stay or adverse events, highlighting antibiotic class selection as a key factor in optimizing perioperative prophylaxis.
  - **Editorial Commentary:**[Wilson NA. Antibiotic selection and redosing to prevent surgical site infections—when timing is not enough. JAMA Netw Open. 2026 Feb 18;9(2):e2559356; doi:10.1001/jamanetworkopen.2025.59356](https://ad-id.co/404ln7S)
- [Dureau P, Rombi L, Ouorou R, et al. Preoperative and Intraoperative Risk Factors for Postoperative Pneumonia After Cardiac Surgery: An Ancillary Study of the STERNOCAT (Catheter Outcomes With Sternotomy Cardiac Operated) Randomized Trial and a Systematic Review With Meta-Analysis. Crit Care Med. 2026 Feb 11. doi: 10.1097/CCM.0000000000007060](https://ad-id.co/4skVs81)
  - Summary: In a cohort of 1,470 cardiac surgery patients and a systematic review of 24 studies including 172,079 patients, postoperative pneumonia (POP) occurred in 5.3% of cases and was associated with higher 30-day mortality; independent risk factors included ischemic cardiomyopathy, prolonged cardiopulmonary bypass, and catecholamine use, while most identified risk factors were non-modifiable, emphasizing the need for perioperative optimization of modifiable factors such as CPB duration and transfusion practices to reduce POP risk.

**Sexually transmitted infections**

- [Unemo M, Golparian D, Elango V, Bettiol E, Piddock LJV, Srinivasan S, et al. Microbiological analysis and whole-genome sequencing of *Neisseria gonorrhoeae* from the microbiological failures in the international, zoliflodacin, phase 3, clinical trial for treatment of uncomplicated urogenital gonorrhoea: a retrospective, genomic, observational study. Lancet Microbe. 2026 Feb 5;7(2):101270; doi:10.1016/j.lanmic.2025.101270](https://ad-id.co/4sbcJ3s)
  - Summary: In a phase 3 trial of zoliflodacin for uncomplicated urogenital gonorrhoea, whole-genome sequencing of baseline and test-of-cure isolates from 960 samples showed that 23% of zoliflodacin microbiological failures and the single ceftriaxone–azithromycin failure involved different strains, suggesting reinfection rather than treatment failure; no mutations conferring zoliflodacin resistance were detected, and 59% of zoliflodacin failures in anogenital sites had low MICs, supporting high efficacy and highlighting the value of WGS in future gonorrhoea trials to distinguish reinfection from true treatment failure.
- [Lin LW, Chen IS, Pai SY, Lu CH, Hsieh CH, Lin KY, et al. A silent spirochete: *Treponema pallidum* in seronegative syphilis. Clin Microbiol Infect. 2026 Feb 20; doi:10.1016/j.cmi.2026.02.014](https://ad-id.co/3ZZRNAt)
  - Summary: This article describes cases of Treponema pallidum infection presenting as seronegative syphilis, where standard serological tests initially failed to detect antibodies despite clinical evidence of infection, and highlights the utility of T. pallidum PCR assays for diagnosis—especially in early stages or atypical presentations—as exemplified by a case where a chemiluminescent immunoassay became positive only six months after treatment, underscoring that molecular testing can identify infections missed by serology alone and may improve diagnostic accuracy.
- [Pugsley RA, Davis NL, Cope AB, Diesel JC, Matthias J, Danforth B, et al. Is three really what we need? Relative effectiveness of benzathine penicillin G and doxycycline treatment regimens for late or unknown duration syphilis in 6 United States jurisdictions, 2016–2021. Clin Infect Dis. 2026 Feb 23; doi:10.1093/cid/ciag099](https://ad-id.co/404mC76)
  - Summary: This retrospective cohort study of 18,027 late or unknown duration syphilis cases found no difference in treatment effectiveness—defined as a fourfold decline in non-treponemal titers—between 1 versus 3 doses of benzathine penicillin G or between 3 doses of benzathine penicillin G and doxycycline, with similar outcomes across baseline titer, pregnancy status, and HIV infection, suggesting that a single dose may be as effective as multiple doses.
  - **Editorial Commentary:**[Kojima N, Klausner JD. Strong real-world evidence that a single dose of benzathine penicillin G or a 28-day course of oral doxycycline is not inferior to three doses of benzathine penicillin G for the treatment of late latent or syphilis of unknown duration. Clin Infect Dis. 2026 Feb 23; doi:10.1093/cid/ciag101](https://ad-id.co/40BS8JF)
- [Coldbeck-Shackley RC, Flynn E, Mudhar AK, Taouk ML, Taiaroa G, Bell C, et al. Increased diversity and introduction of multidrug-resistant strains of Neisseria gonorrhoeae following cessation of COVID-19 pandemic–related travel restrictions: an observational genomic epidemiologic study. J Infect Dis. 2026 Feb 12; doi:10.1093/infdis/jiag097](https://ad-id.co/4shCM99)
  - Summary: Genomic analysis of Neisseria gonorrhoeae in South Australia and Victoria showed that the bacterium was highly clonal during COVID-19 travel restrictions, with genomic diversity increasing after restrictions were lifted, highlighting the value of routine genomic surveillance to monitor the introduction and spread of antimicrobial-resistant strains.
- [Prazuck T, Moal GLE, Ursenbach A, Michau C, Perfezou P, Bisio F, et al. Systematic three-site vs. classical single-site screening for C. trachomatis and N. gonorrhoeae infections in non-sex worker women: the multicentric SIST’RS study. Clin Microbiol Infect. 2026 Feb 12; doi:10.1016/j.cmi.2026.02.006](https://ad-id.co/4siX5mE)
  - Summary: A prospective study of 1,498 women attending free STI screening centers in France found that systematic three-site testing (oral, anal, vaginal) significantly increased detection of Chlamydia trachomatis and Neisseria gonorrhoeae compared with vaginal-only testing, with 10–17% of infections detected exclusively at anal or oral sites, supporting the implementation of multisite screening in women regardless of reported sexual practices.
- [Campbell P. The influence of national antibiotic consumption on Neisseria gonorrhoeae antibiotic resistance in Norway, 2003–2024. J Infect Dis. 2026 Feb 11; doi:10.1093/infdis/jiag076](https://ad-id.co/47kWIjh)
  - Summary: Analysis of Norwegian Neisseria gonorrhoeae isolates from 2003–2024 showed that national consumption of penicillins and tetracyclines was strongly associated with increased gonococcal MICs and plasmid-mediated resistance, suggesting that even in low-use settings, population-level antibiotic use drives resistance, and the novel SIPI and WIPI metrics can help monitor shifts in susceptibility distributions for AMR surveillance.

**CNS Infections**

- [Lazarus G, Caddey B, Dean A, Febrina F, Wangsaputra VK, Radiani S, et al. Antimicrobial resistance in bacterial meningitis caused by Streptococcus pneumoniae, Neisseria meningitidis, or Haemophilus influenzae (2010–24): a systematic review and meta-analysis. Lancet Microbe. 2026 Jan 27; doi:10.1016/j.lanmic.2025.101238](https://ad-id.co/4scbe4W)
  - Summary: A systematic review of 16,441 meningitis isolates from 37 countries found high and regionally variable antimicrobial resistance, with benzylpenicillin resistance in Streptococcus pneumoniae increasing in LMICs, and notable resistance in Neisseria meningitidis and Haemophilus influenzae, highlighting the need for strengthened AMR surveillance to guide treatment, especially in resource-limited settings.
- [Pinell-McNamara VA, Ouédraogo HS, Kafando L, Sani O, Issoufou AA, Tassiou EI, et al. Bacterial meningitis epidemiology in Burkina Faso and Niger - high-risk countries in the meningitis belt of Sub-Saharan Africa, 2015-2023. J Infect Dis. 2026 Feb 24; doi:10.1093/infdis/jiag116](https://ad-id.co/4shHCDe)
  - Summary: Between 2015 and 2023, Burkina Faso and Niger experienced ongoing meningitis caused by Neisseria meningitidis, Streptococcus pneumoniae, and Haemophilus influenzae, with serogroup-specific patterns (Nm W in Burkina Faso, Nm C in Niger), highlighting that the introduction of Men5CV conjugate vaccine could reduce disease burden from serogroups C, W, and X.
- [Marín JEO, Boza MJL, Camargo CH, Campos KR, Santos MBN, Yamada AY, et al. Stepwise evolution of triple antimicrobial resistance in Neisseria meningitidis. J Infect Dis. 2026 Feb 23; doi:10.1093/infdis/jiag113](https://ad-id.co/4r7xBrw)
  - Summary: Surveillance in El Salvador identified invasive Neisseria meningitidis serogroup Y isolates with concurrent resistance to penicillin, ciprofloxacin, and tetracycline, mediated by blaROB-1, gyrA T91I, and tetB, representing a significant escalation in meningococcal antimicrobial resistance and highlighting the need for updated treatment and prophylaxis strategies.
- [Contou D, Painvin B, Daubin D, Orieux A, Pirollet H, Cour M, et al. Invasive meningococcal disease in adults aged ≥65 years admitted to French intensive care units: a nationwide comparison with younger adults. Clin Infect Dis. 2026 Feb 24; doi:10.1093/cid/ciag133](https://ad-id.co/47d42gL)
  - Summary: Critically ill older adults (≥65 years) with invasive meningococcal disease have atypical presentations, a high prevalence of serogroup W, require more organ support, and experience nearly triple the in-hospital mortality of younger patients, highlighting the need for rapid antibiotic therapy and consideration of expanded vaccination strategies for older populations.
- [Duerlund LS, Larsen L, Storgaard M, Mens H, Wiese L, Jepsen MP, et al. Herpes simplex virus type 1 encephalitis: a prospective population-based cohort study. Clin Infect Dis. 2026 Feb 23; doi:10.1093/cid/ciag136](https://ad-id.co/4sjbrDw)
  - Summary: In Denmark, HSV-1 encephalitis is rare but carries high mortality, with delayed acyclovir treatment—particularly when initiated more than 6 hours after lumbar puncture—associated with significantly increased 6-month mortality, underscoring the importance of rapid diagnosis and prompt antiviral therapy.
- [Subramanian AK, Java A, Gupta SK, Gupta S, Bomback AS. Strategies to mitigate infection risk in patients receiving complement inhibitor therapy. Clin Infect Dis. 2026 Feb 18; doi:10.1093/cid/ciag081](https://ad-id.co/4sgZjCX)
  - Summary: Complement inhibitors can increase susceptibility to serious infections from encapsulated bacteria, particularly meningococci; prevention relies on adherence to recommended vaccinations, judicious use of antibiotic prophylaxis tailored to local resistance patterns, and prompt evaluation of concerning symptoms, though evidence gaps leave some decisions—such as prophylaxis duration—up to clinical judgment.
- [Frusteri M, Hoyos DZ, Zapata MG, Suarez Sepúlveda JE, Quiceno E, Muñoz Ramírez F, et al. Effectiveness of prophylactic antibiotics for the prevention of intracranial infections following penetrating traumatic brain injury: a systematic review and meta-analysis. Neurosurg Rev. 2026;49:232. doi:10.1007/s10143-026-04139-7](https://ad-id.co/4sl1ky1)
  - Summary: Prophylactic antibiotics in penetrating traumatic brain injury do not significantly reduce the risk of intracranial infections, with no clear benefit from multi-drug or longer regimens; if used, short-course monotherapy is recommended, highlighting the need for high-quality standardized trials to guide clinical practice.

**Bloodstream Infections and Endocarditis**

- [Cohen A, Temkin E, Schwaber MJ, Carmeli Y, et al. The association between antibiotic use and the incidence of third-generation cephalosporin-resistance in Escherichia coli bloodstream infections: an ecological study. Antibiotics. 2026;15(2):187. doi:10.3390/antibiotics15020187](https://ad-id.co/400MkcD)
  - Summary: During the COVID-19 pandemic in Israel, decreased outpatient antibiotic use was followed by a reduction in the proportion of Escherichia coli bloodstream infections that were third-generation cephalosporin-resistant, with resistance rising again post-pandemic in association with increased antibiotic consumption.
- [Soo JZY, Lim TP, Ho JJY, Tong SYC, Lye DC, Davis JS, et al. Effect of in vitro synergy and additivity of vancomycin or daptomycin plus an anti-staphylococcal β-lactam for methicillin-resistant Staphylococcus aureus bacteraemia on mortality: pre-planned analysis from CAMERA2. Clin Microbiol Infect. 2026 Feb 10; doi:10.1016/j.cmi.2026.01.032](https://ad-id.co/406Cnud)
  - Summary: In the CAMERA2 trial, methicillin-resistant Staphylococcus aureus (MRSA) bloodstream infection patients whose isolates showed positive in vitro interactions between vancomycin or daptomycin and a β-lactam had lower 14-day mortality, suggesting combination therapy may be beneficial when synergy is present, though findings are hypothesis-generating and require further confirmation.
- [Leding C, Meddis A, Holler JG, Burisch J, Kragstrup TW, Skov L, et al. Tumor necrosis factor-α inhibitors, immune-mediated inflammatory diseases, and bloodstream infections: results from a nationwide case-control study. Clin Microbiol Infect. 2026 Feb 11; doi:10.1016/j.cmi.2026.02.005](https://ad-id.co/4sdpLgM)
  - Summary: In this nationwide Danish study, tumor necrosis factor-α inhibitor (TNFi) use was associated with increased odds of bloodstream infections (BSI), particularly for adalimumab and infliximab, with the highest risk in patients with inflammatory bowel disease and in those receiving concomitant glucocorticoids; odds varied by pathogen and underlying disease, highlighting the need for individualized infection risk assessment when prescribing TNFi.
- [Sánchez-Osuna M, Bravo M, Cañas M-A, Gómez A-C, Gómez-Sánchez I, Miró JM, et al. Comparative mortality of dominant Staphylococcus aureus lineages in human bacteremia and animal infection models. Microb Pathog. 2026 Apr;213:108323. doi:10.1016/j.micpath.2026.108323](https://ad-id.co/404gUlI)
  - Summary: This study demonstrates that Staphylococcus aureus virulence is lineage- and context-dependent, with ST398 showing high larval virulence linked to α- and δ-hemolysin production, CC30 showing reduced infectivity, and agrC disruption attenuating virulence in Galleria while enhancing vegetation formation in rabbits, highlighting the interplay between quorum sensing, hemolysins, and host-specific pathogenicity across infection models.
- [DeMeules MM, Proll SC, Hua X, Srinivasan S, Loeffelholz T, Liu C, et al. Gut microbiota and intestinal monodomination as a predictor for bacteremia in allogeneic hematopoietic cell transplant recipients. J Infect Dis. 2026 Feb 24; doi:10.1093/infdis/jiag005](https://ad-id.co/4cUPCFK)
  - Summary: In allogeneic hematopoietic cell transplantation recipients, gut microbiota disruption is common, and intestinal domination by a single species shows low positive predictive value for bacteremia, though high gut abundance of coagulase-negative Staphylococcus is associated with increased risk of corresponding bloodstream infections, suggesting the gut as a potential source for these pathogens.
- [Varisco B, Piovani D, Del Turco ER, Bussini L, Paul M, Sabik EF, et al. Clinical utility of the DENOVA score for predicting infective endocarditis in Enterococcus faecalis bacteremia: external validation with decision curve analysis. Clin Microbiol Infect. 2026 Feb 24; doi:10.1016/j.cmi.2026.02.019](https://ad-id.co/4sgAOWm)
  - Summary: The DENOVA score was externally validated in a large international cohort of 543 patients with Enterococcus faecalis bacteremia, showing good discrimination (AUC 0.871) and balanced sensitivity (79.2%) and specificity (83.0%) at the threshold ≥3, supporting its use for risk stratification and guiding echocardiographic evaluation to reduce unnecessary TEEs.

**Respiratory Tract Infections**

- [Cornia PB, Lipsky BA. Pertussis infection in adults. JAMA. 2026 Feb 18; doi:10.1001/jama.2025.26153](https://ad-id.co/3OVLOKx)
  - Summary: Pertussis (whooping cough) is a highly contagious respiratory infection caused by Bordetella pertussis, a human-restricted gram-negative coccobacillus; three other Bordetella species—B. parapertussis, B. bronchiseptica, and B. holmesii—can also cause respiratory illness in humans, with B. parapertussis producing clinically similar disease.
- [Turpin BB, Jones BE, Erstad BL, Wilson K. The 2025 CAP controversy: why guideline methodology matters for clinical decision-making. J Clin Pharm Ther. 2026 Feb 19; doi:10.1177/1060028026142223](https://ad-id.co/47gxYbS)
  - Summary: The 2025 community-acquired pneumonia guideline update emphasizes that clinical guidelines balance evidence, methodology, and value judgments; conditional recommendations reflect uncertainty, and understanding guideline rationale is essential for safe, evidence-informed bedside care.
- [Sweeney DA, Póvoa P, Kalil AC. Corticosteroids and community-acquired pneumonia: Africa deserves an explanatory trial. Crit Care. 2026;30:70.](https://ad-id.co/4soFn1b)
  - Summary: Community-acquired pneumonia in Africa differs significantly from high-resource settings, with younger patients, higher prevalence of HIV, tuberculosis, and vaccine-preventable pathogens. Corticosteroid therapy for CAP remains controversial, as prior trials have shown inconsistent mortality benefits. The SONIA trial in Kenya suggested a modest 3.4% absolute reduction in 30-day mortality with low-dose corticosteroids, but multiple biases—including open-label design, symptom-based pneumonia diagnosis, limited baseline data, and potential misclassification or confounding by co-infections—cast doubt on the generalizability and safety of corticosteroid use in this population.
- [Musher DM, Kulkarni PA, Rodriguez-Barradas MC. Commensal bacteria: an under-recognized cause of pneumonia. J Infect Dis. 2026 Feb 6; doi:10.1093/infdis/jiag053](https://ad-id.co/4snYvMS)
  - Summary: Many cases of community-acquired pneumonia lack an identified causative pathogen, and evidence suggests that “normal respiratory flora” or commensal bacteria may account for a substantial proportion of these infections.

**Urinary Tract Infections**

- [Cantón R, Lim A-N, Cortés V, Díaz-Regañón J. Uncomplicated urinary tract infections: from an invisible impact to a visible change in complex care. Clin Infect Dis. 2026 Feb 25; doi:10.1093/cid/ciaf705](https://ad-id.co/4se9B6L)
  - Summary: Uncomplicated urinary tract infections impose substantial physical and emotional burden, with frequent recurrences, and rising global antimicrobial resistance—particularly in Escherichia coli—necessitates careful consideration of drug, pathogen, and patient factors when selecting therapy.
- [Howard A, Green PL, Zhong Y, Hughes DM, Gerada A, Maskell S, et al. Algorithmic antibiotic decision-making in urinary tract infection using prescriber-informed prediction of treatment utility. npj Digit Med. 2026;9:136. doi:10.1038/s41746-026-02369-z](https://ad-id.co/3N70G8p)
  - Summary: An antibiotic decision-making algorithm integrating clinical prediction models with clinician judgment improved prescribing by selecting more appropriately targeted and oral WHO Access antibiotics, reducing intravenous use, and demonstrating potential to enhance antibiotic stewardship.

**Infections in Neonates and Children**

- [Puopolo KM. Group B streptococcal disease. N Engl J Med. 2026 Feb 25;394:896-905.](https://ad-id.co/4l3u6Rb)
  - Summary: Group B streptococcus is a leading cause of invasive neonatal infection in the United States; intrapartum antibiotics reduce early-onset disease, but vaccines combining capsular polysaccharides with protein antigens are in development to prevent disease in later infancy and nonpregnant adults.
- [Barshak MB, Linder JA, Watson ME Jr, Wessels MR, Carter DM, Cohen AL, et al. Overview of 2025 clinical practice guideline update by the Infectious Diseases Society of America on Group A streptococcal (GAS) pharyngitis: risk assessment using clinical scoring systems in children and adults. Clin Infect Dis. 2026 Feb 14; doi:10.1093/cid/ciag098](https://ad-id.co/4sdHMvj)
  - Summary: Clinical scoring systems for group A streptococcal pharyngitis modestly improve diagnostic accuracy over clinician judgment alone, and the IDSA panel recommends using such scores to guide testing with rapid antigen, molecular methods, or throat culture.

**Skin and Soft Tissue Infections**

- [Edslev SM, Liu CM, Lo BZS, Meiniche H, Lilje B, Park DE, et al. Changes in nasal and throat microbiota composition during and after mupirocin and chlorhexidine decolonisation treatment in asymptomatic MRSA carriers: a longitudinal observational study. Clin Microbiol Infect. 2026 Feb 24; doi:10.1016/j.cmi.2026.02.020](https://ad-id.co/3OTagMy)
  - Summary: Decolonisation of asymptomatic MRSA carriers with mupirocin and chlorhexidine rapidly alters the nasal microbiota, causing short-term and persistent shifts, while throat microbiota changes are transient; promoting recovery of beneficial commensals like Dolosigranulum may enhance post-treatment microbiome restoration.
- [Hooban B, Whelan SO, Burke A, Lucey M, Tumeo A, Mulrooney C, et al. Emerging extraintestinal Vibrio infections in Ireland from clinical and marine sources, 2020-2022. Clin Microbiol Infect. 2026 Feb 18; doi:10.1016/j.cmi.2026.02.013](https://ad-id.co/4l8GcJa)
  - Summary: This study reports the emergence of extraintestinal Vibrio infections in Ireland between 2020 and 2022 from both clinical and marine sources, highlighting the importance of public health surveillance in detecting and monitoring these infections, with isolates collected under routine surveillance, fully anonymized, and analyzed to inform infection trends and potential environmental links.

**Mycobacterial Infections**

- [Steyn J, Williams J, Naufal F, Limberis J, Grobbelaar M, Ismail N, et al. Bedaquiline resistance in patients with Xpert MTB/RIF Ultra-tested rifampicin-resistant tuberculosis in the Western Cape, South Africa: a prospective study. Lancet Microbe. 2026 Feb 19; doi:10.1016/j.lanmic.2025.101293](https://ad-id.co/4saz0OE)
  - Summary: This prospective study in the Western Cape, South Africa, evaluated bedaquiline resistance among patients with rifampicin-resistant tuberculosis using the Deeplex Myc-TB assay on 401 sequenced isolates, finding bedaquiline resistance in 12% of baseline and 41% of longitudinal isolates, and demonstrating that Deeplex testing of both resistance-associated and uncharacterized mmpR5 variants achieved high diagnostic accuracy with 93% sensitivity and 99% specificity when compared to a composite genotypic–phenotypic reference standard.
- [Adolph C, Mendelsohn SC, Via LE, Martinez L, Lindestam Arlehamn CS, Lewinsohn DM, et al. Global biological sample collections from tuberculosis studies: a scoping review. Lancet Microbe. 2026 Feb;7:1287. doi:10.1016/j.lanmic.2025.101287](https://ad-id.co/3N7sFEW)
  - Summary: A scoping review of tuberculosis observational studies and vaccine trials from 2014–2024 identified 104 observational studies and 18 vaccine trials across 43 countries, collectively collecting biological samples from over 35,000 tuberculosis cases, 39,000 contacts or controls, and 45,000 trial participants, with blood, genomic DNA, RNA, and sputum most commonly stored, and investigators expressing high interest in creating interconnected biorepositories, which could enable large-scale investigations, improve understanding of protective immunity, and accelerate tuberculosis vaccine development.
  - **Editorial Commentary:**[Adolph C, Mendelsohn SC, Via LE, Martinez L, Lindestam Arlehamn CS, Lewinsohn DM, et al. The need for interconnected global biorepositories from tuberculosis studies to address fundamental questions at scale. Lancet Microbe. 2026 Feb;7:1279. doi:10.1016/j.lanmic.2025.101279](https://ad-id.co/4sinErY)
- [Chen X, Ruiz-Gonzalez CE, Masias-Leon Y, Singh M, Peloquin CA, Jain SK. Addition of clofazimine enhances the activity of standard treatment regimen in a mouse model of tuberculous meningitis. J Infect Dis. 2026 Feb 23; doi:10.1093/infdis/jiag123](https://ad-id.co/4skVsox)
  - Summary: In a mouse model of tuberculous meningitis, adjunctive clofazimine added to the standard first-line tuberculosis regimen significantly reduced brain bacterial burden and attenuated neuroinflammation despite minimal cerebrospinal fluid penetration, suggesting that clofazimine enhances bactericidal activity and mitigates inflammation, supporting further investigation for clinical use in tuberculous meningitis.
- [Friedland JS. Physiology, host-directed therapies and personalised medicine for tuberculosis. Clin Microbiol Infect. 2026 Feb 18; doi:10.1016/j.cmicom.2026.105180](https://ad-id.co/404lnoo)
  - Summary: Tuberculosis remains a leading cause of infectious mortality globally, with treatment complicated by drug resistance and variable host immune responses, and while host-directed therapies (HDTs) such as corticosteroids, PAS, aspirin, and mTOR inhibitors show potential, their effectiveness is often limited by patient-specific physiological changes including metabolic alterations, hypoxia, acidosis, diabetes, fever, and lesion heterogeneity, highlighting the need for personalized HDT strategies that account for these factors to improve outcomes and reduce tissue damage in TB patients.
- [Howell P, Stillo J, Reuter A, Nkomo T, Mitnick CD, Guglielmetti L, et al. The looming crisis of bedaquiline-resistant tuberculosis and a promising way forward. Lancet Infect Dis. 2026 Feb 16; doi:10.1016/S1473-3099(26)00003-4](https://ad-id.co/4sj1wxB)
  - Summary: Drug-resistant tuberculosis is becoming increasingly dangerous as resistance to newer treatments like bedaquiline emerges, resulting in poor outcomes and high mortality in high-burden settings, while promising drugs remain years from regulatory approval, prompting a call for compassionate-use support platforms (CUSPs) to provide coordinated, equitable pre-approval access, improve safety monitoring, and ensure that patients with the most difficult-to-treat tuberculosis benefit from scientific advances.
- [Tirlangi PK, Pothumarthy VSK, Khan AR, Chellapuram SK, Grobusch MP, Gupta N. Mycobacterial infections in patients with hairy cell leukemia: a systematic review of published cases. Open Forum Infect Dis. 2026 Feb 11;13(2):ofag063. doi:10.1093/ofid/ofag063](https://ad-id.co/3OTwbTP)
  - Summary: Hairy cell leukemia (HCL) patients are highly susceptible to severe mycobacterial infections, which are often disseminated and involve multiple organs such as lymph nodes, lungs, liver, spleen, and bone marrow, with infections caused predominantly by nontuberculous mycobacteria like M. kansasii and M. avium complex, and carry a high mortality rate—particularly with pulmonary involvement—highlighting the need for prompt species-level diagnosis, vigilant multisystem evaluation, and early management in this immunocompromised population.
- [Kim M, Abu Saleh OM, Castillo Almeida NE, Wengenack NL, Deml SM, Comba IY. Clinical presentation, management, and outcomes of Mycobacterium bovis bacillus Calmette-Guérin (BCG) infections: a single-center retrospective review. Open Forum Infect Dis. 2026 Feb 18; doi:10.1093/ofid/ofag071](https://ad-id.co/3P9uq52)
  - Summary: Intravesical Mycobacterium bovis BCG therapy for high-risk non-muscle invasive bladder cancer can lead to localized, bloodstream-limited, or organ-disseminated infections, often presenting months to years after treatment and complicated by diagnostic delays due to limited sensitivity of standard tests, but despite prolonged therapy exceeding nine months, outcomes are generally favorable with a 94.7% cure rate and low attributable mortality, emphasizing the importance of early clinical suspicion, comprehensive diagnostics, individualized treatment, and surgical source control when needed.
- [Apriani L, Sidhu H, Korobitsyn A, Ismail N, Menzies D. Diagnostic performance of new commercial IGRAs for Mycobacterium tuberculosis infection: an updated systematic review and meta-analysis. Clin Infect Dis. 2026 Feb 25; doi:10.1093/cid/ciag102](https://ad-id.co/47hRiFE)
  - Summary: An updated systematic review and meta-analysis of eight newly commercialized Interferon Gamma Release Assays (IGRAs) compared to WHO-endorsed tests found that two new assays demonstrated promising accuracy and high agreement, though overall evidence certainty is low due to bias and imprecision, highlighting the need for high-quality studies in diverse populations to inform future WHO recommendations for tuberculosis infection testing.
- [Chen R, Shen Y, Liu H, Wang M, Fei Z, Xia L, et al. Intrathecal isoniazid and dexamethasone therapy may improve outcomes in patients with tuberculous meningitis: a two-center retrospective cohort study. Clin Infect Dis. 2026 Feb 18; doi:10.1093/cid/ciag106](https://ad-id.co/404mCnC)
  - Summary: A retrospective study of 533 tuberculous meningitis patients found that intrathecal administration of isoniazid and dexamethasone was associated with significantly better functional outcomes at discharge, suggesting that IT therapy may help overcome the blood–brain barrier and improve prognosis, though multicenter randomized trials are needed to confirm these findings.
- [Mulenga H, Mendelsohn SC, Fiore-Gartland A, Penn-Nicholson A, Musvosvi M, Tameris M, et al. Risk factors for immunological sensitization to Mycobacterium tuberculosis and progression to incident TB disease among HIV-uninfected adults in a high burden setting. J Infect Dis. 2026 Feb 18; doi:10.1093/infdis/jiag100](https://ad-id.co/404mCE8)
  - Summary: A prospective cohort study of 2,912 adults in high-TB-incidence South African sites found that 63.4% were Mtb-sensitized, and Mtb-sensitized individuals had a three-fold higher risk of prevalent TB and progression to active disease, with risk factors including increasing age, male sex, smoking, prior TB, TB contact, and lower BMI, highlighting the importance of targeted prevention strategies in high-prevalence settings.
- [Mutoh Y, Minato Y, Kawamoto Y, Hanai S, Umemura T, Suzuki H, et al. Diagnostic utility of quantitative interferon-gamma release assays in elderly patients with tuberculosis. Microbiol Spectr. 2026 Feb 10; doi:10.1128/spectrum.02763-25](https://ad-id.co/404mCUE)
  - Summary: A large retrospective study of 10,745 elderly Japanese patients found that quantitative interferon-gamma release assays (QFT-Plus and T-SPOT) have limited ability to distinguish active tuberculosis from latent infection, though discriminatory performance modestly improves when analysis is restricted to IGRA-positive individuals, suggesting that while quantitative IGRA values are not suitable as standalone diagnostics in the elderly, they may help guide risk stratification and clinical decision-making in selected cases.
- [Chen X, Ruiz-Gonzalez CE, Masias-Leon Y, Singh M, Nino-Meza OJ, Peloquin CA, et al. Beyond rifampin: evaluating rifapentine and rifabutin as alternative treatments for TB meningitis. J Infect Dis. 2026 Feb 10; doi:10.1093/infdis/jiag087](https://ad-id.co/4l3Bg8g)
  - Summary: In a mouse model of tuberculous meningitis, rifapentine- and rifabutin-based regimens demonstrated bactericidal activity in the brain comparable to or exceeding standard rifampin therapy, while also reducing neuroinflammation and neuronal injury, suggesting that these alternative rifamycins have both therapeutic and neuroprotective potential and warrant evaluation in clinical trials for TB meningitis.
- [Nieto Ramirez LM, Shelton K, Belisle JT, Diaz G, Tameris M, Naidoo K, et al. Optimized mass spectrometry to uncover M. tuberculosis biomarkers in extracellular vesicles from asymptomatic tuberculosis patients. J Infect Dis. 2026 Feb 10; doi:10.1093/infdis/jiag086](https://ad-id.co/404mDba)
  - Summary: Using quantitative mass spectrometry of serum-derived extracellular vesicles, researchers identified 19 Mycobacterium tuberculosis proteins and peptides that were differentially abundant in individuals with early or asymptomatic TB, including Rv2997, HspX, GroEL2, GroES, and a MtrB peptide, suggesting their potential as biomarkers for detecting subclinical TB and providing insights into host–pathogen interactions during early infection.

**Fungal Infections and antifungal agents**

- [Murtagh M, White PL, Rodriguez-Tudela JL, Alastruey-Izquierdo A, Chen SCA, Dufresne PJ, et al. Global perspective on gaps in fungal diagnostics in low-resource settings: WHO landscape analysis and research priorities for invasive fungal diseases. Lancet Microbe. 2026 Feb 23; doi:10.1016/j.lanmic.2025.101307](https://ad-id.co/404mDrG)
  - Summary: WHO’s 2024 diagnostic landscape analysis highlights that invasive fungal diseases disproportionately burden low- and middle-income countries where diagnostics remain largely culture-dependent, infrastructure-intensive, and inaccessible, with limited availability of affordable non-culture-based and multiplex platforms for broad pathogen and antifungal resistance detection, underscoring the urgent need for simplified, rapid, and cost-effective diagnostic innovations to reduce IFD-related morbidity and mortality.
- [Rhodes J, Hui ST, Dellière S, Summerbell RC, Scott JA, Kaur A, et al. Emerging terbinafine-resistant Trichophyton indotineae between 2018 and 2023: a multinational genomic epidemiology study. Lancet Microbe. 2026 Jan 22;7:1273. doi:10.1016/j.lanmic.2025.101273](https://ad-id.co/404mDIc)
  - Summary: A genomic epidemiology study of severe dermatophytosis cases across multiple countries confirmed rapid transcontinental spread of terbinafine-resistant Trichophyton indotineae, with 70% of isolates resistant and largely sharing a single evolutionary origin marked by SQLE mutations—though some resistant isolates lacked these mutations—highlighting the urgent need for enhanced genomic surveillance to manage this emerging global dermatophyte threat.
- [Garcia-Bustos V, Puchades F, Alonso-Ecenarro F, Cabanero-Navalon MD, Ruiz-Gaitán A, Pemán J, et al. Development and validation of the AURIS score for predicting candidaemia in Candidozyma auris-colonised patients in the intensive care unit: a bicentric retrospective cohort study. Lancet Infect Dis. 2026 Feb 18; doi:10.1016/S1473-3099(26)00002-2](https://ad-id.co/404mEvK)
  - Summary: In a bicentric ICU cohort in Spain, a refined four-variable AURIS score (including total parenteral nutrition, prior antifungal therapy, multifocal colonisation, and urinary isolation) demonstrated good discrimination (AUC 0.81) and outperformed the Candida score in predicting candidaemia among Candidozyma auris-colonised patients, offering a practical tool to identify low-risk individuals and reduce unnecessary empirical antifungal use, pending broader validation.
- [Herbel S, Paccoud O, de Montmollin É, Grenier B, Picard M, Vigneron C, et al. Presentation and prognosis of cryptococcosis requiring intensive care unit admission in France: the CRYPTO-ICU study. Clin Infect Dis. 2026 Feb 18; doi:10.1093/cid/ciag091](https://ad-id.co/404mEMg)
  - Summary: In a multicenter French ICU study of 151 severe cryptococcosis cases, nearly half died by 90 days—with mortality strongly associated with higher SOFA scores, disseminated infection, earlier admission period, and need for organ support, but not HIV status—highlighting a growing burden among non-HIV immunosuppressed patients and the critical importance of early diagnosis and antifungal therapy.
- [Mitaka H, Barron MA. Peritoneal Coccidioidomycosis. N Engl J Med. 2026 Feb 18;394:793.](https://ad-id.co/404mFQk)
  - Summary: A 23-year-old previously healthy man who recently moved to Arizona presented with 2 months of weight loss, abdominal pain, and distention; imaging revealed diffuse peritoneal nodularity, ascites, lymphadenopathy, and pleural effusion. Laparoscopy and biopsy demonstrated necrotizing granulomas with large spherules and endospores, and peritoneal tissue culture grew *Coccidioides*, confirming peritoneal coccidioidomycosis. HIV testing was negative and lumbar puncture ruled out meningitis. The patient was treated with prolonged fluconazole, leading to symptom resolution at 1-month follow-up.
- [Maguire CA, Orlow SJ. Majocchi’s Granuloma. N Engl J Med. 2026 Feb 14;394:e13. doi:10.1056/NEJMicm2509457](https://ad-id.co/404mG6Q)
  - Summary: A 17-year-old high-school wrestler developed a 6-week itchy rash on his calf that worsened after treatment with a topical antifungal and high-potency glucocorticoid. Examination revealed a 6 cm circular red, scaly plaque with papules, nodules, and pustules. Skin scraping culture grew *Trichophyton tonsurans*, and a clinical diagnosis of Majocchi’s granuloma—a deep follicular dermatophyte infection often triggered by topical steroids—was made. The patient was treated with oral terbinafine, leading to resolution after 1 month and sustained improvement at 6 months post-treatment.
- [Mezzogori L, Bavastro M, Magnasco L, Centorrino F, Schiavoni R, Portunato F, et al. Testing to detect Candida auris colonisation after intrahospital transfer from an endemic area: a prospective observational study. Mycoses. 2026 Feb;69(2):e70138. doi:10.1111/myc.70138](https://ad-id.co/404mGnm)
  - Summary: In a prospective study of 462 adults transferred from a *Candida auris* endemic ICU to non-endemic wards, 440 were initially non-colonised. Among 208 patients sufficiently screened with ≥2 swabs in the first 4 weeks post-transfer, 16.3% were newly colonised, most detected on the first post-transfer swab. A negative swab within 24 hours before transfer missed 7.1% of later-positive cases. Post-transfer colonisation was associated with candidemia (11.8%), whereas non-colonised patients did not develop infection. The study concludes that a single negative swab at ICU discharge is insufficient; repeated screening within the first 2 weeks post-transfer is critical to detect colonisation and prevent *C. auris* transmission.
- [Chen S, Wei Y, Wang Q, Li Y, Pei F, Liu W, et al. Antifungal susceptibility surveillance of clinical moulds to olorofim, manogepix, amphotericin B, triazoles and echinocandins at 10 tertiary hospitals in China (2019–24). J Antimicrob Chemother. 2026 Mar;81(3):dkag016. doi:10.1093/jac/dkag016](https://ad-id.co/404mGDS)
  - Summary: A multicenter study in China (2019–2024) analyzed clinical mould isolates, finding Aspergillus spp. most prevalent (92.0%), followed by Fusarium spp. (4.2%) and Mucorales (1.9%), with non-*A. fumigatus* species increasing. Triazole resistance in Aspergillus was uncommon (2.5%) but linked to cyp51A or hmg1 mutations. Novel antifungals olorofim and manogepix showed potent in vitro activity against most moulds, including triazole-resistant Aspergillus, whereas Mucorales were less susceptible. These findings highlight emerging non-*A. fumigatus* prevalence, low triazole resistance, and broad-spectrum potential of new antifungal agents.

**Virulence**

- [Fong W, Rockett RJ, Tam KK-G, Nguyen T, Sim EM, Tay E, et al. Characterisation of Bordetella pertussis virulence and macrolide resistance in Australia by targeted culture-independent sequencing: a genomic epidemiology study. Lancet Microbe. 2026 Feb 25;7:101286. doi:10.1016/j.lanmic.2025.101286](https://ad-id.co/4snYwjU)
  - Summary: A nationwide Australian study applied targeted culture-independent next-generation sequencing (tNGS) to 255 *Bordetella pertussis*–positive respiratory specimens, recovering near-complete genomes from 58%. Seven co-circulating lineages were identified, including two with macrolide resistance. Eight cases of macrolide-resistant *B. pertussis* carrying the 23S rRNA 2037A→G mutation were detected, with an estimated 4% prevalence among positive cases. tNGS enables direct detection of resistance mutations, high-resolution phylogenetics, and complements PCR surveillance for genomic and epidemiologic insights.
- [Gallagher KE, Odiwour F, Bottomley C, Ojal J, Adamu A, Muthumbi E, et al. Serotype-specific pneumococcal invasiveness: a global meta-analysis of paired estimates of disease incidence and carriage prevalence. Lancet Microbe. 2026 Feb 23;7:101301. doi:10.1016/j.lanmic.2025.101301](https://ad-id.co/4smNPOw)
  - Summary: A systematic review of 80 studies from 18 countries calculated serotype-specific case–carrier ratios (CCRs) for pneumococcal disease across income settings, age groups, HIV status, and pre- versus post-PCV introduction. In children under 5, non-PCV13 serotypes had higher pre-PCV CCRs in low- and lower-middle-income countries (177) than in upper-middle- and high-income countries (103). Post-PCV, CCRs declined in high-income settings (26) but remained high in low-income settings (173). CCRs were lowest in 5–14-year-olds and higher in HIV-positive individuals. These estimates provide representative invasiveness data for modeling pneumococcal disease incidence using carriage data.
- [Gibbon MJ, Couto N, Cozens K, Habib S, Cowley L, Aanensen DM, et al. Convergence and global molecular epidemiology of Klebsiella pneumoniae plasmids harbouring the iuc3 virulence locus: a population genomic analysis. Lancet Microbe. 2026 Feb;7:101236. doi:10.1016/j.lanmic.2025.101236](https://ad-id.co/47hRiWa)
  - Summary: A global genomic analysis of 517 iuc3-carrying plasmids from 4148 Klebsiella isolates revealed widespread diversity across ecological and geographical sources. Most iuc3 plasmids were found in pigs or pork, but a subset was present in clinical isolates. Plasmid hybridisation between iuc3 virulence plasmids and antimicrobial resistance plasmids, including ESBLs, was observed, producing convergent plasmids in hospital and market settings in Asia. Phylogenetic clustering identified three plasmid groups: group 1 primarily in European pigs, group 2 heterogeneous across sources, and group 3 associated with Asian clinical isolates carrying multiple ARGs and virulence factors. These findings highlight the role of environmental reservoirs in the emergence of virulent, resistant Klebsiella and underscore the need for targeted plasmid surveillance.
- [Edirmanasinghe R, Dingle TC, Croxen MA, Boyd DA, Mataseje L. Carbapenemase-producing hypervirulent Klebsiella pneumoniae in Alberta, Canada, 2016–2024. J Assoc Med Microbiol Infect Dis Can. 2026 Feb 11; doi:10.3138/jammi-2025-0028](https://ad-id.co/400sCxE)
  - Summary: A genomic surveillance study in Alberta, Canada identified 16 carbapenemase-producing Klebsiella pneumoniae isolates, spanning eight sequence types, including globally recognized high-risk lineages (ST11, ST15, ST231, ST147, ST395) and, for the first time in Canada, the hypervirulent ST23 lineage. Carbapenemase genes detected included blaNDM-1, blaOXA-48-type, and combinations thereof. The ST23 isolate carried chromosomal hypervirulence genes (ybt, clb) and a 229.9 kb virulence plasmid encoding iro and iuc, alongside a 92.3 kb plasmid with blaOXA-48. Nearly half of isolates carried both virulence determinants and carbapenemase genes on the same plasmid, forming hybrid plasmids. Despite this, 81% were negative on the hypermucoviscous string test, highlighting the cryptic nature of hypervirulence in multidrug-resistant strains. These findings underscore the emergence of hypervirulent, carbapenem-resistant K. pneumoniae in Canada and the need for genomic surveillance to detect such high-risk strains.
- [Xu L, Li J, Wu W, Dou L, Wang J, Qiu M, et al. Decoding hypervirulence in carbapenem-resistant Klebsiella pneumoniae: genomic and phenotypic profiling reveals capsular polysaccharide as a key driver of pathogenicity. J Infect Dis. 2026 Feb 9; doi:10.1093/infdis/jiag075](https://ad-id.co/3NcjYcv)
  - Summary: A study of 59 carbapenem-resistant Klebsiella pneumoniae (CRKP) isolates using a mouse subcutaneous model identified 37% as hypervirulent (hv-CRKP). Patients infected with hv-CRKP experienced higher sepsis rates and mortality. Capsule production and hypermucoviscosity reliably distinguished hv-CRKP from CRKP, while genome-wide association and transcriptomic analyses highlighted chromosomal factors—particularly rcsA-mediated capsule upregulation—as key contributors to hypervirulence by enhancing resistance to macrophage phagocytosis. Multivariable and LASSO regression confirmed capsule production and rcsA expression as robust biomarkers for hv-CRKP detection. These findings underscore the need for validated biomarkers to accurately identify clinically significant hypervirulent CRKP strains.

**Host Factors in Infection**

- [Damhorst GL, Boulis MJ, Nelson EB, Hofstetter KS, Kim J, Sakurai Y, et al. Variable staphylothrombin activity in Staphylococcus aureus bloodstream infections. J Infect Dis. 2026 Feb 13; doi:10.1093/infdis/jiag012](https://ad-id.co/401fOXV)
  - Summary: A high-throughput assay was developed to measure Staphylococcus aureus staphylothrombin activity and growth fitness by exposing human plasma to bacterial culture supernatants. Results showed marked variability in coagulase-driven fibrin formation across isolates and plasma donors, indicating that host–pathogen compatibility influences observed phenotypes. Analysis of transposon mutants, plasma coagulation factors, and thrombin inhibition suggested complex determinants of coagulase-mediated virulence. These findings highlight the potential for using staphylothrombin activity profiles to inform novel therapeutic strategies and risk stratification in S aureus bloodstream infections.
- [Amar N, Chakroun A, Ben-Taleb H, Sebbah H, Lamrissi A, Badre A, et al. Inflammatory cytokine profile in pregnant women colonized with Group B Streptococcus reveals IL-17a as a potential biomarker to identify at-risk newborns. J Infect Dis. 2026 Feb 11; doi:10.1093/infdis/jiaga092](https://ad-id.co/3N70GoV)
  - Summary: In a prospective cohort study in Morocco, GBS-colonized pregnant women whose newborns developed invasive GBS disease showed significantly lower maternal IL-1β, IL-4, and IL-17A levels compared with those whose newborns remained healthy. Ex vivo stimulation of maternal blood with TLR4 and TLR1/2 ligands confirmed reduced cytokine responses. Maternal IL-17A emerged as a strong predictive biomarker for neonatal GBS transmission and invasive disease, highlighting its potential for risk stratification in GBS-colonized mother–newborn dyads.

**Diagnostics**

- [De Swardt H, Bernabeu S, Dortet L, Emeraud C. Evaluation of the Carbapenemase Detection Kit (Colloidal Gold; Macro & Micro Test): a new immunochromatographic assay for rapid detection of carbapenemases in Enterobacterales. J Antimicrob Chemother. 2026 Mar;81(3):dkag066. doi:10.1093/jac/dkag066](https://ad-id.co/4saz15a)
  - Summary: This study evaluated the performance of the M&M Carba Test, a lateral-flow immunochromatographic assay for rapid detection of carbapenemase-producing Enterobacterales (CPE), using a collection of 242 well-characterized isolates from France. The panel included 194 carbapenemase producers (188 with target enzymes such as KPC, NDM, VIM, and OXA-48-like, and six with non-targeted enzymes) and 48 non-producers. Whole-genome sequencing was used to confirm the carbapenemase profiles, ensuring a representative assessment of the assay’s diagnostic accuracy against both common and emerging variants.
- [Chilleri C, Salvetti S, Coppi M, Montenora I, Giani T, Rossolini GM, Antonelli A. Evaluation of the Performance of Novel Gram-Negative and Gram-Positive Sepsis Panels for the Rapid Diagnosis of Bloodstream Infections. Diagnostics. 2026;16(3):481. doi:10.3390/diagnostics16030481](https://ad-id.co/404cbk0)
  - Summary: This study evaluated the Molecular Mouse (MM) Sepsis panels for rapid identification (ID) of Gram-negative and Gram-positive bacteria and detection of resistance determinants (RDs) directly from positive blood cultures. Among 140–136 valid samples, species-level ID agreement with standard-of-care (SoC) methods was 89% overall (GN 89%, GP 92%), while RD detection showed 99% agreement. The MM panels demonstrated high sensitivity for RDs, though some species-specific ID discrepancies occurred. Their compact size and modular design make them a practical tool for rapid bloodstream infection diagnostics.
- [Davenport CF, Rutjes AWS, Mallett S, Tomlinson E, Yang B, Holmes J, Westwood ME, Takwoingi Y, Reitsma JB, Hyde C, Bossuyt PMM, Deeks JJ, Leeflang MMG, Whiting PF. QUADAS-3 Explanation and Elaboration: Guidance for Quality Assessment of Diagnostic Test Accuracy Studies. Ann Intern Med. 2026; published online February 17, 2026. doi:10.7326/ANNALS-25-04943](https://ad-id.co/47iI9wB)
  - Summary: QUADAS-3 is the latest version of the QUADAS tool, designed to assess risk of bias and applicability in diagnostic test accuracy studies. This article provides guidance on defining review questions, conceptualizing ideal trials, interpreting domains and signaling questions, and making judgments about bias and applicability, serving as a practical guide for systematic reviewers.
  - **Editorial Commentary:**[Whiting PF, Tomlinson E, Rutjes AWS, Davenport CF, Yang B, Westwood ME, Takwoingi Y, Mallett S. QUADAS-3: A Revised Tool for the Quality Assessment of Diagnostic Test Accuracy Studies. Ann Intern Med. 2026; published online February 17, 2026. doi:10.7326/ANNALS-25-02104](https://ad-id.co/4spbhL3)
- [Olearo F, Last K, Leibovici L. Revisiting Diagnostics: Progress, Gaps, and the Road Ahead. Clin Microbiol Infect. 2026; published online February 13, 2026. doi:10.1016/j.cmi.2026.02.009](https://ad-id.co/4sdHMLP)
  - Summary: The editorial “Revisiting Diagnostics: Progress, Gaps, and the Road Ahead” (Olearo, Last, Leibovici, 2026) introduces a series reflecting on diagnostic practices in clinical microbiology, highlighting the need to evaluate which methods should be adopted, optimized, or potentially discontinued.

**Improving Clinical Research**

- [Gregson J, Redfors B, Cohen DJ, Pinto D, Fahy M, Pocock S. Evaluating Noninferiority in Clinical Trials with a Hierarchical End Point. NEJM Evid. 2026;5(3). doi:10.1056/EVIDctw2500029](https://ad-id.co/4sDP2Bd)
  - Summary: The article explains how the win ratio, typically used in superiority trials, can be applied to noninferiority trials by selecting an appropriate noninferiority margin and translating it into familiar metrics, providing a practical framework illustrated with a hypothetical example.
- [Glazier A, DeCormier Plosky W, Bierer BE. Disclosure of Pregnancy-Related Privacy Risks in Clinical Research Post-Dobbs. NEJM Evid. 2026;5(3). doi:10.1056/EVIDra2500239](https://ad-id.co/3ZZRO7v)
  - Summary: This review examines how routine reproductive protections in clinical research—such as pregnancy testing and contraception—pose new risks to participants and investigators following the Dobbs v. Jackson Women’s Health Organization decision, offering practical guidance for researchers and IRBs navigating these legal challenges.
- [Yeretsian T, Viau-Lapointe J, Ashraf R, D'Souza R, Lapinsky SE. A core outcome set and reporting checklist for research on critically ill obstetric patients: An international consensus study. Acta Obstet Gynecol Scand. 2026. doi:10.1111/aogs.70161](https://ad-id.co/4sbcJAu)
  - Summary: COSCO, a Core Outcome Set for research on critically ill obstetric patients, was developed through international consensus, identifying 10 core outcomes and 7 reporting items to standardize research, improve comparability, and support patient-centered, evidence-based guidelines.
- [Prasad V, Makary MA. One pivotal trial, the new default option for FDA approval — ending the two-trial dogma. N Engl J Med. 2026;394:815-817.](https://ad-id.co/4l63vTV)
  - Summary: Since 1997, the FDA has allowed marketing approvals based on a single adequate and well-controlled study plus confirmatory evidence, but two trials have historically been preferred. In 2026, the FDA announced that the default standard will now require only one robust trial, supported by confirmatory evidence, to reduce costs, accelerate drug development, and maintain credibility. Decisions will consider trial design, effect size, endpoints, controls, statistical rigor, and biological plausibility, rather than simply the number of trials. Two trials may still be required for interventions with uncertain mechanisms, surrogate outcomes, or other limitations. The change aims to stimulate biomedical innovation while preserving patient safety and regulatory standards.
- [Shu S, Hamasaki T, Evans S, Komarow L, van Duin D, Diao G. Doubly robust estimation of desirability of outcome ranking (DOOR) probability with application to MDRO studies. arXiv. 2026;arXiv:2602.10012. doi:10.48550/arXiv.2602.10012](https://ad-id.co/3OTwcal)
  - Summary: In observational studies, covariate adjustment is essential when comparing treatments to reduce bias and improve precision. The Desirability of Outcome Ranking (DOOR) method, originally developed for randomized trials, ranks patient outcomes from most to least desirable, integrating both benefits and risks. This work extends DOOR to observational studies using causal inference methods: inverse probability of treatment weighting, G-Computation, and a doubly robust approach combining both. Simulation studies evaluate these methods, and an applied analysis in the Antibacterial Resistant Leadership Group (ARLG) MDRO network compares benefit-risk between mono-drug and combination-drug therapies.
- [Lopes ECD, Bulgarelli L. Federated analysis for critical care: opportunities and challenges for research. Crit Care Sci. 2026;38:e20260263. doi:10.62675/2965-2774.20260263](https://ad-id.co/4aKR6RL)
  - Summary: Single-center studies and locally trained models often lack power and generalizability, motivating multi-institution collaborations. Traditional collaborations require patient-level data sharing, which is restricted by privacy regulations. Federated frameworks, including Federated Analysis (FA) and Federated Learning (FL), allow statistical analyses and model training across institutions without transferring patient-level data, sharing only aggregate statistics. These frameworks enhance reproducibility, enable exploration of population heterogeneity, and strengthen statistical power. Platforms like TriNetX, Datashield, EHDEN, and Critical Care Asia exemplify successful federated deployments for multicenter benchmarking and quality studies, including participation from low- and middle-income countries. In critical care, FL has supported generalizable AI tools for outcomes such as mortality, sepsis, and acute kidney injury. Nonetheless, challenges remain around heterogeneity, privacy, and data management.
- [Chu MHM, Wong HMK, Ho KM. Strategies to enhance the likelihood of detecting true effects in randomised controlled trials. J R Coll Physicians Edinb. 2026. Published online February 8, 2026. doi:10.1177/0310057X251366319](https://ad-id.co/4r0tiy9)
  - Summary: Randomized controlled trials (RCTs) are the gold standard for evidence-based medicine, but their high cost challenges researchers and funders. Trial efficiency has gained attention over the past decade, as failing to detect an effective treatment can delay patient access and discourage further research. The US FDA has issued guidance to improve the likelihood of detecting true effects, though trialists play a central role in enhancing the scientific efficiency of RCTs.
- [Henderickx JGE, Smits WK, Zeller GF, Kuijper EJ. How to set up and manage a microbiome research facility. Clin Microbiol Infect. 2026. Published online February 13, 2026. doi:10.1016/j.cmi.2026.02.007](https://ad-id.co/404lnEU)
  - Summary: The complexity of human microbiome research—spanning study design, data generation, bioinformatics, and statistical analysis—requires specialized expertise to avoid biases and errors. Drawing on the experience of the microbiome research facility at Leiden University Medical Center, this review highlights essential aspects of such a facility, including structure, financial and legal frameworks, and key services. Dedicated microbiome facilities centralize support for clinical studies, enable methodological innovation, foster industry collaborations, and strengthen funding applications, thereby advancing high-quality microbiome research and education.

**General Interest**

- [Kung MS, Crawford JR. Subacute sclerosing panencephalitis after measles infection. N Engl J Med. 2026;394:e14. Published February 21, 2026.](https://ad-id.co/4snYwAq)
  - Summary: A 7-year-old boy developed progressive cognitive decline and seizures three months before presentation, following measles infection at 7 months of age in Afghanistan. Examination revealed diffuse hyperreflexia and clonus; MRI showed frontal lobe edema and restricted diffusion, and EEG demonstrated bilateral high-amplitude periodic discharges (Radermecker complexes). Cerebrospinal fluid analysis revealed elevated total IgG and markedly increased measles-specific IgG. He was diagnosed with subacute sclerosing panencephalitis, a fatal neuroinflammatory disorder caused by persistent measles virus infection, and died 12 months after symptom onset. Vaccination remains the primary prevention.
- [Bendapudi PK, Klug M, Azar SS, Murali MR, Al Jurdi A. Case 6-2026: a 91-year-old man with shortness of breath, weight loss, and eosinophilia. N Engl J Med. 2026;394:794-805. Published February 18, 2026.](https://ad-id.co/4sj1wO7)
  - Summary: A 91-year-old man with a history of chronic obstructive pulmonary disease, coronary artery disease, and paroxysmal atrial fibrillation presented with progressively worsening dyspnea, weight loss, and marked eosinophilia over 8 months. He had multiple prior hospital visits for dyspnea and cough, with fluctuating pulmonary opacities on chest radiographs and elevated eosinophil counts ranging from 1,280 to 2,090/µL. On the current admission, oxygen saturation after ambulation was 70%, and laboratory evaluation revealed profound leukocytosis with an absolute eosinophil count of 27,390/µL. Imaging showed persistent and recurrent lung opacities with new small bilateral pleural effusions. Electrocardiography demonstrated atrial fibrillation, and biomarkers indicated cardiac stress with elevated NT-proBNP (4,300 pg/mL) and troponin T (56 ng/L). Peripheral-blood smear confirmed mature eosinophilia. The presentation suggested a recurrent or persistent eosinophilic process affecting the lungs and potentially the heart.
- [Giovannoni G, Payne O, Valero-Hernández E, Kang AS, Singh BK, Baker D, Harris K, Cutino-Moguel T, James LK, Bloom BM. Post-streptococcal autoimmunity and its relevance to Epstein–Barr virus as the potential cause of multiple sclerosis. J Infect Dis. 2026; jiag035. Published February 12, 2026. doi:10.1093/infdis/jiag035](https://ad-id.co/4sjHZgO)
  - Summary: Epstein–Barr virus (EBV) infection is strongly implicated as a necessary, but not sufficient, factor in the development of multiple sclerosis (MS), suggesting that additional host or environmental factors determine disease onset. Current immunologic models favor molecular mimicry to explain EBV’s role in triggering MS autoimmunity. By analogy, group A beta-hemolytic streptococcus (GAS) is a well-established infectious trigger of autoimmune conditions such as acute rheumatic fever, Sydenham’s chorea, glomerulonephritis, arthritis, and vasculitis. The authors hypothesize that EBV may similarly drive MS disease activity, akin to how GAS infections precipitate flares in ARF.
- [Wooten D, Hendrix C, Nordman J. More than a message: death by a 1000 “chats.” Clin Infect Dis. 2026; ciag094. Published February 14, 2026. doi:10.1093/cid/ciag094](https://ad-id.co/3OVLP13)
  - Summary: Secure electronic messaging within EHRs, such as Epic Chat, enhances rapid communication but can fragment attention, disrupt clinical reasoning, and increase medicolegal risk for Infectious Diseases consultants. Repetitive or trivial messages exacerbate these issues. To protect clinician well-being and optimize patient care, purposeful communication is essential, including setting clear boundaries, defining when to use chat versus phone or in-person communication, batching responses, protecting focus time, and teaching messaging etiquette.
- [de Gaay Fortman DPE, Kullberg RFJ, Wiersinga WJ, Haak BW. Dynamics of the gut and lung microbiota in severe infections: from observational studies to therapeutic strategies. Clin Microbiol Infect. 2026. Published online February 24, 2026. doi:10.1016/j.cmi.2026.02.016](https://ad-id.co/4r0tjlH)
  - Summary: Severe infections and sepsis disrupt gut and lung microbiomes, depleting obligate anaerobes and immunomodulatory metabolites, which impairs mucosal integrity, immune homeostasis, and increases risk of secondary infections and organ failure. Gut–lung crosstalk influences systemic and pulmonary immunity, with critical illness favoring overgrowth of opportunistic pathogens. Strategies to improve outcomes include antibiotic stewardship to preserve anaerobes and experimental interventions such as defined live anaerobic consortia or postbiotics to restore microbial balance.
- [Dumchev K, Mc Gann P, Danyliuk O, Metreveli M, Musich TA, Bennett JW. Microbial Flora in War Wounds from the Ukrainian Front Line. N Engl J Med. 2026;394:926-928. Published February 25, 2026.](https://ad-id.co/4kZX3NU)
  - Summary: Wounds of Ukrainian soldiers shortly after injury are primarily colonized by low-virulence commensal and environmental bacteria, with few antimicrobial-resistance genes; highly resistant pathogens likely arise later through nosocomial transmission. Early use of broad-spectrum antibiotics may promote resistance, highlighting the need to reconsider prophylaxis protocols and strengthen infection-control measures at hospitals and prehospital settings.

**Trial Protocols / Trial Ideas**

- [Pinot J, Delory T, Pétrier M, Aubin Auger I, Whiston M, Issa A, et al. Clinical data, chest X-ray or C-reactive protein to initiate antibiotic therapy in outpatients with suspected community-acquired pneumonia: a prospective, randomised, controlled, digital case vignette study. Clin Microbiol Infect. 2026 Mar 14; doi:10.1016/j.cmi.2026.03.010](https://ad-id.co/3NGAGkt)
  - Summary: This prospective, randomised digital case vignette study of 3,729 French general practitioners found that using chest X-ray (CXR) as a first-line test in patients with clinically suspected community-acquired pneumonia significantly reduced antibiotic initiation by 21% overall and up to 51% when CXR was negative, while C-reactive protein (CRP) testing reduced antibiotic use by 15%, and combining CRP with CXR influenced prescribing depending on CRP results, demonstrating that systematic CXR, and to a lesser extent CRP, can effectively decrease unnecessary antibiotic prescriptions in primary care.

**Antibiotic Therapy Reviews**

- [Crowley PD, Meagher KM, Barwise AK. Antibiotics in hospice: applying the four-quadrant approach to improve patient-centered care. Clin Infect Dis. 2026 Mar 5; doi:10.1093/cid/ciag057](https://ad-id.co/3NGAGRv)
  - Summary: This article discusses the complexities of antibiotic use in hospice care, highlighting how applying the four-quadrant approach—which considers medical indications, patient preferences, quality-of-life factors, and contextual features like antimicrobial resistance and cost—can guide individualized decisions, emphasizing that antibiotics may be appropriate for some hospice patients and that more evidence-based data is needed to inform treatment discussions at enrollment.

**PK/PD and Drug Dosing**

- [Farooq A, Martens M, Attwood MLG, Nordmann P, MacGowan A, Wicha SG. Meropenem and fosfomycin against K. pneumoniae: towards a combination breakpoint using a pharmacometric approach. Clin Microbiol Infect. 2026 Mar 6; doi:10.1016/j.cmi.2026.02.024](https://ad-id.co/3NGAHox)
  - Summary: This pharmacometric study of 12 multidrug-resistant Klebsiella pneumoniae strains demonstrates that while meropenem or fosfomycin monotherapy achieves high probability of target attainment (PTA) only at low MICs, their combination produces strong synergistic effects, enabling effective bacteriostasis and bacterial killing at much higher MICs, supporting the reintroduction of a fosfomycin breakpoint in combination therapy and providing an evidence-based rationale for optimized dosing regimens in severe infections.
- [Supparitsch S, Zeitlinger M. Experimental biofilm models for pharmacokinetic and pharmacodynamic investigations: bridging in vitro, ex vivo and in vivo systems. J Antimicrob Chemother. 2026 Apr; doi:10.1093/jac/dkag091](https://ad-id.co/4ly7iJM)
  - Summary: This review highlights the challenges of treating biofilm-associated infections due to reduced antimicrobial susceptibility and limited predictive value of conventional PK/PD indices, and provides a structured overview of in vitro, ex vivo, and in vivo experimental models—each with distinct advantages and limitations—emphasizing that combined use and standardization of these models can enhance translational relevance and guide preclinical antimicrobial research and development.

**Beta-Lactamases and Other Resistance Mechanisms**

- [Yang W, Tang C, Shi Q, Ou H, Yin D, Han R, et al. Ceftazidime-avibactam-resistant Klebsiella pneumoniae driven by multiple β-lactamase mutation: a hospital outbreak report. Clin Microbiol Infect. 2026 Mar 13; doi:10.1016/j.cmi.2026.03.014](https://ad-id.co/3N7HYO5)
  - Summary: This study reports a nosocomial clonal dissemination of six ceftazidime-avibactam-resistant Klebsiella pneumoniae strains in a hepatobiliary surgery ward and uncovers that resistance is mediated not by KPC-157 but by a novel CTX-M-249 enzyme, evolved from CTX-M-65 through specific mutations, which can transfer under selective antibiotic pressure and may coexist with CTX-M-65 on separate plasmids, highlighting the complex evolution of resistance and raising concerns for global infection prevention and control.

**Adverse Effects from Antimicrobial Agents**

- [Winner KM, Chanderraj R, Nuppnau M, He Y, Petouhoff AM, Falkowski NR, et al. Antianaerobic antibiotics, gut microbiota, and sepsis-associated acute kidney injury. Am J Respir Crit Care Med. 2026 Feb;212(2):314–326; doi:10.1164/rccm.202411-2281OC](https://ad-id.co/4rDO1Ih)
  - Summary: This study of 12,776 septic patients, supported by instrumental variable analyses, a gut microbiota case-control study, and a murine sepsis model, demonstrates that early use of antianaerobic antibiotics significantly increases the risk of sepsis-associated acute kidney injury (AKI) and delays its resolution, with gut microbiome alterations—particularly enrichment of Enterobacteriaceae and Lachnospiraceae—identified as a key mechanistic contributor to AKI susceptibility.
  - **Editorial Commentary:**[Lydon EC, Liu KD, Calfee CS. Are we overlooking a hidden organ in sepsis-associated AKI? Am J Respir Crit Care Med. 2026 Feb;212(2):283–285; doi:10.1093/ajrccm/aamaf098](https://ad-id.co/4rA81vr)

**Antibiotic Stewardship and Hospital in the Home**

- [Masucci L, Schwartz K, Ivers N, Bai L, Brown K, Tadrous M, et al. Mailed audit and feedback for antibiotic prescribing in primary care. JAMA Netw Open. 2026 Mar 13;9(3):e261641; doi:10.1001/jamanetworkopen.2026.1641](https://ad-id.co/3P9BuPq)
  - Summary: This economic evaluation of a randomized clinical trial involving 4,879 primary care physicians in Ontario, Canada, found that a low-cost mailed antibiotic audit and feedback program significantly reduced antibiotic prescribing in patients aged 65 and older and generated an estimated return of $8.82 CAD for every dollar invested, demonstrating that scalable A&F interventions can deliver substantial clinical and economic value as a high-impact antimicrobial stewardship strategy in primary care.
- [Mackow NA, Shao W, Ge L, Komarow L, Jiang J, Boutzoukas A, et al. Escherichia coli ST131 drives carbapenem use for E. coli bloodstream infections. Clin Infect Dis. 2026 Mar 6; doi:10.1093/cid/ciag160](https://ad-id.co/4ba03US)
  - Summary: This prospective cohort study of 282 US patients with Escherichia coli bloodstream infections found that ST131, particularly the C2/H30Rx subclade, accounted for the majority of ceftriaxone-resistant infections, were more common in older patients and those from long-term care facilities, and drove higher overall and empiric carbapenem use, although 30-day clinical outcomes were similar between ST131 and non-ST131 infections.

**Infection Prevention / Antibiotic Prophylaxis**

- [Blacksell SD, Le KK, Dhawan S, Wuthiekanun V, Limmathurotsukul D, Dunachie SJ, et al. Biosafety and biosecurity for Burkholderia pseudomallei and Burkholderia mallei: evidence, gaps, and sustainable practice in endemic, low-resource settings. Clin Microbiol Rev. 2026 Mar 11; doi:10.1128/cmr.00369-25](https://ad-id.co/4lxyJU0)
  - Summary: This review highlights the significant biosafety challenges posed by Burkholderia pseudomallei and Burkholderia mallei due to their high pathogenicity, antimicrobial resistance, and bioterrorism potential, emphasizing inconsistencies in laboratory risk classifications and protocols, the need for harmonized global standards, sustainable PPE and disinfection practices for low-resource settings, and targeted research on infectious dose, post-exposure prophylaxis, and transmission dynamics to improve laboratory safety and inform coherent international containment policies.
- [Khader K, Haroldsen C, Stevens V, Visnovsky L, Evans M, Simbartl L, et al. Estimating changes in facility MRSA infection rates due to changes in MRSA precaution policy. Clin Infect Dis. 2026 Mar 13; doi:10.1093/cid/ciag176](https://ad-id.co/3P25g8H)
  - Summary: This study of 121 VA acute care hospitals during the COVID-19 pandemic found that temporary discontinuation of methicillin-resistant Staphylococcus aureus (MRSA) prevention practices, including contact precautions and active surveillance, was not consistently associated with increased healthcare-associated infections after adjusting for baseline MRSA burden, underscoring the influence of facility-specific factors, unmeasured pandemic-related practices, and the need for flexible, context-sensitive infection prevention policies.
- [Igwilo-Alaneme R, Berbari E, McHugh J, Petri F, Ravi V, Matsuo T, et al. Cephalosporins versus non-cephalosporin antibiotics for perioperative prophylaxis in primary arthroplasty: a systematic review and meta-analysis. Clin Infect Dis. 2026 Mar 14; doi:10.1093/cid/ciag172](https://ad-id.co/4be4PAQ)
  - Summary: This systematic review and meta-analysis of 23 studies involving over 211,000 arthroplasties found that cephalosporins, particularly cefazolin and cefuroxime, are associated with significantly lower odds of prosthetic joint infection compared to non-cephalosporin prophylaxis, supporting guideline-recommended use in primary arthroplasty and highlighting the importance of adhering to cephalosporin-based perioperative antibiotic protocols.
- [Hagiwara J, Yoshioka K, Ito-Hagiwara K, Endo Y, Jafari D, Rolston DM, et al. Optimizing infection management after cardiac arrest: addressing diagnostic uncertainty and therapeutic dilemmas—a narrative review. J Intensive Care. 2026;14:20; doi:10.1186/s40560-026-00859-6](https://ad-id.co/3PCdww8)
  - Summary: This review highlights that infections are common and diagnostically challenging after cardiac arrest due to post-cardiac arrest syndrome, sedation, and targeted temperature management, with pneumonia predominating but bloodstream and intra-abdominal infections often under-recognized, emphasizing that optimal care requires protocolized prevention bundles, serial biomarker monitoring integrated with clinical assessment and imaging, early microbiological sampling, antimicrobial stewardship with timely de-escalation, and the development of PCAS-specific diagnostic tools and decision-support systems to improve patient outcomes.
- [Sanvitti M, Kanapeckas L, Bilotta F. Minimizing hospital acquired intensive care unit infections: a focus on prevention. World J Crit Care Med. 2026 Mar 9;15(1):113252; doi:10.5492/wjccm.v15.i1.113252](https://ad-id.co/4dl6aaa)
  - Summary: This narrative review emphasizes that preventing hospital-acquired infections in intensive care units requires coordinated, multifaceted strategies—including strict hand hygiene, proper use of personal protective equipment, vaccination, patient-level care bundles for device-related infections, and systemic measures such as adequate staffing and single-patient rooms—while highlighting that consistent implementation, continuous education, auditing, and adaptation of international guidelines to local contexts are essential to reduce morbidity and mortality.

**Gastrointestinal Tract Infections**

- [Park JY, Lee Y-C, Moayyedi P, Lansdorp-Vogelaar I, Camargo MC, Tepeš B, et al. Helicobacter pylori screen-and-treat programs for gastric cancer prevention — IARC working group report. N Engl J Med. 2026 Mar 11;394:1131–1137; doi:10.1056/NEJMsb2515372](https://ad-id.co/3NErAoj)
  - Summary: Gastric cancer remains a major global health burden, particularly in low- and medium-Human Development Index countries, and is largely caused by Helicobacter pylori infection; while clinical evidence supports H. pylori eradication as an effective preventive strategy, population-level screen-and-treat programs remain limited to a few regions, with most countries relying on costly endoscopic screening, highlighting the need for greater investment, scalable implementation guidance, and evaluation of feasibility, effectiveness, and potential harms to expand global gastric cancer prevention.
- [Eltorki M, Ajayi OO, Seok J, Xie J, Rizzuti FA, Berenger BM, et al. Shiga toxin–producing Escherichia coli outbreak in Canadian daycare centers. JAMA Netw Open. 2026 Mar 10;9(3):e261278; doi:10.1001/jamanetworkopen.2026.1278](https://ad-id.co/4lvqEz1)
  - Summary: This retrospective cohort study of a large pediatric Shiga toxin–producing Escherichia coli outbreak in Calgary, Canada, involving 285 children and 71 adults, found that significant health care resources were required, including 508 emergency department and 395 dedicated clinic visits, with 40 children hospitalized and 21 developing hemolytic uremic syndrome (HUS), and demonstrated that daily thrombotic microangiopathy screening accurately identified all future HUS cases, highlighting the value of coordinated public health responses and proactive laboratory monitoring in optimizing clinical outcomes.

**Sexually Transmitted Infections**

- [Elsener TA, Dillon JA, Shafer WM, Tang CM. Plasmids in Neisseria gonorrhoeae: drivers of DoxyPEP failure and an emerging threat for current therapy. Clin Microbiol Rev. 2026 Mar 10; doi:10.1128/cmr.00252-25](https://ad-id.co/40xYfif)
  - Summary: This review highlights that gonococcal disease poses a growing threat due to rising cases and antimicrobial resistance, with ceftriaxone as the primary treatment and doxycycline post-exposure prophylaxis (DoxyPEP) being increasingly used, while plasmids pbla and pConj in Neisseria gonorrhoeae confer resistance to β-lactams and tetracyclines, respectively, limiting DoxyPEP efficacy and facilitating the spread of resistance, emphasizing the need to understand plasmid biology, transfer dynamics, and evolutionary interactions to inform effective public health strategies for controlling and treating gonococcal infections.

**Respiratory Tract Infections**

- [Al-Zergani F, Meddis A, Clausen CL, Ravn P, Roldgaard MS, Kolte L, et al. Characterising the timing and causes of death in community-acquired pneumonia. Clin Microbiol Infect. 2026 Mar 13; doi:10.1016/j.cmi.2026.03.016](https://ad-id.co/4rvNWX6)
  - Summary: In a cohort of 2,918 adults hospitalized with community-acquired pneumonia, 19% died within 90 days, with pneumonia-related deaths—primarily from respiratory failure—occurring early in the first two weeks, while later deaths were mostly due to comorbidities or unknown causes, highlighting that CAP mortality is front-loaded and emphasizing the importance of cause-specific death assessment to guide treatment evaluation and improve outcomes in frail populations.
- [Fayos M, Burillo A, Galar A, Álvarez Uría A, Martín Loeches I, Vena A, et al. Questions and issues of non-ventilated hospital acquired pneumonia: an opinion document. Clin Microbiol Infect. 2026 Mar 14; doi:10.1016/j.cmi.2026.03.015](https://ad-id.co/4lvNpmF)
  - Summary: This narrative review critically examines non-ventilated hospital-acquired pneumonia (NV-HAP), highlighting the limited and heterogeneous evidence on its diagnosis, microbiological sampling, radiological evaluation, and empirical treatment, and provides expert insights, practical recommendations for clinical decision-making, and identifies key gaps and research priorities to guide future studies and improve patient management.
- [Jackson H, Hernandez Padilla AC, Vintcent LEM, Barac A, Cremer O, Daix T, et al. Perpetual observational study of the clinical and microbiological epidemiology of ventilator-associated pneumonia in Europe. Crit Care. 2026;30:112; doi:10.1186/s13054-025-05753-5](https://ad-id.co/4dk7TfV)
  - Summary: This prospective study of 3,446 patients across 25 European ICUs found that ventilator-associated pneumonia (VAP) occurred in 17.1% of at-risk patients, with significant variability between countries, predominantly caused by Staphylococcus aureus, Haemophilus influenzae, and Pseudomonas aeruginosa, and was associated with prolonged mechanical ventilation and higher ICU mortality (34.2% vs 29.3%), underscoring the ongoing need for optimized prevention and management strategies despite widespread implementation of measures such as head-of-bed elevation.

**Urinary Tract Infections**

- [Kinlin C, Gravel J, Barrowman N, Bijelik V, Cook R, Wills-Ibarra N, et al. Diagnosing urinary tract infection in young febrile children in the emergency department. JAMA Netw Open. 2026 Mar 13;9(3):e261741; doi:10.1001/jamanetworkopen.2026.1741](https://ad-id.co/413yDtN)
  - Summary: This prospective diagnostic study of 2,561 febrile children aged 2–24 months in two Canadian pediatric emergency departments found that UTICalc version 3.0 accurately predicted urinary tract infections, with AUROCs of 84.1% for the clinical model and 95.3% when combined with dipstick results, demonstrating high sensitivity and specificity across risk thresholds, and offering a practical tool to support evidence-based urine testing and treatment decisions, though it did not surpass experienced clinicians.

**Mycobacterial Infections**

- [Vargas DA, Fuertes-Bucheli JF, Sanchez-Hidalgo A, Palomares Velosa J, Lasso AM, Gupta AJ, et al. Diagnostic accuracy of molecular testing on saliva and oral swabs for pulmonary tuberculosis.](https://ad-id.co/4luMNNS)
  - Summary: In a nested case–control study of 648 participants in Colombia, molecular testing using Xpert MTB/RIF Ultra on saliva and oral swabs demonstrated high diagnostic accuracy for pulmonary tuberculosis, with saliva showing superior sensitivity (90.5%) compared with swabs (71.6%) while maintaining high specificity (saliva 95.8%, swab 99%), surpassing WHO targets for nonsputum TB diagnostics and indicating both methods are acceptable and feasible for patients.
- [Dougan TJ, Roth S, Xie L, D’Amaddio S, Walt DR. Antigen heterogeneity in the development and clinical validation of a multiplexed urine test for tuberculosis. Commun Med. 2026 Mar 10; doi:10.1038/s43856-026-01458-0](https://ad-id.co/4lAGlFs)
  - Summary: A multiplex Single Molecule Array (Simoa) assay measuring lipoarabinomannan (LAM) and antigen 85B (Ag85B) in urine was validated across 576 individuals from multiple countries, achieving 98% specificity and 45% overall sensitivity (58% in HIV-positive patients), demonstrating higher sensitivity than the AlereLAM test and offering a safe, noninvasive adjunctive tool for earlier tuberculosis diagnosis and treatment initiation.
- [Department for HIV, Tuberculosis, Hepatitis and Sexually Transmitted Infections. Near point-of-care nucleic acid amplification tests (NPOC-NAATs) as a new diagnostic class for diagnosis of TB using sputum and tongue swabs. World Health Organization. 2026 Mar 9.](https://ad-id.co/4lrYuF4)
  - Summary: WHO recommends near point-of-care nucleic acid amplification tests (NPOC-NAATs) for rapid TB diagnosis using sputum or, when unobtainable, tongue swabs in adults and adolescents, including people living with HIV; these battery-operated, swab-based molecular tests produce results within an hour, can be performed by minimally trained staff in peripheral or community settings, and should be followed by drug susceptibility testing since rifampicin resistance is not detected. The MTBC Nucleic Acid Test Card (Pluslife, China) meets WHO performance criteria for this class, providing ~30-minute results at low cost and portable operation, but new or brand-specific NPOC-NAATs require separate WHO evaluation and prequalification.
- [Calderwood CJ, Kunor T, Coleman M, Marambire E, Khan U, Herrera R, et al. Principles and priorities for integrated tuberculosis screening and care: a modified Delphi consensus exercise. PLOS Glob Public Health. 2026 Mar 2; doi:10.1371/journal.pgph.0005954](https://ad-id.co/4lrSRa0)
  - Summary: An international modified Delphi study with 324 panellists from 68 countries found strong consensus that tuberculosis (TB) services should be expanded and integrated for people with TB and their households, emphasizing routine screening for comorbid conditions, improved TB treatment outcomes, increased screening and preventive treatment among contacts, and integration with population-wide disease screening; these findings support further research, policy development, and evaluation of integrated TB service delivery.
- [Prodanuk M, King JW, Cunningham J, Kitai I, Piché-Renaud P-P, Ratnayake M, et al. Host blood biomarkers for the diagnosis of childhood tuberculosis disease: a systematic review and meta-analysis. Clin Microbiol Infect. 2026 Mar 6; doi:10.1016/j.cmi.2026.02.025](https://ad-id.co/3N7iAbg)
  - Summary: A systematic review of 55 studies on host blood biomarkers for childhood tuberculosis (TB) found multiple promising biomarkers, including cytokines, metalloproteinases, and miRNA, that met the WHO target product profile for TB diagnosis, but most were single-centre studies and lacked validation; meta-analysis of interferon-γ-inducible protein 10 (IP-10) showed insufficient accuracy, highlighting the need for high-quality, multicentre studies and development of point-of-care tests suitable for low-resource settings to improve pediatric TB diagnosis.
- [Yoshida S, Matsumoto Y, Kajihara A, Funato M, Tsuyuguchi K, Mitarai S, et al. Fomite transmission of Mycobacterium abscessus between severely disabled patients. Clin Microbiol Infect. 2026 Mar 9; doi:10.1016/j.cmi.2026.02.029](https://ad-id.co/4luR7Nj)
  - Summary: A 34-month investigation of a nosocomial outbreak of Mycobacterium abscessus subspecies massiliense (MAM) in an Osaka hospital identified seven patients and demonstrated through genomic sequencing that clinical and environmental isolates were nearly identical, with subclone analysis indicating that fomites, such as a wagon used in patient rooms, likely facilitated indirect transmission, highlighting the pathogen’s ability to persist in dry hospital environments and the importance of environmental infection control measures.
- [McKenna L, Frick M, Alarcón-Guizado VA, Davies G, Furin JJ, Guglielmetti L, et al. When are uncontrolled trials in TB scientifically and ethically justified: borrowing wisdom from early AIDS clinical trials. Clin Infect Dis. 2026 Mar 13; doi:10.1093/cid/ciag175](https://ad-id.co/4lsQnbD)
  - Summary: An analysis of tuberculosis treatment trial designs concluded that existing frameworks do not support the use of uncontrolled phase 3 trials for novel regimens, indicating that all phase 3 TB trials should include appropriate control groups to ensure valid and reliable assessment of treatment efficacy.
- [Moe CA, Luswata RK, Barrameda AJ, Le H, Muzazu S, Crowder R, et al. Diagnostic yield of tongue swab- compared to sputum-based molecular testing for tuberculosis in four high-burden countries. Clin Infect Dis. 2026 Mar 10; doi:10.1093/cid/ciag077](https://ad-id.co/4rt3lax)
  - Summary: In a multicountry study, tongue swab-based molecular testing for tuberculosis using the MiniDock MTB platform achieved a diagnostic yield non-inferior to sputum-based molecular testing, supporting its use as a feasible, cost-efficient alternative in settings where sputum collection is difficult or smear microscopy is the main diagnostic method.
  - **Editorial Commentary:**[Barer MR. Are we ready for the gift tongues can give to TB? Clin Infect Dis. 2026 Mar 10; doi:10.1093/cid/ciag078](https://ad-id.co/415UlNO)
- [Kosenko M, Davtian L, Iakovleva E, Ashurov M, Podgalo D, Oganezova JG, et al. Shorter antitubercular regimens versus 9 months of isoniazid for latent tuberculosis in children: a systematic review and meta-analysis. Clin Infect Dis. 2026 Mar 10; doi:10.1093/cid/ciag073](https://ad-id.co/4ryarL0)
  - Summary: A systematic review and meta-analysis found that shorter rifamycin-containing regimens for treating latent tuberculosis infection in children likely improve treatment completion, maintain similar safety, and show no meaningful difference in preventing TB disease compared with the standard 9-month isoniazid regimen, supporting current guideline recommendations favoring shorter courses.
- [Yang E, Van Brantegem P, Peloquin CA, Brooks MB, Coit JM, Vargas Vásquez D, et al. Pharmacokinetic and pharmacogenomic predictors of hepatotoxicity in the HIRIF trial for drug-susceptible tuberculosis. Clin Infect Dis. 2026 Mar 6; doi:10.1093/cid/ciag141](https://ad-id.co/4lvmOWB)
  - Summary: In an analysis of the HIRIF trial, higher pyrazinamide exposure was independently associated with hepatotoxicity during standard tuberculosis therapy, while rifampin exposure was not; isoniazid exposure and slow NAT2 acetylator status were linked to hepatotoxicity in univariable analysis, highlighting pyrazinamide as the primary driver of liver enzyme elevations.
- [Yanagisawa S, Todoroki Y, Takechi H, Wasamoto S. Cobblestone airway from Mycobacterium avium infection. Am J Respir Crit Care Med. 2026 Feb;212(2):354–355; doi:10.1164/rccm.202412-2504IM](https://ad-id.co/4dlX7Wr)
  - Summary: A 46-year-old healthy nonsmoking woman presented with gradually worsening chest radiograph abnormalities; chest CT showed disseminated centrilobular nodules and cavitary lesions in the right upper lobe. Despite a negative ELISPOT, a positive anti-glycopeptidolipid-core IgA antibody suggested pulmonary Mycobacterium avium complex (MAC) infection. Bronchoscopy revealed cobblestone-like airway changes, and transbronchial biopsy showed granulomas, while bronchoalveolar lavage grew M. avium. She was diagnosed with MAC lung disease and treated with azithromycin and ethambutol, resulting in significant radiographic improvement.

**Fungal Infections and Antifungal Agents**

- [Lass-Flörl C, Lass M, Kern JM, Huber S. Antifungal susceptibility testing across fungi: why MICs vary, methods diverge, and what MIC can miss. Clin Microbiol Infect. 2026 Mar 11; doi:10.1016/j.cmi.2026.03.004](https://ad-id.co/4lvzInK)
  - Summary: Antifungal susceptibility testing (AFST) is essential for guiding antifungal therapy, but minimum inhibitory concentration (MIC) results can vary due to technical differences, fungal growth phenotypes, and methodological variability. Factors such as inoculum size, incubation conditions, medium, endpoint definitions, and non-binary growth (e.g., azole trailing) can influence MICs, while limited clinical breakpoints and inter-method non-interchangeability add to interpretation challenges. Careful standardization, reporting of methods, cautious interpretation of near-cutoff results, and confirmation with reference methods or targeted resistance testing are recommended to ensure reliable clinical decision-making.
- [Barac A, Cornely OA, Paño-Pardo JR, Gupta N, Jokelainen P, Mora-Rillo M, et al. Emerging mycoses: a neglected area of concern for global health. Clin Microbiol Infect. 2026 Mar 13; doi:10.1016/j.cmi.2026.03.013](https://ad-id.co/3PBPCAS)
  - Summary: Fungal infections are an increasingly significant global health threat, driven by environmental changes, climate change, deforestation, human mobility, immunosuppression, and limited healthcare capacity. Emerging pathogens such as Blastomyces helicus, Candidozyma auris, Emergomyces spp., and Sporothrix brasiliensis demonstrate shifting epidemiology, yet fungal diseases remain neglected in health policy, diagnostics, and pandemic preparedness. Strengthening surveillance, equitable access to diagnostics and antifungals, and health system readiness across human, animal, and environmental interfaces is critical to mitigate the growing public health impact of mycoses.
- [Stemler J, Sprute R, Koehler P, Cornely OA. How to safely discontinue antifungal treatment in invasive pulmonary aspergillosis? – Clinical considerations in haematology. Clin Microbiol Infect. 2026 Mar 6; doi:10.1016/j.cmi.2026.03.001](https://ad-id.co/4rBFbKV)
  - Summary: In invasive pulmonary aspergillosis (IPA) among patients with hematological malignancies, antifungal treatment duration is often empirically determined due to a lack of standardized clinical trial data. Safe discontinuation should be guided by a combination of host immune recovery, clinical stability, mycological markers (e.g., serum galactomannan), and imaging findings, with structured post-cessation surveillance to detect recurrence early. Decisions must balance the risk of relapse, especially in immunocompromised patients, against cumulative drug toxicity, interactions, and quality-of-life considerations, highlighting the need for standardized, patient-specific cessation protocols and prospective trials to optimize therapy duration.
- [Ghai RR, Benedict K, Chiller TM, Chow NA, Hennessee IP, Jones S, et al. Contribution of fungal diseases to the U.S. chronic disease burden. Clin Microbiol Rev. 2026 Mar 5; doi:10.1128/cmr.00085-25](https://ad-id.co/4lOwA6J)
  - Summary: Fungal infections, particularly severe and antifungal-resistant cases, play a significant but underexplored role in the development and exacerbation of chronic diseases in the United States. While chronic conditions are known to increase susceptibility to invasive fungal infections, fungal pathogens themselves can directly contribute to chronic disease through pathophysiologic effects and treatment-related toxicities. This review underscores the need for further research, enhanced surveillance, greater public and clinical awareness, and improved access to diagnostics and antifungal therapies to mitigate the impact of fungal infections on chronic disease burden.
- [Angelini J, Ferin S, Martini L, Flammini S, Tascini C, Giuliano S. Current opinion on the potential role of liposomal amphotericin B in underexplored clinical scenarios. Clin Microbiol Rev. 2026 Mar 13; doi:10.1128/cmr.00228-25](https://ad-id.co/4rzspwH)
  - Summary: The rising incidence of invasive fungal infections caused by resistant Candida species and molds poses significant challenges, especially in immunocompromised patients, due to resistance, drug interactions, and toxicity associated with standard antifungals. Liposomal amphotericin B (L-AmB), with broad-spectrum fungicidal activity, reduced nephrotoxicity, and minimal CYP450 interactions, may be a valuable alternative in high-risk scenarios, including resistant or biofilm-associated infections, hepatic vulnerability, and prophylaxis in hematologic patients. Although clinical evidence remains limited, selective use of L-AmB in cases where conventional therapies are inadequate or contraindicated is supported by expert insight and warrants further research.
- [Stephens AV, Thauland TJ, Whitehill GD, Garcia-Lloret MI, Butte MJ. Immunomodulation in the treatment of disseminated coccidioidomycosis. Clin Infect Dis. 2026 Mar 4; doi:10.1093/cid/ciag036](https://ad-id.co/3NjodmM)
  - Summary: In a case series of 18 patients with severe or persistent disseminated coccidioidomycosis at UCLA, adjunctive immunomodulatory therapy—recombinant interferon gamma for severe disease and dupilumab for Type-2 immune dysregulation—was associated with improved clinical outcomes and an 88% survival rate, suggesting that targeted immunomodulation may enhance treatment effectiveness in this population and supporting the need for clinical trials to define optimal patient selection and therapy strategies.
  - **Editorial Commentary:**[Donovan FM. Immunomodulation in disseminated coccidioidomycosis: a small step or a giant leap? Clin Infect Dis. 2026 Mar 4; doi:10.1093/cid/ciag037](https://ad-id.co/4lqsr8w)
- [Deivarajan HR, Nachammai D, Nandhakumar D, Jaisankar D, Ganapathi B, Jaju SS, et al. Diagnostic accuracy of a CRISPR-based assay in smear- and culture-negative fungal keratitis. JAMA Ophthalmol. 2026 Mar 5; doi:10.1001/jamaophthalmol.2026.0113](https://ad-id.co/4bl525j)
  - Summary: In patients with smear- and culture-negative fungal keratitis, the CRISPR-based RID-MyC assay demonstrated good sensitivity (82.1%) and fair specificity (76.9%) compared with in vivo confocal microscopy, showing 80.5% concordance; its rapid, accessible format suggests it could serve as a practical adjunct diagnostic tool, particularly in resource-limited settings or when imaging is unavailable.
- [Maertens JA, Vanbiervliet Y, Mercier T, Aerts R, Lagrou K, Slavin MA. 25 years of improvement in mortality in invasive aspergillosis in haematology patients: will it be sustained or is it under threat? J Antimicrob Chemother. 2026 Mar 6; doi:10.1093/jac/dkag077](https://ad-id.co/3PCO61n)
  - Summary: Mortality from invasive aspergillosis has steadily declined over recent decades, largely due to routine use of mold-active antifungals, particularly azoles, alongside advances in diagnostics, targeted therapy, and management of underlying diseases, though the precise contribution of improved diagnostics and supportive care is difficult to quantify; emerging azole resistance, mixed-species infections, and drug interactions now highlight the need for novel antifungal strategies.

**Virulence**

- [Brizuela J, Murray GGR, Boueroy P, Balmer AJ, Wongsurawat T, Jenjaroenpun P, et al. Emergence of novel zoonotic and multi-drug resistant Streptococcus suis lineages. Clin Microbiol Infect. 2026 Mar 13; doi:10.1016/j.cmi.2026.03.012](https://ad-id.co/4lussIP)
  - Summary: Whole-genome sequencing of Thai Streptococcus suis strains revealed that two endemic lineages, CC104 and CC233, recently emerged through capsule switching from CC1 and acquired multiple antimicrobial resistance genes—including resistance to penicillin and ceftriaxone—highlighting rapid evolution of multi-drug-resistant zoonotic strains and underscoring the urgent need for enhanced surveillance, infection control, and treatment strategies.

**Improving Clinical Research**

- [Julious SA, Totton N. Considerations for improving non-inferiority trials. Lancet. 2026 Mar 14;407(10533):1038-1039. doi:10.1016/S0140-6736(26)00306-5](https://ad-id.co/4rvnVHi)
  - Summary: Non-inferiority trials are increasingly used to evaluate new treatments with potentially different benefits, but careful attention to design and analysis is essential to address methodological challenges and ensure high-quality, reliable research and publication outcomes.
- [Yelland LN, Lange KM, Braat S, Robledo KP, Cuthbert AR, Sullivan TR. When randomisation goes horribly wrong: examples of major failures of randomisation and strategies to avoid them. Trials. 2026;27:84. doi:10.1186/s13063-025-09390-9](https://ad-id.co/4bcubis)
  - Summary: Randomisation is a cornerstone of clinical trials, but major errors—such as incorrect schedules, cluster mismanagement, or programming mistakes—can compromise trial validity and lead to retractions; careful planning, documentation, staff training, and thorough testing are essential to prevent such failures and ensure transparency when they occur.
- [Chesnaye NC, Ortiz A, van Diepen M, Dekker F, Zoccali C, Tripepi G, et al. How to interpret the number needed to treat for clinicians. Nephrol Dial Transplant. 2026 Mar;41(3):437–44. doi:10.1093/ndt/gfaf168](https://ad-id.co/4dn2BQQ)
  - Summary: The number needed to treat (NNT) is a practical measure for evaluating intervention effectiveness, with related concepts like number needed to harm (NNH) and likelihood to be helped or harmed; accurate calculation and interpretation require attention to baseline risk, time, confidence intervals, and common pitfalls to support informed clinical decision-making.
- [Ban J-W, Madsen T, Robinson KA, Lund H. Determining the conclusiveness of systematic review evidence: a scoping review of methodological approaches. Ann Intern Med. 2026 Mar 10; doi:10.7326/ANNALS-25-0279](https://ad-id.co/3P5XD0Z)
  - Summary: This review mapped 62 methods for assessing the conclusiveness of systematic reviews, including 29 mathematical methods for meta-analyses, 15 for cumulative meta-analyses, and 18 nonmathematical approaches such as GRADE and RAND, highlighting diverse strategies to determine whether systematic review findings can reliably guide research, funding, and clinical decisions.
- [Arlett P, Umuhire D, Verpillat P, Foggi P, Liminga UW, Sepodes B, et al. Clinical Evidence 2030. Clin Pharmacol Ther. 2025 Feb 14; doi:10.1002/cpt.3596](https://ad-id.co/40xJJah)
  - Summary: The generation of high-quality clinical evidence is essential for informed decisions on medicine development, authorization, reimbursement, and use; in Europe, evolving factors—including lessons from COVID-19, new legislation like the European Health Data Space, regulatory reforms, advanced analytics, and greater patient involvement—create opportunities to strengthen and accelerate sustainable, collaborative, and innovative approaches to evidence generation, guided by six key principles.

**General Interest**

- [Hornuss D, Mathé P, Giesen R, Escolà-Vergé L, Isler B, Bartoletti M, et al. Possible quality indicators for clinical infectious diseases consultations – results from a hybrid Delphi-nominal group approach and scenario study. Clin Microbiol Infect. 2026 Mar 12; doi:10.1016/j.cmi.2026.03.006](https://ad-id.co/417eCCA)
  - Summary: Infectious diseases consultation (IDC) services improve infection management, but standardized guidance on performance and reporting is lacking; this consensus study identified 25 quality indicators across four domains—history and risk factors, bedside assessment, recommendations, and reporting—highlighting thorough conduct, documentation, and potential for automation or AI support, with consultation times ranging from 35 to 55 minutes, providing a framework to standardize IDC evaluation, enhance effectiveness, and support international benchmarking, though further validation in diverse clinical settings is needed.
- [DePledge L, Shi LZ. Soft Tick Relapsing Fever. N Engl J Med. 2026 Mar 11;394:e18. doi:10.1056/NEJMicm2514982](https://ad-id.co/4dtoSMO)
  - Summary: A 74-year-old man developed recurrent fevers, myalgia, and vomiting after attending a historic cabin in Yosemite National Park, and thin blood smears revealed spirochetes; PCR confirmed Borrelia species, leading to a diagnosis of soft tick relapsing fever, likely transmitted by Ornithodoros hermsi ticks, and treatment with doxycycline with monitoring for a Jarisch–Herxheimer reaction resolved his symptoms within one month.
- [Tran A, Lee C. Massive Intravascular Hemolysis from Clostridium perfringens Bacteremia. N Engl J Med. 2026 Mar 11;394:e19. doi:10.1056/NEJMicm2514135](https://ad-id.co/4rRcNF7)
  - Summary: A 73-year-old woman with type 2 diabetes presented in shock with malaise, dyspnea, and confusion, and her blood samples were grossly hemolyzed; she suffered a pulseless electrical activity cardiac arrest and died, and postmortem examination revealed massive intravascular hemolysis with intracellular and extracellular bacilli, with blood cultures growing Clostridium perfringens and Klebsiella pneumoniae, leading to a diagnosis of toxin-mediated C. perfringens bacteremia, a rare but highly fatal condition.

**Target Trial Emulation**

- [Bartoletti M, Rosselli Del Turco E, Bussini L, Paul M, Fares Sabik E, Castagna A, et al. Monotherapy vs combination therapy for Enterococcus faecalis bacteremia: a target trial emulation. Clin Microbiol Infect. 2026 Mar 16; doi:10.1016/j.cmi.2026.03.018](https://ad-id.co/4154UAL)
  - Summary: In a multicenter international cohort of 373 adults with Enterococcus faecalis bloodstream infection without endocarditis, combination antibiotic therapy (e.g., ampicillin plus ceftriaxone or gentamicin) did not improve 90-day clinical outcomes compared to monotherapy, with similar rates of composite failure (death, relapse, or endocarditis), suggesting that simpler monotherapy may be sufficient and support antimicrobial stewardship, while sepsis or septic shock at presentation was the main factor associated with failure.

**Trial Protocols / Trial Ideas**

- [Mawson P, Morton M, Walmsley Z, Wafer R, Hancock HC, Mossop H, et al. SHORTER trial: protocol for a pragmatic, multicentre, randomised controlled trial of short-duration antibiotic therapy for critically ill patients with sepsis. BMJ Open. 2026; doi:10.1136/bmjopen-2026-117142](https://ad-id.co/4dkTRL7)
  - Summary: The SHORTER trial is a large multicentre UK randomised controlled study enrolling 2244 critically ill adults with suspected or confirmed sepsis to evaluate whether a fixed 5-day antibiotic course is non-inferior to standard-duration therapy for 28-day mortality while reducing antibiotic use, with additional assessment of longer-term outcomes, readmissions, infection rates and cost-effectiveness to address uncertainty around optimal antibiotic duration in sepsis.
- [Banerjee R, Komarow L, Li Y, Wu Q, Sanchez-Gonzalez L, Mau D, et al. Study protocol for a multicenter, multinational prospective randomized controlled trial comparing outcomes in subjects with Gram-negative bacteremia who have blood culture evaluation using Fast Antibiotic Susceptibility Testing vs. standard of care testing: the FAST trial. Trials. 2025 Nov 12;26:500. doi:10.1186/s13063-025-09228-4](https://ad-id.co/3NzBF6b)
  - Summary: This multicentre multinational randomised controlled trial of 900 hospitalized patients with Gram-negative bloodstream infections compares rapid phenotypic antibiotic susceptibility testing using VITEK REVEAL™ plus standard care versus standard methods alone to determine whether faster results improve clinical outcomes, including a composite desirability ranking, mortality, length of stay, and timeliness of antibiotic optimisation in high-resistance settings.

**Guidelines**

- [Prescott HC, Antonelli M, Alhazzani W, Møller MH, Alshamsi F, Azevedo LCP, et al. Surviving Sepsis Campaign: International Guidelines for Management of Sepsis and Septic Shock 2026. Crit Care Med. 2026 Mar 23; doi:10.1097/CCM.0000000000007075](https://ad-id.co/4bXT8x0)
  - Summary: The 2023 Surviving Sepsis Campaign guidelines provide evidence-based recommendations for the identification, management, and post-hospital care of adult patients with sepsis, emphasizing early intervention, antimicrobial stewardship, and patient-centered outcomes; developed by a 69-member international committee with diverse clinical and geographic representation including LMICs, the guidelines used rigorous PICO-based evidence synthesis, consensus voting, and patient/family input to produce actionable guidance across six clinical domains, supporting clinicians and health systems in improving sepsis outcomes globally.

**Antibiotic Therapy Reviews**

- [Gatti M, Pea F. Pharmacological approaches to overcome antimicrobial resistance in gram-negative bacterial infections: current practice and future directions. Expert Opin Pharmacother. 2026 Mar 27; doi:10.1080/14656566.2026.2651279](https://ad-id.co/4dTaL3I)
  - Summary: This review highlights the critical need to prevent resistance development among Gram-negative pathogens and emphasizes that optimizing the pharmacokinetic/pharmacodynamic (PK/PD) use of novel beta-lactams and beta-lactam/beta-lactamase inhibitor combinations—through aggressive PK/PD targets, prolonged or continuous infusion, and therapeutic drug monitoring—can help preserve antimicrobial efficacy, supported by both preclinical and clinical evidence.
- [Lyu S, Luo J, Liu P, Qin X, He W, Jing G, et al. Inhaled antibiotics to treat ventilator-associated pneumonia: a systematic review and meta-analysis. Crit Care Med. 2026 Mar 16; doi:10.1097/CCM.0000000000007072](https://ad-id.co/41850rp)
  - Summary: This systematic review and meta-analysis of 32 RCTs and 41 non-RCTs demonstrates that adjunctive inhaled antibiotics in ventilator-associated pneumonia significantly improve clinical cure, microbiological eradication, and reduce all-cause mortality—particularly in VAP-only populations—while also decreasing emergence of new drug resistance, and exploratory analyses suggest potential benefits over intravenous therapy, including shorter ventilator duration and lower nephrotoxicity, supporting the need for further high-quality trials.

**PK/PD and Drug Dosing**

- [Timsit JF, Joannes-Boyau O, Bracht H. The time has come for optimizing beta-lactam therapy in critically ill patients with renal dysfunction. Intensive Care Med. 2026 Mar 23; doi:10.1007/s00134-026-08376-8](https://ad-id.co/4bUvBgv)
  - Summary: Achieving effective antibiotic therapy in critically ill patients requires rapidly attaining optimal β-lactam pharmacokinetic/pharmacodynamic (PK/PD) targets, as subtherapeutic concentrations in the first 24–48 hours increase the risk of treatment failure, resistance, and mortality; strategies such as continuous or prolonged infusion, model-informed precision dosing, nomograms for initial dosing, and therapeutic drug monitoring (TDM) can improve target attainment, particularly in patients with altered pharmacokinetics or on renal replacement therapy, though clinical trials show mixed results on survival benefits and TDM remains primarily a supportive tool for dose adjustment rather than a proven means to improve outcomes.
- [van den Berg S, Pieren M, Sassen SDT, de Jong WAM, Boonman CSC, Dale GE, et al. The pharmacodynamics of polymyxin B in Acinetobacter baumannii in murine thigh and lung infection models. J Antimicrob Chemother. 2026 Apr;81(4):dkag097. doi:10.1093/jac/dkag097](https://ad-id.co/3PxKX3f)
  - Summary: This preclinical study using murine models shows that while polymyxin B achieves effective bacterial killing in thigh infections caused by Acinetobacter baumannii, its efficacy is limited in lung infections, and standard human dosing regimens are unlikely to reach pharmacodynamic targets without exceeding toxicity thresholds, indicating that polymyxin B monotherapy may be insufficient for treating A. baumannii infections.

**Antibiotics - In vitro susceptibility**

- [Kunz Coyne AJ, Gray R, Gonnabathula P, May ES, Tamma PD, Do A, et al. Comparative in vitro activity of aztreonam–avibactam and aztreonam plus ceftazidime–avibactam against Stenotrophomonas maltophilia complex. Antimicrob Agents Chemother. 2026 Mar 24; doi:10.1128/aac.01456-25](https://ad-id.co/4dkuI3i)
  - Summary: This in vitro study demonstrates that aztreonam–avibactam (ATM–AVI) and aztreonam plus ceftazidime–avibactam (ATM–CZA) have comparable bactericidal activity against Stenotrophomonas maltophilia complex isolates, though isolate-specific differences exist, with ATM–AVI showing sustained killing in blaL2-dominant strains and ATM–CZA more effective against blaL1 or smeABC-dominant strains, highlighting the need for further investigation into genotype–phenotype relationships to guide optimal therapy.
- [Gashaw Y, Sisay A, Getachew E, Asmare Z, Geteneh A, Tamrat E, et al. Colistin-resistance among Acinetobacter baumannii and Pseudomonas aeruginosa from clinical specimens in Africa: a systematic review and meta-analysis. JAC Antimicrob Resist. 2026 Apr;8(2):dlag039. doi:10.1093/jacamr/dlag039](https://ad-id.co/4s5bl1W)
  - Summary: This systematic review and meta-analysis of African clinical isolates shows a rising prevalence of colistin resistance in Acinetobacter baumannii (13.75%) and Pseudomonas aeruginosa (14.42%) with substantial geographic variation and increasing trends over time, highlighting the urgent need for strengthened antimicrobial stewardship, infection control, and molecular surveillance to curb the spread of resistance.
- [Pham TH, Molina KC, Huang V. Predicting oral cephalosporin susceptibility in Escherichia coli blood isolates using parenteral cephalosporin susceptibility testing. J Antimicrob Chemother. 2026 Apr;81(4):dkag093. doi:10.1093/jac/dkag093](https://ad-id.co/4s3wlWA)
  - Summary: This study evaluated whether parenteral cephalosporin susceptibility testing can predict oral cephalosporin activity in Escherichia coli bloodstream isolates and found that ceftriaxone and cefotaxime reliably predict oral third-generation cephalosporin susceptibility, while cefazolin MIC ≤2 mg/L may serve as a surrogate for oral second-generation agents despite high rates of minor and major errors, indicating the need for further clinical validation.

**Beta-Lactamases and Other Resistance Mechanisms**

- [Nguyen HA, Peleg AY, Wisniewski JA, Wang X, Wang Z, Blakeway LV, et al. AMR-GNN: a multi-representation graph neural network framework to enable genomic antimicrobial resistance prediction. Nat Commun. 2026 Mar 6; doi:10.1038/s41467-026-69934-8](https://ad-id.co/4dkqXLk)
  - Summary: AMR-GNN is a graph neural network-based framework that integrates multiple genomic representations to predict antimicrobial resistance phenotypes from whole-genome sequencing data, demonstrated in Pseudomonas aeruginosa, and validated across a large dataset of Gram-negative and Gram-positive pathogens, providing improved performance, mitigation of clonal bias, and identification of informative biomarkers to enhance explainability and applicability in diverse clinically relevant pathogen-drug combinations.
- [Beh JQ, Howden BP, Webb JR, Connor CH. Global dissemination of optrA-mediated linezolid resistance in enterococci. J Antimicrob Chemother. 2026 Apr;81(4):dkag089. doi:10.1093/jac/dkag089](https://ad-id.co/4djjH2f)
  - Summary: This genomic analysis of 565 enterococcal genomes reveals that the linezolid-resistance gene optrA is more frequently plasmid-borne in E. faecium than E. faecalis, with diverse genetic contexts including transposons (Tn6674, Tn6261) and multidrug-resistant fexA-optrA-erm(A)/(B) units, often mobilized by IS1216E elements, highlighting the critical role of mobile genetic elements in the global spread of optrA and the associated public health risk of plasmid-mediated drug-resistant enterococci.
- [Wang J, Zhang X, Zhang Y, Xie Q, Guo Y, Deng Q, et al. Emergence of carbapenem-resistant Escherichia coli coharboring blaCTX-M-199 and penicillin-binding protein 3 insertion conferring increased aztreonam/avibactam resistance in South China. J Infect Dis. 2026 Mar 15;233(Suppl_1):S81–S91. doi:10.1093/infdis/jiag025](https://ad-id.co/3O06elo)
  - Summary: This study identifies the emerging blaCTX-M-199 gene in carbapenem-resistant Escherichia coli isolates from South China, predominantly within high-risk clones ST410 and ST167, and demonstrates that blaCTX-M-199, often combined with YRIK/YRIN penicillin-binding protein 3 insertions and coharboring blaNDM-5, substantially reduces susceptibility to aztreonam/avibactam, highlighting the urgent need for surveillance and clinical detection of these multidrug-resistant strains.
- [González-Díaz A, Pinto M, Cadenas-Jiménez I, Duarte S, Ardanuy C, Ribeiro MM, et al. First identification and molecular characterization of CTX-M-15 extended-spectrum β-lactamase and OXA-9 β-lactamase in Haemophilus influenzae in the Iberian Peninsula. Microb Genet. 2026 Jan 30; doi:10.1128/aac.01649-25](https://ad-id.co/4djjHiL)
  - Summary: This study reports, for the first time, CTX-M-15 and OXA-9 β-lactamases in Haemophilus influenzae strains from Portugal and Spain, carried on mobile integrative elements (ICEHpaHUB5-like and ICEHinHUB1), revealing multidrug resistance, genomic plasticity, and an expanded resistome, underscoring the need for ongoing genomic surveillance and caution in treatment strategies.

**Antibiotic Stewardship**

- [Walker MK, Yek C, Sarzynski S, Warner S, Harris AD, Baghdadi JD, et al. Survival trends in patients with difficult-to-treat, antibiotic-resistant, Gram-negative infections in the era of next-generation antibiotics in the USA: a retrospective cohort study. Lancet Infect Dis. 2026 Mar 25; doi:10.1016/S1473-3099(26)00020-4](https://ad-id.co/4c0qews)
  - Summary: This retrospective US study of 5,065 adult inpatient encounters with difficult-to-treat resistant (DTR) Gram-negative infections from 2016 to 2023 found that, despite increased availability and use of newer DTR-active antibiotics, most patients continued to receive in-vitro discordant initial therapy and overall adjusted mortality remained largely unchanged for Enterobacterales, Pseudomonas aeruginosa, and Acinetobacter baumannii infections, except for a modest decrease in P. aeruginosa bloodstream infections, highlighting that timely identification of pathogens and resistance profiles remains critical to improving outcomes.
  - **Editorial Commentary:**
    [Kabbani S, McDonald LC. Treatment of multidrug-resistant Gram-negative infections: a stewardship imperative. Lancet Infect Dis. 2026 Mar 25; doi:10.1016/S1473-3099(26)00113-1](https://ad-id.co/4djjHPN)
- [Thomas A, Vogrin S, Batrouney A, Devchand M, Khumra S, Narayanasamy S, et al. Sustaining stewardship: longitudinal evaluation of an integrated antimicrobial programme in the ICU. J Antimicrob Chemother. 2026 Apr;81(4):dkag086. doi:10.1093/jac/dkag086](https://ad-id.co/4djjNqD)
  - Summary: A 7-year prospective study of an ICU electronic medical record–driven antimicrobial stewardship (AMS) ward round at Austin Health showed high acceptance of AMS recommendations, particularly for antibiotic escalation, and sustained reductions in broad-spectrum antimicrobial use (piperacillin/tazobactam, meropenem, ciprofloxacin, vancomycin), with increased amoxicillin/clavulanate suggesting compensatory prescribing, demonstrating the long-term effectiveness and sustainability of the ICU-AMS programme.
- [Rafiq S, Shi C, Ghosal S, Dark P, Felton T, Kontopantelis E, et al. Clinical effectiveness of procalcitonin- or C-reactive protein-guided antibiotic discontinuation protocols for adult patients who are critically ill with sepsis: a rapid systematic review and meta-analysis. Anaesthesia. 2026 Jan 8; doi:10.1111/anae.70109](https://ad-id.co/4dOgub2)
  - Summary: Moderate-certainty evidence from 19 trials including 6382 critically ill patients with sepsis indicates that procalcitonin-guided protocols probably reduce antibiotic duration by about 2 days without increasing mortality, whereas evidence for C-reactive protein-guided protocols remains limited, highlighting the potential role of procalcitonin in guiding safe and effective antimicrobial stewardship in critical care.
- [Myatra SN, Boyer KM, Hidalgo JL, Maves RC, Acharya SP, Jacob ST, et al. Gaps and strategies for management of sepsis in low-resource settings: expert consensus statements using a Delphi method. Crit Care Med. 2026 Mar 20; doi:10.1097/CCM.0000000000007102](https://ad-id.co/4bSqP31)
  - Summary: An international expert panel used a literature review and Delphi process to develop 58 consensus clinical practice statements addressing prevention, early recognition, timely management, and post-sepsis care in low-resource settings, highlighting gaps in evidence while providing actionable guidance to complement existing international sepsis guidelines.
- [Kortz TB, Hidalgo JL, Akech SO, Myatra SN, Maves RC, Perez-Fernandez J, et al. Ten steps to improve sepsis care in low-resource settings. Crit Care Med. 2026 Mar 20; doi:10.1097/CCM.0000000000007090](https://ad-id.co/4dkTW1n)
  - Summary: A consensus-based process involving literature review, Delphi surveys, stakeholder input, and expert conferences produced ten actionable, nonsequential steps to improve sepsis care in low-resource settings, emphasizing governance, education, prevention, early recognition, timely interventions, post-sepsis care, data systems, quality improvement, culture, and holistic well-being to guide health leaders, clinicians, and policymakers in strengthening the sepsis chain of survival and reducing global inequities in outcomes.

**Infection Prevention / Antibiotic Prophylaxis**

- [Rinaldi M, Miani B, Gibertoni D, Di Chiara M, Andrade Lopes C, Cona A, et al. Targeted peri-operative prophylaxis in patients colonized with carbapenem resistant Enterobacterales undergoing liver transplantation: a multinational cohort study. Clin Infect Dis. 2026 Mar 25; doi:10.1093/cid/ciag185](https://ad-id.co/4bXqpsj)
  - Summary: In a multinational cohort of 408 liver transplant recipients colonized with CRE, targeted perioperative prophylaxis using novel β-lactam/β-lactamase inhibitors or cefiderocol (T-PAPnew) significantly reduced CRE infections within the first 15 days post-transplant, whereas standard prophylaxis or older regimens (T-PAPold) showed no protective effect, though the benefit of T-PAPnew diminished by 30 days.
- [Martelli M, Rosa A, Miranda M, Simone R, Scarpati Cioffari di Castiglione M, De Falco F, et al. Oral hygiene management in critically ill patients: prevention of ventilator-associated pneumonia. Front Dent Med. 2026 Feb 19;7:1748329. doi:10.3389/fdmed.2026.1748329](https://ad-id.co/4dpRYNr)
  - Summary: Maintaining oral hygiene in critically ill, mechanically ventilated patients is essential for preventing ventilator-associated pneumonia (VAP) and other infections; evidence from systematic reviews indicates that combining mechanical plaque removal with chlorhexidine significantly reduces VAP incidence, and implementing standardized, multidisciplinary oral care protocols—including dental specialist involvement—optimizes patient outcomes and overall well-being, though further research is needed to refine techniques and standardize practices.

**Bloodstream Infections**

- [Cona A, Curatolo C, Giordani P, D'Andrea F, Bellavia D, Campanella M, et al. Association between unrecognized donor bloodstream infection and outcomes in solid organ transplant recipients: a prospective observational cohort study. Clin Microbiol Infect. 2026 Mar 26; doi:10.1016/j.cmi.2026.03.026](https://ad-id.co/4sovjoB)
  - Summary: In a prospective study of 968 solid organ transplant recipients, 14% received organs from donors with unrecognized bloodstream infections (BSI); while overall one-year survival was similar to recipients from uninfected donors, those receiving organs from donors with multidrug-resistant (MDR) BSI—particularly carbapenem-resistant Enterobacterales—had significantly lower survival, highlighting that MDR donor infections can adversely impact outcomes despite prompt treatment of donor-derived infections.

**Bone and Joint Infections**

- [Piquart L, Goutelle S, Maillard M, Boisset S, Carricajo A, Tristan A, et al. Temocillin: a therapeutic alternative in bone and joint infections due to third generation cephalosporin-resistant Enterobacterales? J Antimicrob Chemother. 2026 Apr;81(4):dkag105. doi:10.1093/jac/dkag105](https://ad-id.co/4s7Bzkq)
  - Summary: Temocillin demonstrated high in vitro activity against third-generation cephalosporin-resistant Enterobacterales from bone and joint infections—including ESBL producers and AmpC hyperproducers—with 92.3% susceptibility; however, optimal pharmacokinetic/pharmacodynamic target attainment depends on both the isolate’s MIC and the patient’s renal function, highlighting the need for dosage adjustments to maximize efficacy.

**Skin and Soft Tissue Infections**

- [Dulcey M, DeBord KM, Bell ME, Murray MT, Szewc AM, Livingston K, et al. Haematospirillum jordaniae infections after recreational exposure to river water, Pennsylvania, USA, 2020. Emerg Infect Dis. 2025 Nov;31(11):2073-2079. doi: 10.3201/eid3111.241586](https://ad-id.co/4dDnVC3)
  - Summary: Four cases of Haematospirillum jordaniae infection were identified in 2020 in south-central Pennsylvania, USA, linked to leg injuries sustained during recreational river water activities. The bacterium was later detected in river samples collected in 2024 from locations reported by patients. Initial emergency department visits did not identify the causative agent, which was only confirmed months after discharge. Although considered rare, the true incidence of H. jordaniae infections is unknown, and understanding its environmental ecology and seasonality could improve public health guidance and clinician awareness.
- [Napolitano LM, Biffl WL, Costantini TW, Diaz JJ, Inaba K, Livingston DH, et al. Evidence-based, cost-effective management of necrotizing soft tissue infection: an algorithm of the Journal of Trauma and Acute Care Surgery emergency general surgery algorithms work group. J Trauma Acute Care Surg. 2026 Apr;100(4):542–548. doi:10.1097/TA.0000000000004943](https://ad-id.co/4bMWmmU)
  - Summary: The Journal of Trauma and Acute Care Surgery Emergency General Surgery algorithms work group developed a bedside reference algorithm for the initial evaluation and management of necrotizing soft tissue infection (NSTI) in the emergency setting. NSTI is a rare but rapidly progressive and potentially life- and limb-threatening infection, with mortality rates of 20–25% and high resource utilization, including prolonged ICU and hospital stays. Early recognition and prompt surgical debridement are critical for improving outcomes, though incidence and causative pathogens—such as group A Streptococcus—vary regionally, and readmission rates remain high. The algorithm provides evidence-based guidance but must be adapted to clinical judgment and local protocols.

**Sexually Transmitted Infections**

- [Menza TW, Berzkalns A, Cannon CA, Balkus J, Kerani RP, Dombrowski JC, et al. The population-level impact of doxycycline post-exposure prophylaxis on syphilis in King County, WA: an interrupted time series analysis. Clin Infect Dis. 2026 Mar 26; doi:10.1093/cid/ciag209](https://ad-id.co/4sfKHDG)
  - Summary: After doxycycline post-exposure prophylaxis (doxy-PEP) was implemented in King County, WA in March 2023, overall syphilis diagnoses declined by 52%, with substantial reductions among cisgender men (-53%), cisgender women (-47%), and transgender/nonbinary people (-33%). However, syphilis cases in pregnant persons and congenital syphilis continued to rise, indicating that doxy-PEP’s population-level impact does not extend to these groups.
  - **Editorial Commentary:**
    [Bolan RK. Doxycycline prophylaxis for syphilis: proceed with caution. Clin Infect Dis. 2026 Mar 26; doi:10.1093/cid/ciag210](https://ad-id.co/4dl5ZMb)
- [Barbee LA, Zhang S, Golden MR, Khosropour CM, Soge OO, Manhart LE. The natural history of Mycoplasma genitalium at the pharynx and rectum in a cohort of men who have sex with men: prevalence, incidence, duration and symptomatology. J Infect Dis. 2026 Mar 20; doi:10.1093/infdis/jiag173](https://ad-id.co/41HATak)
  - Summary: In a cohort of men who have sex with men, rectal and pharyngeal *Mycoplasma genitalium* infections were common, mostly asymptomatic, and persistent—rectal infections often lasting close to a year and pharyngeal infections around three months. The high asymptomatic rates and prolonged duration suggest routine testing at these sites may offer limited individual benefit.
- [Mukherjee A, Blomqvist SOP, Helekal D, Das AA, Palace SG, Grad YH, et al. Genetic background modulates zoliflodacin and gepotidacin cross-resistance and fitness in Neisseria gonorrhoeae. J Infect Dis. 2026 Mar 19; doi:10.1093/infdis/jiag174](https://ad-id.co/4dfO5KP)
  - Summary: In ciprofloxacin-resistant *Neisseria gonorrhoeae*, the gyrBD429N mutation conferred cross-resistance to gepotidacin in some strains, with its effects on fitness and resistance influenced by the strain’s genetic background, particularly the presence of parCD86N. These results highlight the need to consider genetic context when introducing and monitoring new topoisomerase inhibitors like zoliflodacin and gepotidacin.
- [Michalow J, Cori A, Kimani J, Bhattacharjee P, Boily M-C, Imai-Eaton JW, et al. Optimal deployment of gonorrhoea point-of-care tests: modeling the potential impact of diagnostic confirmation testing and screening strategies across five priority populations in Kenya. J Infect Dis. 2026 Mar 17; doi:10.1093/infdis/jiag162](https://ad-id.co/4bW7SfM)
  - Summary: Modeling gonorrhea transmission in Kenya suggests that, under limited POCT availability, prioritizing diagnostic confirmation for symptomatic individuals—particularly pregnant women—prevents the most morbidity and is more efficient than broad screening, supporting WHO guidance to strengthen etiologic diagnosis within syndromic care.

**Respiratory Tract Infections**

- [Tam KKG, Suster CJS, Fong W, Golubchik T, Sivalingam V, Jeoffreys N, et al. Genomic surveillance reveals emergence and spread of macrolide-resistant Mycoplasma pneumoniae in Australia during the 2023–2024 epidemic. J Infect Dis. 2026 Mar 21; doi:10.1093/infdis/jiag163](https://ad-id.co/4deATG1)
  - Summary: Using targeted metagenomic sequencing, the study revealed a genetically diverse population of *Mycoplasma pneumoniae* in Australia, with 13% macrolide-resistant strains linked to higher healthcare utilization; while macrolides remain largely effective, ongoing MRMP surveillance is critical to guide treatment and stewardship.
- [Azoulay E, McEvoy C, Castro P, Ait Hssain A, Taccone FS, Myatra SN, et al. Epidemiology, ventilation, and outcomes of acute respiratory failure in immunocompromised patients from 103 intensive care units in 26 countries: a retrospective observational study. Lancet Respir Med. 2026 Mar 16; doi:10.1016/S2213-2600(26)00046-9](https://ad-id.co/41Cj6Bv)
  - Summary: In a large international cohort of 9854 immunocompromised ICU patients with acute hypoxaemic respiratory failure, 30-day mortality was 47.3%, with higher risk associated with older age, comorbidities, coma, invasive fungal infection, unidentified ARF cause, and organ support needs, while solid organ transplantation, connective tissue disease, higher oxygenation, and high-flow nasal oxygen were protective, highlighting factors to guide timely clinical decisions and management.
- [Zhang X, Z T. Interleukins in community-acquired pneumonia: from biomarkers to precision medicine. Front Immunol. 2026 Feb 24;17:1774731. doi:10.3389/fimmu.2026.1774731](https://ad-id.co/4s5pUT4)
  - Summary: This review highlights the roles of key interleukins in community-acquired pneumonia, categorizing them by pro-inflammatory, anti-inflammatory, dual-action, and emerging functions, and emphasizes integrating cytokine profiles with organ dysfunction, multi-omics, and AI-driven approaches to enable precision medicine and improve severity assessment and management in CAP.

**Infections in Children**

- [Weiss SL, Peters MJ, Oczkowski SJW, Belley-Cote E, Buysse C, Choong KLM, et al. Surviving Sepsis Campaign International Guidelines for the Management of Sepsis and Septic Shock in Children 2026. Intensive Care Med. 2026 Mar 23; doi:10.1007/s00134-026-08360-2](https://ad-id.co/3NUsInS)
  - Summary: An international panel of experts updated evidence-based guidelines for managing children with sepsis or septic shock, issuing 61 statements—including strong, conditional, and good practice recommendations—highlighting that most recommendations are based on low-quality evidence, with only a few supported by moderate or high-certainty data.
- [Lieu TA, Alexander JT, Seymour CW. Caring for Pediatric Patients With Sepsis. JAMA. 2026 Mar 26; doi:10.1001/jama.2026.3800](https://ad-id.co/4s0ZxgT)
  - Summary: The 2026 Surviving Sepsis Campaign International Guidelines for the Management of Sepsis and Septic Shock in Children update the 2020 recommendations, providing evidence- and expert-informed guidance for children from ≥37 weeks gestational age to <18 years. Key recommendations include routine infectious disease consultation for documented bloodstream infections, blood lactate measurement for tissue perfusion assessment, and use of cardiac and lung POCUS to guide resuscitation. In resource-limited settings, fluid boluses are strongly discouraged except in severe hypotension, and conservative oxygen targets (SpO₂ 88–92%) are suggested for intubated children. The guidelines emphasize early, timely antimicrobial therapy, ongoing hemodynamic assessment, and high-volume hemofiltration when kidney support is required. Of 61 statements, 5 are strong recommendations, 24 conditional, and 10 good practice statements, highlighting that most recommendations remain based on low- to very low-certainty evidence despite updated research and expert consensus.
- [Founou LL, Founou RC, Charani E, Gwanyama N, Dramowski A, Feasey N. Neonatal sepsis in Africa: what are we missing? Lancet Microbe. 2026 Mar 18; doi:10.1016/j.lanmic.2026.101374](https://ad-id.co/4s0eOyB)
  - Summary: Neonatal sepsis continues to be a critical public health challenge in Africa, posing a major barrier to achieving the UN Sustainable Development Goal target of reducing neonatal mortality to 12 per 1000 livebirths. Progress is hindered not only by diagnostic, treatment, and prevention gaps, but also by sociocultural norms, fragmented health systems, inequities in care, and low political commitment. Antimicrobial-resistant infections exacerbate the crisis, placing neonates at disproportionate risk. Without urgent, context-appropriate interventions, Africa will continue to bear the highest burden of preventable neonatal deaths. A shift toward a community-centred One Health approach, integrating biomedical, environmental, and sociocultural factors, along with coordinated global and local leadership, is essential to accelerate progress and reduce neonatal mortality.
- [St Peter SD, Ampofo K, Brogan T, Cabana MD, Espinosa C, Florin TA, et al. 2026 Clinical Practice Guideline Update by the Infectious Diseases Society of America and the Pediatric Infectious Diseases Society on the Management of Community-acquired Pneumonia in Infants and Children Older than 3 Months of Age: The Use of tPa and DNase or tPa Alone for Fibrinolysis. Clin Infect Dis. 2026 Mar 17; doi:10.1093/cid/ciag191](https://ad-id.co/4bYlg3l)
  - Summary: This paper is part of a larger clinical practice guideline on the diagnosis and management of parapneumonic effusion and empyema in children. In this report, the panel provides recommendations for intrapleural fibrinolysis with tissue plasminogen activator (tPA) alone over tPA and dornase alfa (DNase) in children (3 months to 18 years) with complicated parapneumonic effusion and empyema. The panel’s recommendations are based upon evidence derived from systematic literature reviews and adhere to a standardized methodology for rating the certainty of evidence and strength of recommendation according to the GRADE (Grading of Recommendations Assessment, Development, and Evaluation) approach.
- [St Peter SD, Ampofo K, Brogan T, Cabana MD, Espinosa C, Florin TA, et al. 2026 Clinical Practice Guideline Update by the Infectious Diseases Society of America and the Pediatric Infectious Diseases Society on the Management of Community-acquired Pneumonia in Infants and Children Older than 3 Months of Age: The Choice of Chest Tube Size. Clin Infect Dis. 2026 Mar 17; doi:10.1093/cid/ciag190](https://ad-id.co/4c55V10)
  - Summary: This paper is part of a larger clinical practice guideline on the diagnosis and management of parapneumonic effusion and empyema in children. In this paper, the panel provides recommendations on the appropriate size of thoracostomy tube for drainage. The panel’s recommendations are based upon evidence derived from systematic literature reviews and adhere to a standardized methodology for rating the certainty of evidence and strength of recommendation according to the GRADE (Grading of Recommendations Assessment, Development, and Evaluation) approach.
- [St Peter SD, Ampofo K, Brogan T, Cabana MD, Espinosa C, Florin TA, et al. 2026 Clinical Practice Guideline Update by the Infectious Diseases Society of America and the Pediatric Infectious Diseases Society on the Management of Community-Acquired Pneumonia in Infants and Children Older than 3 Months of Age: The Use of Chest Ultrasound in Children with Parapneumonic Effusion. Clin Infect Dis. 2026 Mar 16; doi:10.1093/cid/ciag187](https://ad-id.co/4bVnD6H)
  - Summary: This paper is part of a larger clinical practice guideline on the diagnosis and management of parapneumonic effusion and empyema in children. In this paper, the panel provides recommendations for the role of chest ultrasound to evaluate parapneumonic effusion and empyema. The panel’s recommendations are based upon evidence derived from systematic literature reviews and adhere to a standardized methodology for rating the certainty of evidence and strength of recommendation according to the GRADE (Grading of Recommendations Assessment, Development, and Evaluation) approach.
- [St Peter SD, Ampofo K, Brogan T, Cabana MD, Espinosa C, Florin TA, et al. Clinical Practice Guideline by the Infectious Diseases Society of America and the Pediatric Infectious Diseases Society: 2026 Guideline Update on The Management of Community-Acquired Pneumonia in Infants and Children Older than 3 Months of Age. Clin Infect Dis. 2026 Mar 16; doi:10.1093/cid/ciag186](https://ad-id.co/3Q920sm)
  - Summary: As the first part of an update to the clinical practice guideline on the management of community-acquired pneumonia in infants and children older than 3 months of age, we present six updated recommendations. The updated recommendations span the characterization and management of pneumonia with parapneumonic effusion. The panel’s recommendations are based on evidence derived from systematic literature reviews and adhere to a standardized methodology for rating the certainty of evidence and strength of recommendation according to the GRADE (Grading of Recommendations Assessment, Development, and Evaluation) approach.
- [Chung CH, Barash JR, Castonguay JL, Penzel-McNamara C, Lee K, Read JS, et al. Multistate Infant Botulism Outbreak Associated with Powdered Infant Formula. N Engl J Med Evid. 2026 Feb 25;5(4): doi:10.1056/EVIDpha2600020](https://ad-id.co/3PxKXjL)
  - Summary: This report details the identification of an outbreak of infant botulism linked to powdered infant formula in the United States. In October 2025, the Infant Botulism Treatment and Prevention Program, California Department of Public Health (IBTPP-CDPH) noted common powdered infant formula exposure among three neonates with suspected infant botulism. Subsequent laboratory analyses at CDPH identified Clostridium botulinum type A from an open container of ByHeart powdered infant formula associated with one infant with infant botulism. As of December 10, 2025, 51 suspected or confirmed infant botulism cases with exposure to ByHeart powdered infant formula had been identified across 19 states. All ByHeart powdered infant formula has been recalled nationwide.
- [Khan AZ, Khan FZ, Sajid A, Khan ZZ, Murphy KJ, Mazer M. A 9-Year-Old Girl with Persistent Fevers and Lethargy. N Engl J Med Evid. 2026 Mar 24;5(4): doi:10.1056/EVIDmr2400406](https://ad-id.co/3NxRcn2)
  - Summary: This report examines the case of a 9-year-old girl who presented to the emergency department with prolonged fevers and malaise. Using questions, physical examination, and testing, an illness script for the presentation emerges. As the clinical course progresses, the differential is refined until a diagnosis is made.
- [Caddy SL, van Dorp L, Swadling L, Wang-Koh Y, Houldcroft CJ. Germ factories or immune boot camps? Infection and immunity in childcare settings. Clin Microbiol Rev. 2026 Mar 18; doi:10.1128/cmr.00253-25](https://ad-id.co/4c2gORb)
  - Summary: Childcare outside the home is common in high-income countries and is associated with higher incidence of infectious diseases for children and their household members. This review examines how the age at which children start childcare interacts with the maturation of cellular and humoral immunity, drawing on maternal antibody dynamics, seroepidemiology, cohort studies, and outbreak reports. It summarizes typical infections in early life, including gastrointestinal, respiratory, and rash-forming illnesses, and considers the additional impact of childcare on pathogen transmission. The economic and personal effects of these infections are discussed, as well as interventions such as vaccination programs. The review also explores a potential trade-off between early-life infections and later school-age illness, concluding that the high burden of infection in young children is biologically normal but its collective impact is often underestimated.

**Mycobacterial Infections**

- [Nguyen TM, MacLean ELH, Zhang X, Georghiou SB, Xia H, Beardsley J, et al. Molecular diagnostic tests for isoniazid-resistant tuberculosis: a scoping review. Lancet Microbe. 2026 Mar 20; doi:10.1016/j.lanmic.2026.101362](https://ad-id.co/4bXJUkq)
  - Summary: Molecular diagnostics for isoniazid-resistant tuberculosis have expanded rapidly and generally meet performance targets, but their complexity, cost, and limited accessibility—especially in low-resource settings—continue to hinder widespread implementation despite the critical need for early detection.
- [Lam WKJ, Chan KKP, Wang G, Lai CKC, Kang G, Chan C, et al. Sequencing of pleural fluid and plasma for tuberculous pleuritis. N Engl J Med Evid. 2026 Mar 24;5(4): doi:10.1056/EVIDoa2500237](https://ad-id.co/3NzBFmH)
  - Summary: Targeted sequencing of pleural fluid with masked Mycobacterium tuberculosis genomic alignment demonstrated markedly higher sensitivity (97.1% vs 47.1%) and near-perfect diagnostic accuracy for tuberculous pleuritis compared with culture, offering a highly effective approach for detecting this paucibacillary disease.
  - **Editorial Commentary:**[AbdelHalim HA, AboElNaga HH. Unlocking the diagnostic challenge of tuberculous pleural effusions. N Engl J Med Evid. 2026 Mar 24;5(4): doi:10.1056/EVIDe2500329](https://ad-id.co/41D3KN6)
- [Smith JP, O’Connor S, Date A, Moonan PK. Tuberculosis cases and deaths averted by PEPFAR. N Engl J Med. 2026 Mar 24; doi:10.1056/NEJMc2506284](https://ad-id.co/4bY5O76)
  - Summary: Since 2003, PEPFAR has averted an estimated 11 million tuberculosis cases and 2.1 million deaths among people with HIV, highlighting the major impact of integrated HIV and tuberculosis interventions on global disease control.
- [Visek C, Dalmat RR, Nalutaaya A, Erisa KC, Biché P, Stein G, et al. Prevalence and predictors of tuberculosis in adults and adolescents with sputum trace Ultra results in 2 high-burden clinical settings. Clin Infect Dis. 2026 Mar 20; doi:10.1093/cid/ciag019](https://ad-id.co/4bVp3OR)
  - Summary: Among patients with trace-positive Xpert MTB/RIF Ultra results, only about 20–30% had microbiologically confirmed tuberculosis, but nearly half were started on treatment, suggesting most should be treated when further evaluation is limited, while selected low-risk patients may be safely observed with follow-up.
  - **Editorial Commentary:**[Sossen B, Martinson N. “Trace-MTB” on sputum Xpert Ultra: new evidence for interpretation in high-burden settings. Clin Infect Dis. 2026 Mar 20; doi:10.1093/cid/ciag018](https://ad-id.co/41Cqr3U)
- [Selva Kumar D S, Somarkutty N, Hariram Prasad D J, Gautam P, Nagaraj V, Karthik R, et al. Role of low-dose infliximab for inflammatory complications of central nervous system tuberculosis: a retrospective cohort study. Clin Infect Dis. 2026 Mar 20; doi:10.1093/cid/ciaf573](https://ad-id.co/4bRAuqE)
  - Summary: In 20 patients with severe central nervous system tuberculosis, low-dose infliximab (5 mg/kg) as adjunctive therapy resulted in 60% disability-free survival and 75% showing meaningful clinical improvement, comparable to high-dose reports, highlighting the need for randomized trials to optimize dosing.
  - **Editorial Commentary:**[Thwaites GE. Targeting inflammation in tuberculous meningitis treatment. Clin Infect Dis. 2026 Mar 20; doi:10.1093/cid/ciaf574](https://ad-id.co/4s3VuR6)
- [van Riet E, Corleis B, Giersing BK, Hatherill M, Burhan E, Jassat W, et al. Accelerating research and development of new vaccines against tuberculosis: 5-year progress on the global roadmap. Lancet Infect Dis. 2026 Mar 18; doi:10.1016/S1473-3099(26)00019-8](https://ad-id.co/4bXT8Nw)
  - Summary: Since the 2021 global tuberculosis vaccine R&D roadmap, the pipeline has diversified with several candidates in phase 3 trials, but clinical trial numbers remain small, development challenges persist, and insufficient investment and procurement commitments limit progress, highlighting the need for strategic coordination, diversified funding, and planning for effective vaccine implementation and uptake.
- [Vuyyuru SK, Singh U, Das P, Basant S, Singh V, Sinha SK, et al. Association of Mycobacterium Avium Paratuberculosis with Crohn's disease: a large multicenter study from a tuberculosis-endemic region. Clin Infect Dis. 2026 Jan 5; doi:10.1093/cid/ciaf738](https://ad-id.co/3PxKT3v)
  - Summary: In a multicenter observational study in India including 889 participants, Mycobacterium avium subspecies paratuberculosis (MAP) was more frequently detected in patients with Crohn's disease (CD) than in controls, intestinal tuberculosis, or ulcerative colitis, particularly by serology, tissue PCR, and solid biopsy culture, suggesting an association between MAP and CD and supporting further research into its potential causal role and therapeutic targeting.
- [Cerqueira-Silva T, Boaventura VS, Paixão ES, Sanchez M, Leyrat C, Ranzani O, et al. Long-term risk of death after tuberculosis diagnosis and treatment. Nat Med. 2026 Mar 19; doi:10.1038/s41591-026-04294-w](https://ad-id.co/4c0qeMY)
  - Summary: A nationwide Brazilian cohort study found that individuals diagnosed with or treated for tuberculosis had significantly elevated long-term mortality up to 14 years later, including deaths from cancer, cardiovascular, endocrine, respiratory, and external causes, emphasizing the need for prolonged monitoring of TB survivors.
- [Miranda-Hernandez S, Kumar M, Henderson A, Graham E, Tan X, Taylor J, et al. CD8+ T cells sustain vaccination-induced immunity against dissemination of contained tuberculosis in immunosuppressed hosts. Nat Commun. 2026 Mar 24; doi:10.1038/s41467-026-70911-4](https://ad-id.co/418TrQK)
  - Summary: Using a mouse model of lymphatic latent Mycobacterium tuberculosis infection, researchers found that vaccination with BCG or recombinant BCG prevents reactivation and dissemination even without CD4+ T cells, with CD8+ T cells mediating protection, highlighting potential strategies for managing latent TB in immunocompromised individuals.
- [Chung TK, Yang E, Shin M, Solans BP, Zhou X, Hwang S, et al. Pharmacokinetics and dose optimization of ethambutol in children on first-line antituberculosis therapy: an individual patient data meta-analysis. J Infect Dis. 2026 Mar 30; doi:10.1093/infdis/jiag194](https://ad-id.co/4bYlk35)
  - Summary: An individual patient data meta-analysis of 220 children with tuberculosis showed that current WHO-recommended ethambutol doses often result in suboptimal drug exposure, particularly in children under 25 kg, and proposed optimized weight-based dosing to improve efficacy while balancing toxicity and formulation considerations.
- [Zhang S, Qi J, Zheng J, Qin Y, Yang J, Li X, et al. National and subnational burden of XDR-TB in China, 1990-2023: long-term trends, regional disparities, and projections, 2024-2050. J Infect. 2026 Mar 15; doi:10.1016/j.jinf.2026.106728](https://ad-id.co/4dkuIjO)
  - Summary: Analysis of 1990–2023 data in China shows that extensively drug-resistant tuberculosis (XDR-TB) is rising in incidence, prevalence, and mortality, with marked geographic disparities—Xinjiang and Tibet bear the highest burdens—and highlights the need for targeted interventions, especially for high-risk regions and older populations.
- [Purohit D, van Wijk R, Kafeero P, Kibengo F, Zimmerman M, D'artois V, et al. Impact of high-dose rifampicin on linezolid pharmacokinetics in tuberculous meningitis. Open Forum Infect Dis. 2026 Mar 27; doi:10.1093/ofid/ofag154](https://ad-id.co/4s8znsK)
  - Summary: High-dose rifampicin significantly increases the clearance of linezolid in adults with tuberculous meningitis, reducing plasma and cerebrospinal fluid concentrations, but twice-daily dosing of linezolid can maintain therapeutic levels, highlighting the need to optimize dosing strategies when these drugs are co-administered to ensure effective treatment.
- [Funauchi A, Hashimoto K, Fukushima K, Matsumoto Y, Hamada N, Hara R, et al. Gastric aspirate isolate demonstrates strain-level concordance with sputum isolate in nontuberculous mycobacterial pulmonary disease. Open Forum Infect Dis. 2026 Mar 27; doi:10.1093/ofid/ofag175](https://ad-id.co/4saVi2x)
  - Summary: Nontuberculous mycobacteria isolated from gastric aspirate show over 85% strain concordance with sputum isolates, indicating a pulmonary origin and suggesting that gastric aspirate could serve as a useful supplementary specimen for diagnosing NTM pulmonary disease.
- [Moyo RC, Okango E, Bolton L, Otto M, Blose N, Sereo T, et al. Effect of the scale-up of dolutegravir on retention in care, risk of developing tuberculosis and viral load suppression among people living with HIV: analysis of routine HIV clinical data in rural KwaZulu-Natal, South Africa (2019–23). Open Forum Infect Dis. 2026 Mar 19; doi:10.1093/ofid/ofag156](https://ad-id.co/4dl60jm)
  - Summary: In a cohort of 69,919 people living with HIV in rural KwaZulu-Natal, South Africa, approximately 70% transitioned to dolutegravir-containing regimens over four years, and DTG use was associated with higher viral load suppression, better retention in care, and a lower risk of developing tuberculosis, supporting the continued rollout of DTG-based therapy and the need for ongoing programmatic monitoring.

**Fungal Infections and Antifungal Agents**

- [Muthu V, Sehgal IS, Agarwal R. Treatment of pulmonary mucormycosis: current concepts. Expert Rev Anti Infect Ther. 2026 Jan 29; doi:10.1080/14787210.2026.2622695](https://ad-id.co/3NAYB53)
  - Summary: Pulmonary mucormycosis is a rapidly progressive, high-mortality fungal infection, and optimal management involves early recognition, aggressive host factor optimization such as glycemic control and immunosuppression reduction, prompt induction with liposomal amphotericin B followed by oral triazole maintenance, careful consideration of surgery, and multidisciplinary care, while combination antifungal therapy should be reserved for severe or refractory cases, and future research should focus on evaluating treatment strategies, host-directed therapies, emerging antifungals, and adjunctive modalities across special populations.
- [Gigante V, Alm RA, Rocke T, Melchiorri D, Cameron AM, et al. WHO assessment of the preclinical antifungal pipeline: evaluating innovation and preparedness in the face of emerging fungal threats. Lancet Microbe. 2026 Mar 27; doi:10.1016/j.lanmic.2025.101331](https://ad-id.co/4djjNH9)
  - Summary: Invasive and mucosal fungal infections are a growing global health threat, particularly among immunocompromised populations, with rising incidence, drug resistance, and climate-related influences, while current antifungal treatments are limited in spectrum and safety, prompting the WHO to identify priority fungal pathogens and stimulate research and development; preclinical and clinical pipelines are emerging with novel agents such as fosmanogepix and olorofim, but sustainability, safety evaluation, and alignment with global health needs remain critical to ensure effective, broad-spectrum, and equitable therapies for these high-burden infections.
- [Kroustali V, Pournaras S, Meletiadis J. Can fluconazole be used to treat non-resistant Candida (Candidozyma) auris infections? Preclinical PK/PD data from a Galleria mellonella infection model. J Infect Dis. 2026 Mar 20; doi:10.1093/infdis/jiag182](https://ad-id.co/4bW7NZw)
  - Summary: Fluconazole, despite widespread resistance in Candida auris, showed similar in vivo activity to Candida albicans in a Galleria mellonella model, with isolates having MICs ≤8 mg/L potentially treatable at 1,200 mg/day, suggesting that select non-resistant C. auris infections could respond to fluconazole, though clinical studies are needed to confirm efficacy.
- [Tan M, Guo Z, Wang Y, Xu X, Cao W, Liu Z, et al. Identification of Aspergillus at section and species levels by artificial intelligence-based microscopic morphology image recognition. J Clin Microbiol. 2026 Feb 27; doi:10.1128/jcm.00012-26](https://ad-id.co/4dOgury)
  - Summary: FungalNet, a deep learning model based on ResNet-50 and Focal Loss, accurately identified Aspergillus species and sections from 11,689 high-resolution microscopic images with overall accuracies of 98.45% and 97.85%, demonstrating promise for rapid, reliable Aspergillus diagnosis in clinical laboratories and potential integration into routine workflows after further validation.
- [Godet C, Joste V, Frat J-P, Khalil A, Bunel V, Goletto T, et al. Non-fumigatus Aspergillus-associated pulmonary events: a diagnostic challenge. J Clin Microbiol. 2026 Mar 18; doi:10.1128/jcm.00163-26](https://ad-id.co/4bZ9CFk)
  - Summary: In a retrospective study of 497 patients with respiratory samples positive for non-fumigatus Aspergillus species, 10.5% experienced pulmonary events, mostly colonization, while nearly one-third had clinically significant pulmonary disease, with Aspergillus niger, A. flavus, and A. nidulans being most frequent, and low positivity rates across conventional diagnostic tests highlight the need for repeated sampling and the limitations of assays designed primarily for A. fumigatus.
- [Bitterman R, Kus JV, Verma G, Kopp A, Husain S, Kwong JC, et al. Incidence and outcomes of Candida bloodstream infection in solid organ transplant recipients. JAMA Netw Open. 2026 Mar 17;9(3):e261467. doi:10.1001/jamanetworkopen.2026.1467](https://ad-id.co/4dl656E)
  - Summary: In a population-based cohort study of 10,249 solid organ transplant recipients in Ontario, Canada, candidemia occurred in 1.67% of patients, with lung transplant recipients at the highest risk, and was associated with high mortality—39.3% at 30 days and 47.4% at 90 days—with both fluconazole-susceptible and fluconazole-resistant Candida infections significantly increasing the risk of death, highlighting the need for targeted prevention and early intervention strategies in this vulnerable population.

**Virulence**

- [Snaith AE, van der Putten B, Bril-Keijzers W, Hall RJ, Dunn SJ, van Schaik W, et al. Lineage dynamics of invasive Escherichia coli isolates in the Netherlands from 1975 to 2021: a retrospective longitudinal genomic analysis. Lancet Microbe. 2026 Mar 18; doi:10.1016/j.lanmic.2025.101300](https://ad-id.co/4s5CHEW)
  - Summary: The SENTINEL study longitudinally analyzed 1,790 invasive Escherichia coli isolates, mainly from neonates in the Netherlands between 1975 and 2021, revealing a highly dynamic population with shifts in dominant lineages and virulence factors, only 58.8% of isolates expressing the K1 capsule, and no clear influence from antimicrobial resistance, highlighting the importance of host-pathogen interactions, immune selection pressures, and the need for ongoing genomic surveillance to guide interventions and cautioning against generalizing findings from a single cohort to other populations.

**Microbiome**

- [Baldanzi G, Larsson A, Sayols-Baixeras S, Dekkers KF, Hammar U, Nguyen D, et al. Antibiotic use and gut microbiome composition links from individual-level prescription data of 14,979 individuals. Nat Med. 2026 Mar 11; doi:10.1038/s41591-026-04284-y](https://ad-id.co/4bSqPjx)
  - Summary: This study analyzed fecal metagenomes from 14,979 adults in Sweden and found that oral antibiotic use, even 4–8 years prior, was associated with long-lasting alterations in gut microbiome composition, with the strongest reductions in species diversity observed within one year of use and clindamycin, fluoroquinolones, and flucloxacillin contributing most to changes, highlighting the enduring impact of antibiotics on gut microbial communities.
- [Spottiswoode N, Neyton LP, Mick E, Kalantar KL, Hao S, Lydon EC, et al. Host–Microbe Multiomic Profiling Predicts Mortality in Sepsis. Am J Respir Crit Care Med. 2026 Jan;212(1):95–104. doi:10.1164/rccm.202410-1996OC](https://ad-id.co/4bRU9GV)
  - Summary: This study of 321 critically ill adults with sepsis found that mortality was linked to host immune dysregulation, including increased neutrophil degranulation gene expression, decreased T-cell signaling, and elevated IL-8, as well as microbial factors such as higher bacterial load and dominance, and demonstrated that integrated host–microbe metagenomic and host transcriptomic classifiers predicted sepsis mortality more accurately than conventional scoring systems, highlighting the combined influence of host and microbial factors on outcomes in critical illness.
  - **Editorial Commentary:**[Barnett CR, Bos LDJ, Segal LN. Microbes Meet Host: The Next Frontier in Sepsis Classification. Am J Respir Crit Care Med. 2026 Jan;212(1):8–10. doi:10.1164/rccm.202507-1590ED](https://ad-id.co/3NxR8DO)

**Diagnostics**

- [Gigante V, Murtagh M, Bachmann TT, Rocke T, Trainor BW, Poutanen SM, et al. Diagnostics for priority bacterial pathogens: global gaps and research needs for curbing antimicrobial resistance in low-resource settings. Lancet Microbe. 2026 Mar 20; doi:10.1016/j.lanmic.2026.101385](https://ad-id.co/4c55Z0K)
  - Summary: Antimicrobial resistance (AMR) poses a growing global health threat, particularly in low- and middle-income countries, with an estimated 39.1 million direct deaths projected between 2020 and 2050 if unaddressed, and effective bacterial diagnostics are crucial for pathogen detection, antimicrobial susceptibility testing, and antibiotic stewardship, yet access to appropriate diagnostics remains limited in LMICs; this Review outlines the current commercial and pipeline in-vitro bacterial diagnostic landscape, evaluates phenotypic and non-phenotypic approaches, highlights infrastructure challenges that restrict testing to higher-level laboratories, and emphasizes WHO priorities for the next 3–5 years to promote innovation, equitable access, and development of simple, affordable, and decentralised solutions for reliable identification and resistance testing of priority bacterial pathogens.
- [Koroki T, Fujii M, Kotani Y, Yaguchi T, Shibata T, Hirata C, et al. Contamination of blood cultures drawn from arterial catheters versus venipuncture or venous catheters in critically ill patients: a systematic review and meta-analysis. Clin Infect Dis. 2026 Mar 15;82(3):446–452, doi:10.1093/cid/ciaf260](https://ad-id.co/4dg8tvn)
  - Summary: Arterial catheter–drawn blood cultures in critically ill patients have contamination rates similar to venipuncture-drawn cultures and may be lower than venous catheter–drawn cultures, suggesting they could be a safe alternative for blood culture collection, though the certainty of evidence is low.
- [Shorr AF, Kollef MH, Wunderink RG, Jauregui-Peredo LE, Bernard AC, Kim HK, et al. Diagnostic performance of point-of-care immunoassay measurements of pancreatic stone protein for sepsis detection in ICU patients: a prospective, multicenter, biomarker-blinded study. Crit Care Med. 2026 Mar 4; doi:10.1097/CCM.0000000000007087](https://ad-id.co/4dg8tLT)
  - Summary: In a multicenter ICU study of 466 adults, a rapid point-of-care pancreatic stone protein (PSP) assay identified sepsis within the first three days with 74.2% sensitivity, 67.8% specificity, and 71.0% accuracy; combining PSP with C-reactive protein (CRP) improved specificity to 95.2%, supporting PSP’s broad applicability as an early sepsis biomarker and the benefit of combined biomarker strategies for timely detection.

**Improving Clinical Research**

- [Zumbo G, Beltrami M, Bravo-Ferrer JM, Gutiérrez-Gutiérrez B, Rodríguez-Baño J, et al. Methodological quality assessment tool for observational studies comparing treatment effectiveness in infectious diseases: a Delphi consensus and application to studies on bloodstream infections due to carbapenem-resistant Enterobacterales. Lancet Infect Dis. 2026 Mar 23; doi:10.1016/S1473-3099(26)00058-7](https://ad-id.co/4dTaHku)
  - Summary: The ASSURE-ID questionnaire, developed via a three-round Delphi process with 34 experts, provides a structured framework to assess methodological quality and bias in observational infectious disease studies; its application to 14 studies on bloodstream infections caused by carbapenem-resistant Enterobacterales revealed frequent shortcomings, including lack of protocol pre-registration, limited reporting guideline adherence, minimal handling of missing data, and inconsistent addressing of biases and confounders, underscoring the need for improved study rigor.
- [Gelman A, van Zwet E, Więcek W. FDA Draft Guidance for the Use of Bayesian Methods in Clinical Trials. JAMA. 2026 Mar 23; doi:10.1001/jama.2026.4178](https://ad-id.co/4dg8u2p)
  - Summary: The FDA draft guidance on Bayesian methods in clinical trials emphasizes the transparent use of prior information to improve trial efficiency and flexibility, particularly in pediatric and rare-disease studies. Unlike traditional frequentist approaches, the guidance allows informative priors to shift focus from strict type I error control to alternative success criteria based on risk-benefit analysis. It encourages borrowing strength from external data through meta-analysis and hierarchical modeling, while underscoring the need for clearly stated priors, prespecified data models, and simulation-based evaluation to ensure transparency, reproducibility, and regulatory rigor in trial design and interpretation.
- [Lee JJ, Harrell FE Jr, LaVange LM, Spiegelhalter DJ. Embracing Bayesian Methods in Clinical Trials: FDA’s Long-Awaited Draft Guidance. JAMA. 2026 Mar 23; doi:10.1001/jama.2026.4179](https://ad-id.co/4dg8uiV)
  - Summary: In January 2026, the FDA issued draft guidance endorsing Bayesian methods for therapeutic clinical trials, clarifying their regulatory acceptance and addressing prior misconceptions. Unlike traditional frequentist approaches that test a null hypothesis indirectly, Bayesian inference directly estimates the probability of treatment benefit by combining prior knowledge with observed data to generate a posterior distribution, offering a more clinically meaningful answer to whether a treatment works.
- [Evans SR, Fleming TR, Janes H, Dodd LE. Reflections on FDA Draft Guidance on Bayesian Methods in Trials—Protecting Scientific Integrity and Evidentiary Standards. JAMA. 2026 Mar 23; doi:10.1001/jama.2026.4175](https://ad-id.co/4dg8uzr)
  - Summary: The FDA’s draft guidance on Bayesian methods in clinical trials highlights the agency’s commitment to research integrity by providing clear standards and education on these approaches. While Bayesian methods have proven valuable in early-phase trials, diagnostics, and rare diseases, their use in late-phase confirmatory trials has been limited due to concerns that incorporating prior information could compromise randomization benefits, introduce subjective biases, and reduce robustness through strong or unverifiable assumptions.
- [Van Leeve J, Colacci M. Use of Deferred Consent in Randomized Clinical Trials. NEJM Evid. 2026 Mar 24;5(4): doi:10.1056/EVIDctw2400266](https://ad-id.co/4dg8uPX)
  - Summary: Deferred consent allows enrollment of critically ill or incapacitated patients into clinical trials before obtaining informed consent, enabling timely access to potentially beneficial interventions in emergency care settings. This review examines the ethical considerations, risks, and benefits of using deferred consent in randomized controlled trials, highlighting contexts where its use may be ethically acceptable.
- [Mahon N, Hays LMC, Coy E, Ainscough K, Burrell A, Gordon AC, et al. Views on consent approaches used in emergency and critical care research: a rapid, systematic review. Trials. 2026 Mar 11; doi:10.1186/s13063-026-09592-9](https://ad-id.co/4dg8v6t)
  - Summary: Obtaining informed consent in emergency and critical care research is often challenging, prompting the use of alternative consent models such as deferred consent, surrogate consent, or waived consent. This rapid systematic review of 145 studies from 26 countries found that alternative consent approaches are generally acceptable, especially when patients or relatives are involved in decision-making. Acceptability is influenced by prior research experience, illness severity, perceived risk, and intervention invasiveness. Pandemic contexts increased the need for flexible consent processes, while perspectives of underserved groups remain underreported and show no clear consensus.
- [Sidebotham D, Jones PM. Core concepts in statistics and research methods. Part 7: regression. BJA Educ. 2026 Mar 20; doi:10.1016/j.bjae.2026.02.004](https://ad-id.co/3NzBFDd)
  - Summary: Regression is a fundamental statistical framework that quantifies relationships between a response variable and one or more explanatory variables. Simple linear regression involves a continuous response and explanatory variables, while generalized linear models (GLMs) extend regression to accommodate diverse data types, including binary outcomes via logistic regression. Key skills include identifying response and explanatory variables, interpreting coefficients, choosing an appropriate model, and understanding regression assumptions. Regression originated with Sir Francis Galton’s 19th-century studies on heredity and regression to the mean, and today underpins most standard statistical analyses in both frequentist and Bayesian frameworks.
- [Augoustides JG. Platform trials in perioperative cardiothoracic and vascular practice—Is it time for development, deployment, and dissemination? J Vasc Anaesth. 2026 Feb 18; doi:10.1053/j.jvca.2026.02.024](https://ad-id.co/4dkfCuG)
  - Summary: Platform trials are a transformative approach to perioperative and critical care research, designed to evaluate multiple interventions under a single adaptive master protocol over extended periods. Unlike traditional randomized trials, they allow shared control groups, real-time addition or removal of treatment arms, and adaptation to evolving standards of care, resulting in significant cost and time efficiencies. Platform trials, such as the Healey trial for amyotrophic lateral sclerosis and MARLIN in low- and middle-income countries, demonstrate applicability across rare diseases, perioperative complications, and critical care settings. They support collaboration across trials, enhance patient-centered outcomes, and are particularly valuable in resource-limited environments, offering a scalable, flexible, and efficient solution to address inequities in perioperative morbidity and mortality.
- [Kimmoun A, de Jong A, Poole D. Ten methodological aspects to look for when critically reading randomized-controlled trials. What's New in Intensive Care. 2026 Mar 9; doi:10.1007/s00134-026-08372-y](https://ad-id.co/3Oe8Z2J)
  - Summary: This review identifies ten key methodological aspects for critically appraising randomized controlled trials (RCTs), focusing on trial trust, relevance, and design. The crucial points include checking the clinical question, population, outcomes, randomization, blinding, protocol adherence, data analysis, and potential for bias.
- [McIver WJ. Blinding in critical care trials with subjective outcomes: a note of caution. Acute Crit Care. 2026 Feb 5;41(1):186-188. doi:10.4266/acc.005525](https://ad-id.co/4dOgqrO)
  - Summary: Blinding in randomized controlled trials is critical to reducing bias, particularly when outcomes are subjective. Lack of blinding can exaggerate treatment effects, as illustrated by three recent acute and critical care trials: WATERFALL, NAVIGATE, and A2B. Key lessons include: (1) outcome assessors should be blinded when measures are subjective; (2) composite outcomes combining objective and subjective elements should be interpreted cautiously, especially if differences are driven by subjective components; and (3) when blinding is impossible, clinician-driven outcomes should be strictly protocolised and adherence reported, or hard outcomes like mortality should be used. These guiding principles help safeguard trial integrity and ensure more reliable interpretation of findings.
- [van Zwet EW, Harrell FE Jr, Senn SJ. An empirical assessment of the cost of dichotomization of the outcome of clinical trials. Stat Methods Med Res. 2026 Feb 5; doi:10.1002/sim.70402](https://ad-id.co/4dDnVSz)
  - Summary: Analysis of 21,435 randomized controlled trials from the Cochrane Database showed that 66% used binary outcomes, which have larger sample sizes, larger standard errors, and fewer statistically significant results than continuous outcomes. Many binary outcomes result from dichotomizing continuous variables, leading to avoidable information loss and inefficiency. On average, only about 60% of information is retained after dichotomization. The authors provide methods and tools to estimate information loss and adjust sample size calculations, advocating for “model continuously but interpret dichotomously” to improve trial efficiency, reduce participant burden, and maintain statistical power.
- [Beltran J, Etxeandia-Ikobaltzeta I, Piggott T, Akl EA, Mustafa RA, Hazlewood G, et al. New GRADE Evidence-to-Decision Framework for Pairwise and Multiple Comparisons (GRADE Guidance 45). Ann Intern Med. 2026 Mar 17; doi:10.7326/ANNALS-25-04273](https://ad-id.co/4dfO21B)
  - Summary: The GRADE evidence-to-decision (EtD) framework, originally designed for pairwise comparisons, has been updated to accommodate multiple comparisons, reflecting recent GRADE guidance. The new framework includes sections for question definition, assessment—including a “net effect” criterion—and conclusion, enabling structured decision-making across multiple interventions while considering outcomes, values, cost-effectiveness, equity, acceptability, and feasibility. Network meta-analyses can inform effect estimates and rankings, but the framework provides a transparent approach to integrate these with broader decision criteria. Limitations include that usability across diverse guideline contexts has not yet been widely tested.
- [Moreno R, Arias López MdP, Finazzi S. The customization of general outcome prediction models: a statistical exercise or a necessity? Crit Care Sci. 2026 Mar 16;38:e20260461. doi:10.62675/2965-2774.20260461](https://ad-id.co/4bRiPiU)
  - Summary: Since the introduction of the APACHE II score in 1985, predicting mortality in ICU patients has become routine, forming the basis for calculating the Standardized Mortality Ratio (SMR) and, later, the Standardized Resource Use (SRU) to assess ICU performance and cost-effectiveness. These metrics are widely used for benchmarking across registries such as ANZICS and ICNARC. However, the accuracy of SMR and SRU depends on the calibration of the underlying Outcome Prediction Models (OPMs), which can drift over time and across regions due to changes in management, case mix, admission/discharge policies, and end-of-life practices. Consequently, many registries update or customize OPM coefficients annually, though these changes are often unpublished.

**General Interest**

- [White NJ, Bagcchi S. Nicholas John White. Lancet Infect Dis. 2026 Apr;26(4):339. doi:10.1016/S1473-3099(26)00125-8](https://ad-id.co/4djjJqT)
  - Summary: Sir Nicholas John White, a pioneering physician and infectious disease researcher, passed away on February 1, 2026, at age 74. He was instrumental in developing and validating artemisinin-based combination therapies for malaria, leading to WHO recommendations for first-line treatment of uncomplicated and severe malaria. White held professorships at the University of Oxford and Mahidol University, directed the Mahidol Oxford Research Unit, and built a network of research sites across Asia and Africa. His contributions spanned malaria, dengue, tetanus, typhoid, and tuberculosis, influencing global health policy and saving millions of lives. Recognized internationally, he was a fellow of the Royal Society, Knight Commander of the Order of St Michael and St George, and recipient of the Canada Gairdner Global Health Award and Thailand's Prince Mahidol Award. White is survived by his wife, children, and grandchildren.
- [Fauntleroy K, Baron EJ, Humphries RM. In memoriam: Davise Honig Larone. J Clin Microbiol. 2026 Mar 18; doi:10.1128/jcm.01844-25](https://ad-id.co/3NPiSUv)
  - Summary: The editorial honors Davise Honig Larone, a highly influential clinical microbiologist who passed away on September 6, 2025, highlighting her lasting impact through her widely used textbook “Medically Important Fungi: A Guide to Identification” and her legacy as a mentor and leader in clinical mycology.

**Company News**

- [Insmed Incorporated. Insmed announces positive topline results from Phase 3b ENCORE study of ARIKAYCE® (amikacin liposome inhalation suspension) in patients with MAC lung disease. PR Newswire. March 23, 2026.](https://ad-id.co/3ObsZTC)
  - Summary: Insmed announced positive topline results from its Phase 3b ENCORE study evaluating ARIKAYCE® (amikacin liposome inhalation suspension) plus multidrug therapy in patients with a new occurrence of Mycobacterium avium complex (MAC) lung disease. The study met its primary endpoint—improvement in Respiratory Symptom Score at Month 13—and all multiplicity-controlled secondary endpoints, including culture conversion rates by Months 6, 12, 13, and durable conversion at Month 15. ARIKAYCE showed statistically significant improvements compared with placebo plus multidrug therapy, with a safety profile consistent with previous studies. Insmed plans to file a supplemental NDA with the FDA and submit data to Japan’s PMDA in the second half of 2026 to support potential label expansions. The ENCORE study included 425 patients across 177 global sites, assessing both efficacy and safety over a 12-month treatment period followed by 3 months for durability.
- [Iterum Therapeutics PLC. Iterum Therapeutics announces filing of winding up petition. GlobeNewswire. March 27, 2026.](https://ad-id.co/3QacS9n)
  - Summary: Iterum Therapeutics plc (in Provisional Liquidation) filed a petition in the High Court in Ireland on March 27, 2026, to wind up the company, appointing Damien Murran and Jennifer McMahon of Teneo Restructuring (Ireland) Limited as Joint Provisional Liquidators. The petition is scheduled to be heard on April 13, 2026, and if granted, the company and its subsidiaries will be wound up and ultimately dissolved. The decision follows limited cash resources, inability to raise capital, failure to meet Nasdaq listing requirements, and unsuccessful strategic alternatives, including commercialization challenges with ORLYNVAH™ (oral sulopenem) for uncomplicated urinary tract infections. The Joint Provisional Liquidators may manage withdrawal of ORLYNVAH™ from the U.S. market in consultation with the FDA and service providers. Iterum had focused on next-generation oral and IV antibiotics targeting multi-drug resistant pathogens.

**Target Trial Emulation**

- [Hernán MA, Dickerman BA, Swanson SA, Dahabreh IJ. Where do target trials come from? Specifying the protocol of a target trial when repurposing data for causal inference. Epidemiology. 2026 May;37(3):282-286 doi:10.1097/EDE.0000000000001951](https://ad-id.co/3ODOlt2)
  - Summary: This article explains that in real-world applications of the target trial framework, investigators must iteratively adapt their original causal question and hypothetical trial protocol based on the constraints of available observational data, making full prespecification impractical and highlighting the need for transparent reporting and predefined rules to guide and justify these data-driven adaptations.
- [Gupta AB, Walzl E, Ratz D, Horowitz JK, McLaughlin E, Pearlman T, et al. Outcomes associated with empiric azithromycin use among patients hospitalized with non-severe community-acquired pneumonia: emulation of a target trial. Clin Infect Dis. 2026 Apr 3; doi:10.1093/cid/ciag222](https://ad-id.co/3QDL0e0)
  - Summary: In a large target trial emulation of hospitalized adults with non-severe community-acquired pneumonia, adding empiric azithromycin to beta-lactam therapy did not improve time to clinical stability but was associated with reduced 30-day mortality and rehospitalization, with no significant differences in ICU transfer or antibiotic duration.
  - **Editorial Commentary:**

[Hartlage W, Nori P. Much ado about macrolides: revisiting empiric atypical coverage in non-severe community-acquired pneumonia. Clin Infect Dis. 2026 Apr 3; doi:10.1093/cid/ciag223](https://ad-id.co/4sxS9tI)

- [Doumat G, Ratz D, Horowitz JK, Gandhi TN, Petty LA, Malani AN, et al. Short versus longer antibiotic duration for community-acquired pneumonia: a multicenter target trial emulation. Ann Intern Med. 2026 Apr 14; doi:10.7326/ANNALS-25-03538](https://ad-id.co/426ZWUw)
  - Summary: In an observational target trial emulation of hospitalized non-ICU patients with community-acquired pneumonia who achieved clinical stability by day 3, 3–4-day antibiotic courses were used infrequently but showed similar 30-day mortality, readmission, urgent visits, and Clostridioides difficile infection rates compared to longer (≥5-day) treatment durations, suggesting comparable safety and effectiveness among eligible patients.
  - **Editorial Commentary:**

[Metlay JP, Choudhry NK. Antibiotics for pneumonia: from evidence to guidelines to action. Ann Intern Med. 2026 Apr 14; doi:10.7326/ANNALS-26-00795](https://ad-id.co/4sR0ZTP)

**Antibiotics - In vitro susceptibility**

- [Estabrook M, Streit C, Townsend A, Stone G, Kamat S, Sahm D, et al. The in vitro activity of aztreonam-avibactam and cefiderocol against globally collected clinical metallo-β-lactamase- and/or serine-carbapenemase-positive Enterobacterales isolates and the utility of commonly used in vitro diagnostic kits. Microbiol Spectr. 2026 Mar 31; doi:10.1128/spectrum.03820-25](https://ad-id.co/4sw7I54)
  - Summary: In a study of 269 clinical Enterobacterales isolates from global surveillance, the NG-test CARBA 5 and Xpert Carba-R in vitro diagnostic kits accurately detected major carbapenemases including NDM, KPC, and OXA-48-like enzymes, with complementary performance in detecting IMP and VIM variants, while antimicrobial susceptibility testing showed that aztreonam-avibactam demonstrated consistently high in vitro activity against carbapenemase-producing isolates, including metallo-β-lactamase producers, whereas cefiderocol showed more variable activity depending on carbapenemase type, highlighting both the diagnostic utility of rapid carbapenemase detection and the therapeutic potential of newer agents for multidrug-resistant Enterobacterales infections.

**Beta-Lactamases and Other Resistance Mechanisms**

- [Dorazio AJ, Kline EG, Squires KM, Shah S, Van Tyne D, Wu JY, et al. Ceftazidime-avibactam resistance evolution in Pseudomonas aeruginosa and implications for cross-resistance to other novel β-lactams. Antimicrob Agents Chemother. 2026 Apr 6; doi:10.1128/aac.01910-25](https://ad-id.co/4sw7CdK)
  - Summary: In paired baseline and post-ceftazidime-avibactam Pseudomonas aeruginosa isolates from treated patients, treatment-emergent resistance was driven by mutations in ampC and OXA β-lactamases and regulatory changes leading to AmpC overproduction and increased MexAB-OprM efflux activity, with frequent cross-resistance observed to ceftolozane-tazobactam, less frequent cross-resistance to imipenem-relebactam and cefepime-zidebactam, and lowest cross-resistance to cefiderocol, underscoring how resistance evolution during therapy can constrain subsequent β-lactam treatment options.
- [Loo E, Tellapragada C, Razavi M, Giske CG. Molecular epidemiology and genetic determinants of β-lactam resistance in Pseudomonas aeruginosa isolates from cystic fibrosis patients in Stockholm, Sweden. Clin Microbiol Infect. 2026 Apr 7; doi:10.1016/j.cmi.2026.03.034](https://ad-id.co/4sIcLQk)
  - Summary: In a whole-genome sequencing study of Pseudomonas aeruginosa isolates from cystic fibrosis patients in Sweden, investigators identified a high burden of multidrug- and extensively drug-resistant strains, including the epidemic AUST-03 (ST242) clone, with β-lactam resistance primarily driven by chromosomal mutations such as OprD porin loss leading to carbapenem resistance, alterations in AmpC regulation contributing to cephalosporin resistance, and additional mutations in penicillin-binding proteins and iron metabolism genes potentially associated with cefiderocol resistance, highlighting the genomic drivers and epidemiology of antimicrobial resistance in chronic CF-associated infections.
- [Lu TY, Wu SJ, Chu YF, Ni XB, Lv LC, Sun J, et al. Outer membrane vesicles transmit blaNDM-5 and package metallo-β-lactamases to promote antibiotic resistance in Escherichia coli. J Antimicrob Chemother. 2026 Mar 31;81(4):dkag123 doi:10.1093/jac/dkag123](https://ad-id.co/3OFgmjU)
  - Summary: This study demonstrates that outer membrane vesicles from Escherichia coli carrying the blaNDM-5 gene can mediate carbapenem resistance through dual mechanisms, including horizontal transfer of plasmid-encoded resistance genes and extracellular delivery of active NDM-5 carbapenemase that degrades antibiotics and protects nearby bacteria, with proteomic changes in vesicles indicating broader bacterial adaptation to carbapenem exposure.
- [Royer G, Danjean M, Rodriguez C, Fihman V, Gallois E, Tessier E, et al. Piperacillin-tazobactam resistance in Klebsiella pneumoniae is often associated with IS26-mediated blaSHV-1 amplification in a widespread Klebsiella-adapted plasmid. Antimicrob Agents Chemother. 2026 Mar 24; doi:10.1128/aac.01682-25](https://ad-id.co/4sxLPmc)
  - Summary: In an analysis of Klebsiella pneumoniae isolates resistant to piperacillin-tazobactam but susceptible to cephalosporins, resistance was driven primarily by diverse genetic mechanisms including blaOXA-1 presence, promoter mutations leading to chromosomal blaSHV overexpression, and especially plasmid-mediated amplification of blaSHV-1—often carried on a conserved IncFIB(K)_1_Kpn3 plasmid with an IS26-associated transposon—where increased gene copy number correlated with higher antibiotic MICs, suggesting the emergence of an epidemic plasmid-driven mechanism of resistance that may be missed by standard diagnostic approaches.
- [Lu R, Wu Y, Ma R, Feng R, Qin B, Zhou X, et al. Ceftazidime/avibactam-resistant carbapenem-resistant Klebsiella pneumoniae in China: a nationwide multicenter study. Int J Antimicrob Agents. 2026 Jun;67(6):107774 doi:10.1016/j.ijantimicag.2026.107774](https://ad-id.co/3ODUAgr)
  - Summary: In a large multicentre surveillance study of carbapenem-resistant Klebsiella pneumoniae across China, ceftazidime-avibactam resistance was uncommon (2.1%) but widely distributed and mainly associated with epidemic ST11 lineages carrying extensive multidrug resistance, with most resistant isolates driven by NDM production or NDM+KPC co-producers, while a small subset of KPC-2–only resistant strains showed increased blaKPC-2 expression and gene copy number alongside porin alterations and efflux pump involvement, collectively highlighting multiple genomic pathways underlying emerging ceftazidime-avibactam resistance.
- [Pérez-Rodríguez G, Aja-Macaya P, González-Pinto L, Taltavull B, Tarriño-León M, Gallardo-García MdM, et al. Emergence of KPC-producing Pseudomonas aeruginosa in Spain: insights into an outbreak and resistance to the novel carbapenem/β-lactamase inhibitor combinations imipenem/relebactam and meropenem/vaborbactam. Antimicrob Agents Chemother. 2026 Mar 27; doi:10.1128/aac.01657-25](https://ad-id.co/424jxVl)
  - Summary: This report describes a 2025 hospital outbreak of carbapenemase-producing Pseudomonas aeruginosa in Spain involving four patients and an environmental sink isolate, all belonging to ST253 and exhibiting a difficult-to-treat resistance phenotype with resistance to multiple carbapenem/β-lactamase inhibitor combinations; genomic and functional analyses identified plasmid-encoded blaKPC-2 together with OprD porin deficiency as key drivers of resistance, with evidence of synergistic effects between carbapenemase production and porin loss leading to high-level resistance even to agents typically active against KPC producers, such as imipenem/relebactam and meropenem/vaborbactam.

**Adverse Effects from Antimicrobial Agents**

- [Mitri EA, Fletcher LR, Vogrin S, Barnes S, Powell N, Peter J, et al. Direct oral challenge for penicillin allergy: the International Network of Antibiotic Allergy Nations (iNAAN) study. Clin Infect Dis. 2026 Apr 1; doi:10.1093/cid/ciag082](https://ad-id.co/4stmGZT)
  - Summary: In a large multicenter international hybrid effectiveness-implementation study across 40 hospitals, inpatient penicillin direct oral challenge using a digital allergy assessment toolkit safely delabeled most reported penicillin allergies with very low rates of serious adverse events, and in a target trial emulation analysis, was associated with substantially increased subsequent penicillin prescribing and reduced use of WHO Watch/Reserve antibiotics, demonstrating both clinical safety and improved antibiotic stewardship outcomes alongside successful implementation of a digital allergy tool.
- [Mitri EA, Vogrin S, Paynter C, Fletcher LR, Peter J, Powell N, et al. Direct oral challenge for penicillin allergy: a hospital implementation evaluation via the International Network of Antibiotic Allergy Nations (iNAAN) study. Clin Infect Dis. 2026 Apr 1; doi:10.1093/cid/ciag083](https://ad-id.co/4sBSZWD)
  - Summary: In an international multicenter prospective implementation study, penicillin direct oral challenge delivered through a digital toolkit was found to be acceptable, feasible, and sustainable across diverse hospital settings, with audit-and-feedback strategies increasing uptake—particularly among pharmacists—and demonstrating maintained safety across heterogeneous protocols, supporting a pragmatic and scalable model that allows local adaptation of penicillin allergy delabeling practices.
- [Zhao S, Mu G, Liu X, Ma L, Yang L, Zhou Y. Pathophysiology, risk factors and clinical management for polymyxin-associated acute kidney injury. J Antimicrob Chemother. 2026 Apr 9;81(5):dkag132 doi:10.1093/jac/dkag132](https://ad-id.co/3QBj3n8)
  - Summary: This review discusses polymyxin-associated acute kidney injury (PA-AKI), a major limitation in the use of last-line polymyxins for multidrug-resistant Gram-negative infections, outlining its pathophysiology, risk factors, and clinical management strategies, and emphasizing the importance of minimizing concurrent nephrotoxic exposures, implementing standardized monitoring of renal function and drug levels, and exploring emerging biomarkers, while highlighting gaps in clinical validation and the need for real-world evidence, genetic risk stratification, and development of less nephrotoxic polymyxin analogues.
- [Maharaj A, Omar M. Minocycline-induced hyperpigmentation. N Engl J Med. 2026 Apr 1;394:e24 doi:10.1056/NEJMicm2513782](https://ad-id.co/3QCSkXj)
  - Summary: This case report describes a 68-year-old woman with rosacea who developed dark hyperpigmented patches on her arms and legs six weeks after initiating daily minocycline therapy, with skin changes emerging approximately two weeks after starting the antibiotic, suggesting drug-induced pigmentation as a potential adverse effect of long-term minocycline use.
- [Petersiel N, Legg A, Ong SWX, Ovadia A, Meagher N, Price DJ, et al. Association between β-lactam exposure and clearance of bacteremia and acute kidney injury in patients with methicillin-resistant Staphylococcus aureus bloodstream infections – a post hoc analysis of the CAMERA2 trial. Clin Microbiol Infect. 2026 Apr 12; doi:10.1016/j.cmi.2026.04.006](https://ad-id.co/4stt96P)
  - Summary: In a post-hoc analysis of the CAMERA2 trial evaluating adjunctive β-lactam use in MRSA bacteremia, β-lactam exposure showed a duration-dependent association with outcomes, where any β-lactam use (including empiric short exposure) was linked to faster bacterial clearance, but longer or prolonged β-lactam therapy was increasingly associated with higher risk of acute kidney injury, suggesting that short empiric β-lactam courses may improve microbiological outcomes without substantially increasing nephrotoxicity.

**Antibiotic Stewardship and Hospital in the Home**

- [Ilges D, Jensen K, Draper E, Thompson E, Quillen J, Arensman Hannan K, et al. Trends in long duration antibiotic prescribing following focused outpatient antimicrobial stewardship efforts across a large healthcare enterprise. Clin Infect Dis. 2026 Apr 9; doi:10.1093/cid/ciag244](https://ad-id.co/4szq0Td)
  - Summary: In a large multicenter retrospective cohort study of over 640,000 ambulatory encounters, annual syndrome-specific outpatient antibiotic stewardship interventions were associated with significant reductions in long-duration antibiotic prescribing, particularly in ASP-targeted primary and urgent care departments compared with non-targeted settings, with improvements observed across both priority and non-priority diagnoses, supporting long-duration antibiotic prescribing as a useful metric for evaluating outpatient stewardship effectiveness.

**Infection Prevention / Antibiotic Prophylaxis**

- [Ross I, Bath D, Wells J, Dreibelbis R, Ejemot-Nwadiaro R, Esteves Mills J, et al. Cost-effectiveness and benefit-cost analyses of promoting handwashing with soap: a systematic review. PLoS Med. 2026 Apr 3; doi:10.1371/journal.pmed.1004982](https://ad-id.co/3OGCbzD)
  - Summary: This systematic review of economic evaluations on handwashing promotion interventions in non-healthcare settings found limited but generally supportive evidence that promoting handwashing with soap is likely cost-effective, with available high- and medium-quality studies estimating favorable cost per disability-adjusted life-year averted and positive benefit–cost ratios, although the evidence base is constrained by a small number of empirical studies, heavy reliance on modelling, and limited consideration of sustained adoption and respiratory infection outcomes.

**Bloodstream Infections and Endocarditis**

- [Jeffs MA, Li N, Ogunkoya O, Duncan DB, Hall CW, Nemer A, et al. Cefazolin inoculum effect and cefazolin microbiological treatment failure in serious methicillin-susceptible Staphylococcus aureus infections: a multi-center retrospective cohort study. J Infect Dis. 2026 Apr 2; doi:10.1093/infdis/jiag199](https://ad-id.co/4sAbjPU)
  - Summary: In a multicenter retrospective cohort of patients with serious methicillin-susceptible Staphylococcus aureus infections treated with cefazolin, the presence of a cefazolin inoculum effect (CzIE) was not associated with increased 90-day all-cause mortality but was significantly associated with higher rates of microbiological treatment failure, suggesting that CzIE may have important implications for therapeutic efficacy despite similar survival outcomes and may be useful for guiding antibiotic selection in severe MSSA infections.
- [Olivares-Navarro P, Pérez-Rodríguez MT, Sousa A, Goikoetxea-Agirre AJ, Blanco Vidal MJ, Plata A, et al. Mortality risk of ESBL producers in Escherichia coli bacteraemia: a comprehensive analysis using the PROBAC cohort. J Antimicrob Chemother. 2026 Apr 8;81(5):dkag130 doi:10.1093/jac/dkag130](https://ad-id.co/4sxS9Ke)
  - Summary: In a large prospective multicentre cohort study of Escherichia coli bloodstream infections across 26 Spanish hospitals, ESBL-producing isolates were associated with higher crude 30-day mortality compared to non-ESBL strains; however, this association was no longer statistically significant after adjustment for baseline confounders and differences in appropriate empirical antibiotic therapy using propensity score methods, suggesting that the increased mortality observed in unadjusted analyses is largely explained by treatment and patient-related factors rather than ESBL production itself.

**Gastrointestinal Tract Infections**

- [Wei Q, Lu G, Diao H, Jiang S, Zhang Y, Zhang Y, Shi T, Li Y. The incidence and risk factors of antibiotic-associated diarrhea in critically ill patients: a systematic review and meta-analysis. J Gastroenterol Hepatol. 2026 Mar 10; doi:10.1111/jgh.70315](https://ad-id.co/3ODOlJy)
  - Summary: This meta-analysis of critically ill patients found that antibiotic-associated diarrhea (AAD) and Clostridioides difficile–associated diarrhea (CDAD) are common complications in the ICU, with pooled incidences of 29% and 12% respectively, and identified multiple clinical, therapeutic, and severity-related risk factors—including broad-spectrum antibiotic use (notably cephalosporins, β-lactam/β-lactamase inhibitor combinations, glycopeptides, carbapenems), proton pump inhibitor exposure, longer antibiotic duration, higher illness severity, and longer ICU stay—highlighting high-risk patient profiles that may guide prevention strategies in critical care settings.
- [Beebe MA, Sorg JA. An update on Clostridioides difficile population structure and genomics. Clin Microbiol Rev. 2026 Mar 31; doi:10.1128/cmr.00296-25](https://ad-id.co/425fRTn)
  - Summary: This review of Clostridioides difficile phylogeny describes how the species is divided into five main clades and additional cryptic clades with distinct genetic and clinical characteristics, noting that clades 2 and 5 are most strongly associated with severe disease and transmission, while clades 3 and 4 possess unique genomic features and pathogenicity loci; it highlights that much of the diversity and evolution in virulence and antibiotic resistance arises from variation in the accessory genome mediated by mobile genetic elements such as prophages, transposons, and plasmids, underscoring the importance of including diverse ribotypes and clades in both phylogenetic and phenotypic studies.

**Bone and Joint Infections**

- [Hanssen JLJ, Pijls B, Wouthuyzen-Bakker M, Manning L, Campbell D, van Prehn J, et al. Practice variation, outcomes and definitions of suppressive antimicrobial therapy for prosthetic joint infections: a systematic review and expert consensus statement. Clin Infect Dis. 2026 Apr 13; doi:10.1093/cid/ciag251](https://ad-id.co/3QDL0uw)
  - Summary: This systematic review of suppressive antimicrobial therapy (SAT) for periprosthetic joint infection found substantial heterogeneity in how SAT is defined and applied across studies, with notable differences between U.S. and European practice patterns—where SAT is more commonly used after debridement, antibiotics, and implant retention (DAIR) in the U.S. and more often in non-curative strategies in Europe—and reported pooled success rates of approximately 70–74%, ultimately leading to a Delphi-based consensus effort to standardize definitions distinguishing SAT from extended antimicrobial therapy to improve consistency in research and clinical practice.

**Skin and Soft Tissue Infections**

- [Mitchell KB, Valente SA, Snider HC, Fowler AM, Allison KH, Pass HA, et al. American Society of Breast Surgeons, Society of Breast Imaging, and College of American Pathology 2025 guidelines for the management of infectious and inflammatory lesions of the breast. JAMA Surg. 2026 Apr 1; doi:10.1001/jamasurg.2026.0613](https://ad-id.co/4stbUCH)
  - Summary: This expert consensus guideline addresses the management of common infectious and inflammatory breast conditions, emphasizing the importance of distinguishing infectious from noninfectious lactational mastitis to guide appropriate intervention, recommending procedural drainage and antibiotics for infectious mastitis with collections, advocating pathology-confirmed diagnosis and stepwise, often immunomodulatory treatment strategies for granulomatous mastitis including intralesional or systemic steroids and biologics in refractory cases, and outlining tailored antibiotic, drainage, or surgical approaches for periductal mastitis with squamous metaplasia, thereby standardizing care for conditions historically managed with wide variability.

**Sexually Transmitted Infections**

- [Kimble AD, Manabe YC, Melendez JH. Evidence-based assessment of the role of pharyngeal gonorrhea and commensal Neisseria species in the emergence of antimicrobial resistance in Neisseria gonorrhoeae: data gaps and future research. J Infect Dis. 2026 Apr 3; doi:10.1093/infdis/jiag197](https://ad-id.co/4sCJ8zN)
  - Summary: This review highlights the role of pharyngeal Neisseria gonorrhoeae infections as an important but often asymptomatic reservoir for ongoing transmission and a key driver of antimicrobial resistance, emphasizing that horizontal gene transfer between NG and commensal Neisseria species in the oropharynx contributes significantly to the acquisition of resistance determinants, and underscoring the need for improved understanding of pharyngeal pathogenesis and genetic exchange mechanisms to address the accelerating global threat of gonococcal antimicrobial resistance.

**CNS Infections**

- [Duerlund LS, Larsen L, Storgaard M, Mens H, Wiese L, Jepsen MPG, et al. Infectious encephalitis among adults: a prospective and population-based cohort study. Clin Microbiol Infect. 2026 Apr 10; doi:10.1016/j.cmi.2026.04.002](https://ad-id.co/4sDmmaV)
  - Summary: In a nationwide Danish prospective cohort study of adults hospitalized with infectious encephalitis from 2015–2023, the incidence was low at 1.18 per 100,000 per year, with varicella zoster virus and herpes simplex virus type 1 as the most common etiologies, and clinical presentation frequently including nonspecific symptoms such as confusion, headache, and personality changes; outcomes remained poor with 22% requiring ICU admission and 15% six-month mortality, which was particularly increased in patients with immunocompromise, HSV-1 infection, and low Glasgow Coma Scale scores at admission.

**Respiratory Tract Infections**

- [Ramos B, Khatri Vadlamudi N, Golden AR, Martin I, Tyrrell G, Brousseau N, et al. Invasive pneumococcal disease epidemiology and conjugate vaccines in Canada, 2000-2019. JAMA Netw Open. 2026 Apr 9;9(4):e266005 doi:10.1001/jamanetworkopen.2026.6005](https://ad-id.co/4sBET7j)
  - Summary: In a large Canadian cross-sectional study of over 37,000 invasive pneumococcal disease isolates from 2000–2019, introduction of PCV7 and PCV13 was associated with substantial reductions in vaccine-type disease in children and indirect reductions in adults, although some serotypes persisted or re-emerged and non-vaccine serotypes increasingly contributed to disease burden; modelling suggested that newer vaccines such as PCV15, PCV20, and PCV21 could expand serotype coverage by approximately 23%–49%, but ongoing shifts toward nonvaccine serotypes highlight the need for broader-spectrum pneumococcal vaccines and improved adult immunization strategies.
- [Drozdinsky G, Ness A, Sabbah M, Kushnir S, Bishara J, Eliakim-Raz N. Doxycycline in the treatment of outpatient mild community-acquired pneumonia: a propensity matched retrospective cohort study. Clin Microbiol Infect. 2026 Mar 28; doi:10.1016/j.cmi.2026.03.031](https://ad-id.co/3QDCCLB)
  - Summary: In a large propensity-matched retrospective cohort study of over 100,000 adults with outpatient community-acquired pneumonia in Israel, empiric doxycycline monotherapy was associated with a lower risk of early antibiotic regimen switching and slightly reduced 30-day hospitalization compared with other commonly used antibiotics such as macrolides, cephalosporins, and amoxicillin/clavulanate, while showing no significant difference in 30-day mortality, supporting doxycycline as a potentially non-inferior empiric option for mild outpatient CAP.

**Urinary Tract Infections**

- [Luck ME, Martin A, Punja S, Kamar J, Zuchinali P, Edgecomb AG, et al. Definitions and rates of treatment failure in females with uncomplicated urinary tract infection: a systematic literature review. J Antimicrob Chemother. 2026 Apr 8;81(5):dkag112 doi:10.1093/jac/dkag112](https://ad-id.co/4sw7IlA)
  - Summary: This systematic review of studies on uncomplicated urinary tract infections in women found substantial heterogeneity in how treatment failure is defined—ranging from microbiological and clinical outcomes to antibiotic re-prescription and composite endpoints—leading to widely variable reported failure rates across studies; however, more consistent estimates emerged when using composite or healthcare-utilization–based definitions, highlighting the need for standardized, clinically meaningful definitions and better-defined outcome assessment timepoints in future UTI research.
- [Wu T, Gupta K, McCreary EK, Turbett SE, Lazarus JE. Oral fosfomycin tromethamine for UTI: guidance for clinicians. CMI Commun. 2026 Apr 3; doi:10.1016/j.cmicom.2026.105190](https://ad-id.co/41AEs2f)
  - Summary: This narrative review evaluates the role of oral fosfomycin in the treatment of urinary tract infections and concludes that while it remains a useful but rarely preferred option, its use should generally be reserved for specific clinical situations—such as uncomplicated cystitis in men and women, certain catheter-associated or pregnancy-related infections, chronic prostatitis, and UTI prophylaxis—primarily when caused by Escherichia coli or Enterococcus faecalis and when better-established alternatives are unsuitable, while cautioning against its use for pyelonephritis, febrile UTI, or infections due to non–E. coli gram-negative organisms due to limited efficacy and inconsistent susceptibility data.

**Infections in Children**

- [Wen SCH, Hardy MJ, Aslan AT, Harris PNA, Chatfield MD, Lau CL, et al. Empirical antibiotic activity and outcomes in pediatric gram-negative bloodstream infections: a rank-based composite outcome analysis. Clin Infect Dis. 2026 Apr 9; doi:10.1093/cid/ciag234](https://ad-id.co/4sw7Cug)
  - Summary: In a multicenter prospective Australian pediatric cohort of gram-negative bloodstream infections, receipt of active empirical antibiotic therapy (AET) compared with inactive empirical therapy (IET) was not associated with improved outcomes when assessed using a ranked composite endpoint incorporating 30-day mortality, time to death, ICU admission, relapse, and hospital length of stay, with adjusted analyses showing no significant difference between groups, suggesting that in high-resource pediatric settings the clinical impact of initial empirical antibiotic activity may be less pronounced than traditionally expected.
- [Xu Y, Akinbi H, Shen Z, Zhu J, Shi L, Du L, et al. Clinical care practices shape microbiome-associated bloodstream infection risk in geographically distinct NICUs. Clin Infect Dis. 2026 Mar 28; doi:10.1093/cid/ciag213](https://ad-id.co/4szSuMF)
  - Summary: In a prospective cohort study of 127 preterm infants across NICUs in the United States and China, distinct gut and skin microbiome profiles were observed that closely mirrored local bloodstream infection epidemiology, with Staphylococcus aureus predominating in the US site and Klebsiella pneumoniae and Enterococcus species in the Chinese site; analysis showed that modifiable clinical practices such as antibiotic exposure and intravenous catheter use had a stronger influence on microbiome composition than geographic location, and implicated the skin microbiome as an underrecognized reservoir contributing to neonatal bloodstream infections.

**Mycobacterial Infections**

- [Shin E, Dousa KM, Nantongo M, Kurz SG, Holland SM, Kreiswirth BN, et al. Synergistic activity of dual β-lactams against Mycobacterium avium complex. Microbiol Spectr. 2026 Mar 31; doi:10.1128/spectrum.04001-25](https://ad-id.co/4szq19J)
  - Summary: This study systematically evaluates the in vitro activity of 16 β-lactam antibiotics and their combinations against Mycobacterium avium complex (MAC), demonstrating that while single β-lactams show limited efficacy—particularly against M. avium—specific combinations, especially those pairing penems or carbapenems with penicillins or cephalosporins, exhibit potent synergistic and bactericidal activity against M. intracellulare and, to a lesser extent, M. avium at clinically achievable pharmacokinetic/pharmacodynamic exposures, thereby identifying repurposed β-lactam combination strategies as a promising and potentially rapidly translatable approach for treating refractory MAC pulmonary disease.
- [Cheng W, Du C, Zhang S, Jiang G, Hu W. Advances in therapeutic strategies against Mycobacterium marinum infection. Expert Opin Ther Targets. 2026 Apr 1; doi:10.1080/14787210.2026.2652893](https://ad-id.co/4szSvjH)
  - Summary: This systematic review summarizes current and emerging treatment strategies for Mycobacterium marinum infection, highlighting the lack of a standardized therapeutic protocol and reliance on oral antibiotic regimens such as macrolides, tetracyclines, quinolones, and rifampicin–ethambutol combination therapy, which achieves high cure rates especially in deep or refractory disease; it also discusses adjunctive and novel approaches including photodynamic therapy, local hyperthermia, surgical debridement, and immunomodulatory support, emphasizing their potential to improve outcomes, shorten treatment duration, and overcome antimicrobial resistance, while underscoring the need for multicenter trials to establish standardized, evidence-based, multimodal treatment frameworks.
- [Ni R, Liu Y, Armanni A, Ghisleni G, Fumagalli S, An Y, et al. From T-cell sensitization to molecular–intelligent stratification: a roadmap for precision diagnosis of latent tuberculosis infection. Clin Microbiol Rev. 2026 Mar 30; doi:10.1128/cmr.00258-25](https://ad-id.co/4sBtzrY)
  - Summary: This review proposes a shift in latent tuberculosis infection (LTBI) management from traditional tuberculin skin testing and interferon-gamma release assays toward an integrated molecular–intelligent framework that combines host and pathogen biomarkers, multi-analyte diagnostic indexing, and AI-enabled multi-omics and point-of-care technologies, alongside a CD4- and age-stratified clinical decision approach for key populations such as people living with HIV, children, immunosuppressed individuals, and pregnant women; it emphasizes a three-tier diagnostic strategy (triage, confirmation, and treatment monitoring) and highlights the need for standardized thresholds, cross-population validation, and resource-adapted implementation to enable scalable, precision-based LTBI screening and accelerate tuberculosis elimination efforts.
- [Zhan S, Liu W, Yang L, Chen Z, He T, Deng G, et al. Fixed-dose rifapentine–isoniazid (1HP) for tuberculosis preventive treatment in Chinese adults: a prospective real-world safety and pharmacokinetic study. Clin Infect Dis. 2026 Apr 13; doi:10.1093/cid/ciag247](https://ad-id.co/4sviokE)
  - Summary: In a prospective study of 136 non-HIV adults in China receiving fixed-dose one-month daily rifapentine plus isoniazid (1HP) for tuberculosis preventive treatment, the regimen demonstrated good tolerability with a 76.5% treatment completion rate and low incidence of severe adverse events, while pharmacokinetic analysis showed that most participants achieved adequate rifapentine exposure despite modest weight-related variability, supporting the feasibility and effectiveness of fixed-dose 1HP for routine programmatic implementation in this population.
- [Death of stray cat reveals hidden spread of a tuberculosis bacterium. Nature. 2026 Apr 9; doi:10.1038/d44151-026-00063-3](https://ad-id.co/41sr4gF)
  - Summary: The first reported case of Mycobacterium orygis infection in a domestic stray kitten in Mumbai, presenting as severe pulmonary tuberculosis confirmed through PCR and genetic sequencing, suggests possible underrecognized circulation of this tuberculosis-related bacterium across humans, animals, and urban environments, with implications that stray animals may serve as sentinel indicators of broader zoonotic transmission and underscoring the need for integrated One Health surveillance in densely populated cities.
- [Edoo Z, Grosse C, Maitre T, Frita R, Chauffour A, Fournier Le Ray L, et al. Alpibectir–ethionamide combination (AlpE) for the treatment of tuberculosis. Nat Commun. 2026 Apr 7. doi:10.1038/s41467-026-71460-6](https://ad-id.co/41wZf6U)
  - Summary: A preclinical and early clinical study of the transcriptional regulator–targeting compound alpibectir (BVL-GSK098) demonstrates that it enhances Mycobacterium tuberculosis susceptibility to the second-line tuberculosis drugs ethionamide and prothionamide by upregulating MymA expression via VirS binding, resulting in rapid bactericidal activity in vitro and in mouse models, reduced emergence of ethionamide resistance, and maintained efficacy against drug-resistant strains including inhA promoter mutants, while showing safety in a Phase 1 trial and suggesting potential for dose reduction of ethionamide/prothionamide to improve tolerability and adherence in tuberculosis treatment.
- [Motaung B, Holtzhausen A-R, Stanley K, van Rensburg I, Snyders CI, Loxton AG. The value of the monocyte-to-lymphocyte ratio and osteopontin (SPP1) in tuberculosis treatment response monitoring. Sci Rep. 2026 Apr 1; doi:10.1038/s41598-026-46176-8](https://ad-id.co/4tJNu99)
  - Summary: In a prospective observational study comparing healthy controls (n=32 plasma, n=9 BAL) and newly diagnosed tuberculosis patients (n=82 plasma, n=28 BAL) with serial sampling through treatment up to 6 months, investigators found that tuberculosis was associated at diagnosis with elevated monocyte-to-lymphocyte ratio and increased inflammatory mediators including plasma osteopontin (OPN), IL-6, VEGF-A, and sFasL, all of which generally declined during treatment by month 6, while OPN showed dynamic changes over time with early reduction followed by later increase, suggesting that systemic immune and protein signatures such as MLR and OPN may have potential utility as biomarkers for early TB diagnosis and treatment monitoring despite the exploratory nature of the findings.

**Fungal Infections and Antifungal Agents**

- [Gold JAW, Benedict K, Lyman M, Toda M, Little JS, Ostrosky-Zeichner L. *Candida glabrata* emerges as the most common cause of candidemia: analysis of a large hospital-based database, United States, 2016–2024. Clin Infect Dis. 2026 Apr 13; doi:10.1093/cid/ciag252](https://ad-id.co/423xKlt)
  - Summary: In a large U.S. hospital database analysis of 13,177 candidemia-associated hospitalizations from 2016 to 2024, species distribution shifted over time with Candida glabrata overtaking Candida albicans as the most common pathogen and Candida auris emerging as a significant contributor, rising to the fifth most frequently identified species by 2024, highlighting evolving epidemiology and the need for continued surveillance and strict adherence to diagnostic and treatment guidelines for candidemia.
- [Vuong NN, McClung DR, Aitken SL, Rausch CR. New antifungals for the treatment of invasive mold disease. Clin Microbiol Infect. 2026 Apr 6; doi:10.1016/j.cmi.2026.03.035](https://ad-id.co/41BtwBs)
  - Summary: This review summarizes recent advances in antifungal drug development targeting invasive mold infections listed by the WHO fungal priority pathogens list, highlighting newly approved agents such as rezafungin, ibrexafungerp, and oteseconazole alongside investigational therapies including fosmanogepix, olorofim, and other pipeline compounds; it emphasizes emerging clinical data suggesting improved efficacy and safety profiles for several of these agents against difficult-to-treat molds such as Aspergillus, Fusarium, and Mucorales, while noting ongoing challenges in antifungal development due to limited drug targets, resistance emergence, and toxicity concerns, and underscoring the potential of these new therapies to expand treatment options for invasive fungal disease.
- [Staiger MFP, Souza GHA, Venturini J, Peres NTA, Lucini F, Santos DA, et al. The impact of toll-like receptor polymorphisms on susceptibility to fungal infections: a systematic review of genetic and clinical evidence. Clin Microbiol Infect. 2026 Apr 8; doi:10.1016/j.cmi.2026.03.042](https://ad-id.co/4sxSa0K)
  - Summary: This systematic review and meta-analysis of 1,611 patients across ten countries identified 70 toll-like receptor (TLR) gene variants associated with invasive fungal infections, with polymorphisms in TLR2, TLR4, and TLR9 being most frequently reported in cases of invasive aspergillosis and candidiasis, particularly among immunocompromised populations such as transplant recipients and patients with malignancies; pooled analysis suggested that TLR polymorphisms were associated with an approximately twofold increased risk of fungal infection, although the overall evidence base remains largely descriptive and highlights the need for more robust comparative studies with appropriate control populations.
- [Pasqualotto AC, Le T, Vieceli T, Brown L, Oladele R, Bahr NC. Histoplasmosis: a missing piece in the global efforts to end HIV deaths. Lancet Infect Dis. 2026 Mar 12; doi:10.1016/S2352-3018(26)00006-8](https://ad-id.co/425fS9T)
  - Summary: Histoplasmosis is a major but neglected opportunistic infection contributing significantly to mortality in people with advanced HIV disease, now recognised as endemic across multiple regions including sub-Saharan Africa and Southeast Asia, yet its global burden remains underdiagnosed due to extremely limited access to highly sensitive Histoplasma antigen tests; despite effective but toxic or suboptimal antifungal treatments such as amphotericin B and itraconazole, progress is hindered by diagnostic and treatment gaps, highlighting the need for urgent investment in research, expanded diagnostic access, and integration of antigen screening into advanced HIV care, with Latin America’s coordinated surveillance and treatment model offering a potential framework for global scale-up.
- [Bartalucci C, Russo C, Raiola AM, Gambella M, Di Pilato V, Morici P, et al. Targeted screening to predict Magnusiomyces infections in hematopoietic cell transplant recipients: evidence from an outbreak setting. J Fungi. 2026 Apr 1;12(4):254 doi:10.3390/jof12040254](https://ad-id.co/3QDL0L2)
  - Summary: In a hematopoietic cell transplantation (HCT) unit outbreak of Magnusiomyces clavatus, targeted pharyngeal and rectal swab screening combined with prolonged culture and whole-genome sequencing identified five colonizations and five invasive breakthrough infections with high mortality (80% despite antifungal therapy), confirmed clonal transmission among isolates without an identified environmental source, and showed that an enhanced two-phase screening strategy provided minimal additional yield beyond routine surveillance; a broader retrospective review further highlighted that Magnusiomyces spp. infections are rare, occur infrequently in hematological malignancies, and remain difficult to detect early, underscoring ongoing challenges in surveillance, prevention, and outbreak control in high-risk HCT settings.
- [Choi JY, Chiang A, Ben Mamoun C. Coenzyme A metabolism in fungi: a new frontier in antifungal therapy. Clin Microbiol Rev. 2026 Mar 31; doi:10.1128/cmr.00307-25](https://ad-id.co/3QBTVwA)
  - Summary: Fungal infections cause a substantial global health burden with approximately 150 million severe cases and 3.8 million deaths annually, a situation worsened by a limited antifungal drug pipeline and increasing multidrug resistance, prompting renewed interest in the fungal coenzyme A (CoA) biosynthesis pathway as a novel and selective therapeutic target; recent research highlights compounds that inhibit this essential fungal metabolic pathway and potentially act as both standalone antifungals and adjuvants that enhance susceptibility to existing drugs and overcome resistance mechanisms, positioning CoA metabolism as a promising strategy for next-generation antifungal development and improved treatment outcomes.
- [Murphy SG, Ross T, Fitzgerald A, Gauthier NPG, Keller E, Barker E, et al. Detecting healthcare-associated transmission and antifungal resistance in *Candida auris* via whole genome sequencing. J Clin Microbiol. 2026 Mar 25; doi:10.1128/jcm.01348-25](https://ad-id.co/4stbUTd)
  - Summary: This multicenter genomic epidemiology study of 68 Candida auris isolates from 31 hospitalized patients used whole genome sequencing and SNP analysis to define thresholds for strain relatedness in healthcare-associated outbreaks, showing a maximum intra-patient variation of 14 SNPs and identifying five probable transmission clusters with a median of 5 SNP differences (range 0–12) strongly supported by epidemiologic links; it further demonstrated that cases detected more than one month after admission were highly predictive of nosocomial transmission clusters, and highlighted the value of WGS in simultaneously tracking transmission dynamics and identifying key antifungal resistance mutations (including ERG11, MRR1, FKS1, and FUR1 variants), underscoring its importance for outbreak surveillance and resistance prediction in multidrug-resistant C. auris infections.

**Virulence**

- [Le Bris J, Varet H, Rocha EPC, Rendueles O. Plug-and-play evolution of the *Klebsiella pneumoniae* capsule locus enables serotype exchange across genetic backgrounds. PLoS Biol. 2026 Mar 25; doi:10.1371/journal.pbio.3003724](https://ad-id.co/426ZXry)
  - Summary: This experimental and evolutionary study of the polysaccharide capsule locus in Klebsiella pneumoniae demonstrates that capsule swaps across diverse genetic backgrounds have minimal impact on global gene expression and only marginal fitness costs, indicating that capsule loci function as modular “plug-and-play” genetic units; using engineered capsule exchanges combined with transcriptomics, fitness assays, and evolution experiments, the study shows that adaptation to capsule-costly environments leads to consistent reductions in capsule production across all capsule (K) types, while key virulence-associated traits such as biofilm formation and hypermucoviscosity remain conserved, highlighting how capsule exchangeability shapes host–pathogen interactions and drives the evolutionary success and ecological versatility of K. pneumoniae.

**Host Factors in Infection**

- [Xue Y, Karim AM, Sia WR, Han F, Chan KL, Chua NG, et al. Restoration of mucosa-associated invariant T-cell function in healthcare-associated bacterial infections supports recovery of carbapenem efficacy against resistant bacteria ex vivo. J Infect Dis. 2026 Apr 2; doi:10.1093/infdis/jiag151](https://ad-id.co/4sR10qR)
  - Summary: This study investigates mucosa-associated invariant T (MAIT) cells as a host-directed therapeutic strategy against antimicrobial resistance in healthcare-associated infections, demonstrating that cognate antigen combined with cytokines such as IL-15 or IL-2 plus IL-7 enhances MAIT cell cytotoxicity, upregulates antimicrobial effector proteins, and restores functional responses even in patients with impaired MAIT cell pools; importantly, secretomes from activated MAIT cells were shown to resensitize carbapenem-resistant Escherichia coli carrying blaNDM-1, blaKPC-2, and blaOXA-48 by restoring imipenem activity and reducing bacterial viability and growth, supporting the potential of MAIT cell–based immune augmentation as an adjunct strategy to combat multidrug-resistant bacterial infections.
- [Chowdhury A, Powell RE, Kennedy JN, Urbanek KL, Angus DC, Chang CCH, et al. Measuring signatures of host resistance, disease tolerance, and damage in human sepsis: a prospective cohort study. Intensive Care Med. 2026 Apr 7; doi:10.1007/s00134-026-08404-7](https://ad-id.co/4sBETnP)
  - Summary: In this prospective study of 444 adults with community-onset Sepsis-3 sepsis enrolled in the emergency department, 16 plasma and urinary biomarkers were mapped through expert consensus into three mechanistic axes—host resistance to infection, disease tolerance, and host damage—and analyzed as composite signatures using principal component analysis, revealing that higher host damage was independently associated with increased 90-day mortality (aOR 1.70, 95% CI 1.38–2.11, p<0.001), while resistance and tolerance were not significantly linked to mortality; additionally, biomarker profiles varied across SENECA-defined sepsis subtypes, with δ-type patients showing higher damage and lower tolerance and α-type patients showing the opposite pattern, highlighting biologically distinct sepsis endotypes with prognostic relevance.

**Diagnostics**

- [Rodriguez-Temporal D, Gutiérrez-Pareja M, Gordy GG, Nahkala EM, Rodríguez-Sánchez B, Patel R. Prediction of KPC-producing *Klebsiella pneumoniae* by MALDI-TOF MS, ensemble learning, and spectral peak annotation. J Clin Microbiol. 2026 Mar 30; doi:10.1128/jcm.01466-25](https://ad-id.co/3QDCD27)
  - Summary: This multicenter study of 435 Klebsiella pneumoniae clinical isolates from multiple global regions evaluates an approach combining matrix-assisted laser desorption/ionization time-of-flight mass spectrometry (MALDI-TOF MS) with ensemble machine learning to improve detection of KPC carbapenemase production, demonstrating that 92 tested classifiers achieved high specificity (>95%) but moderate sensitivity (up to 72%), outperforming standard MALDI Biotyper KPC modules in specificity but limited by spectral variability affecting sensitivity; additionally, the study introduces the first in silico–annotated MALDI-TOF MS spectral profile of K. pneumoniae, providing peak-level interpretation that may support future antimicrobial resistance detection and clinical decision-making.
- [Dai Q, Lai L, Zhu Q, Yuan L. Clinical efficacy of plasma cell-free DNA metagenomic next-generation sequencing in diagnosing bloodstream infections. BMC Infect Dis. 2026 Mar 21; doi:10.1186/s12879-026-13134-8](https://ad-id.co/41AEsiL)
  - Summary: This retrospective study of 425 patients evaluated plasma cell-free DNA metagenomic next-generation sequencing (cfDNA mNGS) for infection diagnosis alongside conventional microbiological tests (CMTs), finding that mNGS demonstrated higher sensitivity (72.8% vs. 32.9%) but lower specificity (75.4% vs. 85.5%) compared with CMTs, with bloodstream infections being the most common diagnosis and Klebsiella pneumoniae, Candida albicans, and human cytomegalovirus among the most frequently detected pathogens; importantly, mNGS results influenced clinical management in a substantial proportion of cases (44.2% of positives and additional changes in mNGS-negative cases), were associated with higher detection rates in empirically treated patients, and showed that earlier sampling (days 1–3) was linked to shorter hospital stays, supporting cfDNA mNGS as a complementary tool to conventional diagnostics for guiding antimicrobial therapy and improving clinical outcomes.
- [Franklin S, Sahasrabhojane P, Hayase T, Hayase E, Chang CC, Senapati J, et al. Short-chain fatty acid-producing microbes differentiate non-infectious and infectious neutropenic fever in leukemia. mSystems. 2026 Mar 31; doi:10.1128/msystems.01343-25](https://ad-id.co/4sxLPCI)
  - Summary: This study of acute myeloid leukemia patients undergoing chemotherapy investigates whether gut microbiome and fecal metabolite profiles can distinguish infectious from non-infectious neutropenic fever using machine learning, showing that microbial signatures at baseline (including higher Akkermansia, Enterobacter, Escherichia–Shigella, and Flavonifractor in infectious cases and Collinsella, Lachnospiraceae, Coprococcus, and acetic acid in non-infectious cases) achieved moderate predictive performance (AUROC 0.769), while profiles at fever onset (e.g., enrichment of Enterococcus in infectious cases versus butyrate-producing taxa such as Lachnospiraceae and Ruminococcaceae in non-infectious cases) also differentiated outcomes (AUROC 0.752), alongside differences in microbiome–metabolome network connectivity, suggesting that integrated microbial and metabolic biomarkers may help guide antimicrobial decision-making in neutropenic fever and reduce unnecessary antibiotic exposure.

**Improving Clinical Research**

- [Tran A, Granton D, Fan E, Rochwerg B. Incorporating non-randomized studies into critical care clinical practice guidelines. Am J Respir Crit Care Med. 2026 Apr;212(4):724-732 doi:10.1093/ajrccm/aamag014](https://ad-id.co/4sIcM6Q)
  - Summary: This methodological overview discusses the role of clinical practice guidelines in critical care and emphasizes the primacy of randomized controlled trials (RCTs) under the GRADE framework for establishing causal evidence, while acknowledging that RCTs often leave important gaps in subgroup effects, long-term outcomes, and real-world applicability due to recruitment constraints and population heterogeneity; it argues that when RCT evidence is insufficient, high-quality non-randomized studies of interventions (NRSI)—particularly those using target trial emulation with large, well-validated datasets, careful control of confounding, and prespecified protocols—can complement evidence synthesis by approximating causal inference, but stresses that such studies require rigorous design, transparency, preregistration, and cautious interpretation due to inherent risks of bias, unmeasured confounding, and limitations in dynamic critical care settings.

**General Interest**

- [Liu S, Hu D, Xu T, Yin J, Shan X, Xia J, et al. An emerging human eye disease is associated with aquatic virus zoonotic infection. Nat Microbiol. 2026 Mar 26;11:892-906; doi:10.1038/s41564-026-02266-x](https://ad-id.co/425RjJW)
  - Summary: This study identifies covert mortality nodavirus (CMNV), an aquatic-origin virus widely present in farmed and wild aquatic animals, as being associated with persistent ocular hypertensive viral anterior uveitis (POH-VAU) in humans, demonstrating viral infection in ocular tissues and seroconversion in 70 affected patients, with epidemiological analyses linking higher disease risk to frequent and severe exposure to aquatic animals—particularly unprotected handling and consumption of raw seafood accounting for 71.4% of cases—while experimental models further show that CMNV can infect mammalian cells and induce elevated intraocular pressure and ocular tissue damage in mice, suggesting a potential zoonotic spillover driving this emerging ocular disease.

1. The article summaries in this supplement were generated with assistance from ChatGPT (OpenAI) and reviewed and edited by the authors for accuracy and content. [↑](#footnote-ref-1)
